# Asymmetric Allylation Catalyzed by Chiral Phosphoric Acids: Stereoselective Synthesis of Tertiary Alcohols and a Reagent‐Based Switch in Stereopreference

**DOI:** 10.1002/adsc.202100037

**Published:** 2021-05-05

**Authors:** Mattia Lazzarotto, Peter Hartmann, Jakob Pletz, Ferdinand Belaj, Wolfgang Kroutil, Stefan E. Payer, Michael Fuchs

**Affiliations:** ^1^ University of Graz Institute of Chemistry, Bioorganic and Organic Chemistry Heinrichstrasse 28/II 8010 Graz Austria; ^2^ University of Graz Institute of Chemistry Inorganic Chemistry Schubertstraße 1/III 8010 Graz Austria

**Keywords:** asymmetric allylation, chiral phosphoric acid, organocatalysis, tertiary alcohols, organozinc reagents

## Abstract

The substrate scope of the asymmetric allylation with zinc organyls catalyzed by 3,3‐bis(2,4,6‐triisopropylphenyl)‐1,1‐binaphthyl‐2,2‐diyl hydrogenphosphate (TRIP) has been extended to non‐cyclic ester organozinc reagents and ketones. Tertiary chiral alcohols are obtained with ee's up to 94% and two stereogenic centers can be created. Compared to the previous lactone reagent the stereopreference switches almost completely, proving the fact that the nature of the organometallic compound is of immense importance for the asymmetry of the product.

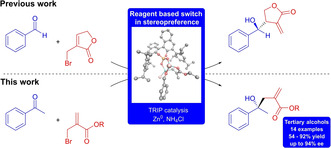

γ‐Butenolides and γ‐butyrolactones constitute a ubiquitous structural core in a plethora of natural products.^[1]^ These natural products and derivatives thereof have shown an immense therapeutic potential, ranging from anti‐inflammatory [e. g. kaliolide (**1**)]^[2]^ over antibiotic [e. g. paraconic acids (**2**)]^[3]^ to antitumor activity [e. g. hydroxymatairesinol (**3**),^[4]^ podophyllotoxin (**4**) derivatives,^[5]^ substituted α‐methylene γ‐butyrolactones;^[6]^ see Scheme 1].

**Scheme 1:**
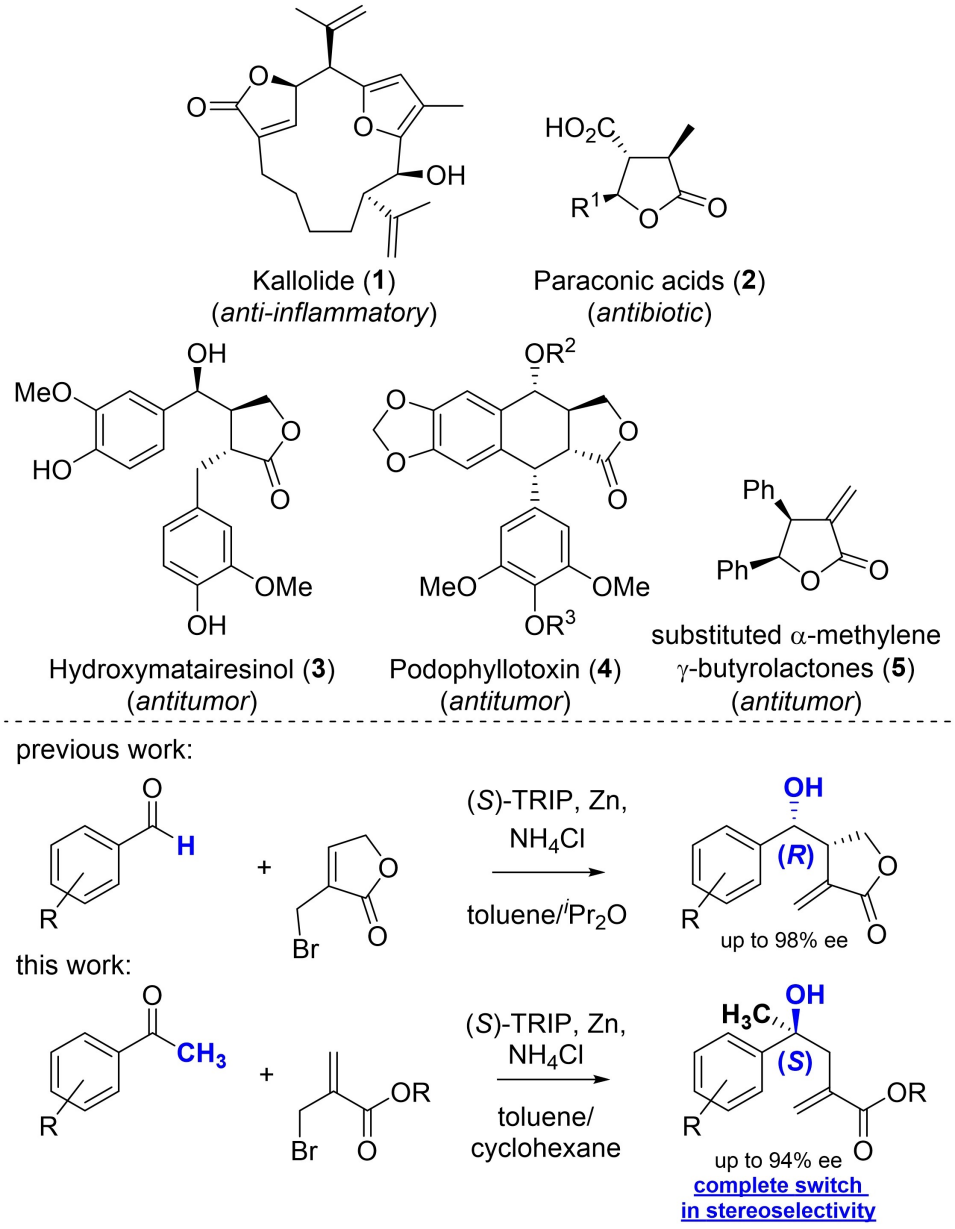
γ‐Butyrolactone natural products.

Based on this tremendous potential, synthetic methods to approach and generate these molecules are of great importance.^[7]^ Whereas preparation of the racemic material is well established – mostly by allylation/cyclization sequences – asymmetric induction on these reactions has proven difficult.^[8]^ Most methods base on less reactive organometallic precursors – most commonly allyl‐Sn, allyl‐Si or allyl‐B compounds. Several very versatile reaction protocols have been developed with regard to these reagents.^[9]^ Nevertheless, if it comes to functional group tolerance, esters are an essential feature of the allylating reagent in the context of butyrolactones and butenolides. With regard to the commonly used asymmetric protocols, at least one additional reaction step is required and very often the preceding preparation of the reagent is tedious and problematic, due to the functional group tolerance of the protocols at hand and/or the stability of the intermediates.[^8a^, ^9a^] Quite often, the activation of these compounds requires strong Lewis acid interactions during the asymmetric catalysis, which limits the functional group tolerance and the control over the reaction outcome.^[9a]^ We have recently reported on a mild, catalytic and asymmetric allylation of aldehydes based on a chiral phosphoric acid catalyst^[10]^ and organozinc compounds, which are formed *in situ* from the corresponding bromide.^[11]^ The developed method relies on readily available zinc dust and ammonium chloride for the generation of the reagent under the reaction conditions. These mild reagents allow insertion of the metal into the carbon‐halogen bond in the presence of esters in an apolar medium (e. g. toluene) – the requirement for asymmetric induction in the developed system.^[11]^ This facilitates the existing routes to natural products like lignans substantially.^[12]^ Additionally, the obtained products provide extremely versatile starting materials for a plethora of transformations [e. g., preparation of α‐keto esters,^[13]^ (stereoselective) reduction of the obtained double bond,^[14]^ conjugated 1,4‐addition of carbo‐^[15]^ and thia‐^[16]^ nucleophiles, Heck reactions,^[17]^ and double bond isomerization^[18]^].

Our studies started with the evaluation of different allylic bromides (see Table 1) under the previously reported reaction conditions.^[11]^ (*S*)‐3,3‐Bis(2,4,6‐triisopropylphenyl)‐1,1‐binaphthyl‐2,2‐diyl hydrogenphosphate [(*S*)‐TRIP, **9**] was used as catalyst.

**Table 1:** Reaction optimization – asymmetric allylation of benzaldehyde/acetophenone (**6 a**–**b**).

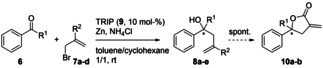					
Entry	R^1^	R^2^	t [h]	Conv. [%]^[a]^	Ee [%]^[b]^
1	H (**6 a**)	H (**7 a**)	2	>99 (**8 a**)	<1^[c]^
2	H (**6 a**)	CO_2_Et (**7 b**)	8	>99 (**10 a**)	50^[c]^
3	Me (**6 b**)	CO_2_Et (**7 b**)	16	>99 (**10 b**)	70^[c]^
4	Me (**6 b**)	CO_2_Et (**7 b**)	16	>99 (**10 b**)	75^[c,d]^
5	Me (**6 b**)	CO_2_Bn (**7 c**)	16	>99 (**8 d**)	77
6	Me (**6 b**)	CO_2_(CHPh_2_) (**7 d**)	16	>99 (89^[e]^, **8 e**)	89
7	Me (**6 b**)	CO(NBn_2_) (**7 f**)	16	33 (30^[e]^, **8 r**)	11

Reaction conditions: aldehyde or ketone (0.1 mmol), zinc (5 eq.), NH_4_Cl (8 eq.), (*S*)‐TRIP [(*S*)‐**9**, 10 mol‐%], **7 a**–**d** (1.5 eq.) in toluene (1 mL) and cyclohexane (1 mL); ^[a]^ Conversions were determined via HPLC‐UV (215 nm); the product is given in brackets. ^[b]^ The enantiomeric excess was determined on a chiral stationary phase via normal phase HPLC‐UV. ^[c]^ Spontaneous lactonization was observed leading to product **10 a**–**b**. ^[d]^ The reaction was performed at −20 °C. ^[e]^ Isolated yield.

Simple allyl bromide (**7 a**) gave racemic product **8 a** upon reaction with benzaldehyde (**6 a**, Table 1, entry 1). The change to the less reactive acrylate **7 b** increased the ee‐value to 50% (Table 1, entry 2). By the assumption that the unwanted enantiomer was formed by the uncatalyzed background reaction, we intended to decrease the reactivity of the electrophile by the change from aldehydes to ketones. Indeed, acetophenone (**6 b**) reacted with an increased enantioselectivity yielding 70% ee (Table 1, entry 3). Lowering the reaction temperature to −20 °C increased the enantiomeric excess slightly (75%, Table 1, entry 4).

Tuning the size of the ester group led to an increase by 7% ee for the benzyl congener and using benzhydryl acrylates further improved the ee to 89% with full conversion after 16 h (Table 1, entries 5 and 6). Important to note is that low but significant racemization was observed for the acidic lactonization. Therefore, the spontaneous lactonization in case of entries 3–5 (Table 1) may cause slightly reduced ee values. Complete suppression of this spontaneous follow up reaction was only possible in the case of reagent **7 d**. This racemization may also be the reason for the slightly improved ee at lower temperatures in the case of reagent **7 b** (see Table 1, entry 4), as a beneficial effect of lower reaction temperatures was not observed for entry 6 and reagent **7 d** (see Table 1).

Amide congeners of reagent **7** provided the allylation product **8 r** only in poor conversion (33%, 30% isolated yield) and low enantiomeric excess (11% ee; see Table 1, entry 7). The lower reactivity may be attributed to the higher stabilization of the organozinc reagent (better coordination of the amide).

Consequently, several commonly used chiral phosphoric acids (CPAs) were screened under the reaction conditions (see Table 2). Whereas Vapol hydrogenphosphate (**11**) gave racemic product **10 b** (Table 2, entry 1), CPAs **12**, **13**, and **15** induced low enantioselectivity in the reaction (Table 2, entries 2–4). The anthracenyl‐derivative **14** increased this induction two‐fold (Table 2, entry 5) and, as observed in the preliminary study, TRIP (**9**) gave the best results (Table 2, entry 6).

**Table 2:** Screening of different chiral phosphoric acid (CPA) catalysts.

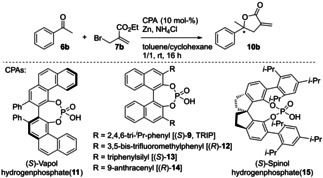			
Entry	Chiral Phosphoric Acid (CPA)	Conv. [%]^[a]^	ee [%]^[b]^
1	(*S*)‐**11**	>99	<1
2	(*R*)‐**12**	>99	17^[c]^
3	(*S*)‐**13**	>99	27
4	(*S*)‐**15**	>99	33^[d]^
5	(*R*)‐**14**	>99	44^[c]^
6	(*S*)‐**9**	>99	70

Reaction conditions: ketone (0.1 mmol), zinc (5 eq.), NH_4_Cl (8 eq.), CPA (10 mol‐%), **7 b** (1.5 eq.) in toluene (1 mL) and cyclohexane (1 mL); spontaneous lactonization to product **10 b** was observed for all entries. ^[a]^ Conversions were determined via HPLC‐UV (215 nm). ^[b]^ The enantiomeric excess was determined on a chiral stationary phase via normal phase HPLC‐UV (215 nm). ^[c]^ The opposite enantiomer was observed [note: the (*R*)‐CPA has been used in case of these entries]. ^[d]^ The opposite enantiomer was observed [note: reagent **7d** was used in case of this entry].

Important to note is, that no reaction of acetophenone with allylboronic acid pinacol ester (**16**) was observed under the reaction conditions published for aldehydes.^[19]^ In addition, we conducted a cross experiment, where we added acetophenone to a 1:1 mixture of the organozinc species formed in‐situ from **7 b** and allylboronic acid pinacol ester (**16**) under TRIP (**9**) catalysis. We were able to detect the product formed from the reaction with **7 b** only and no allylation reaction took place with the allylboronic ester **16** (see Scheme 2). These two experiments indicate, that the zinc organyl is by far more reactive than the boronate congener under the same reaction conditions, and thus enables the allylation of the less reactive ketones.

**Scheme 2:**
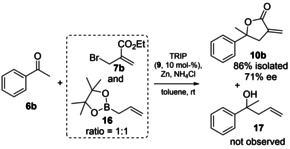
Cross experiment with allylboronic ester **16**.

The next step was to test different ketone substrates under the optimized reaction conditions. The results are summarized in Table 3. Compared to substrate **6 b**, *para*‐substituted acetophenones gave comparable results (see Table 3, entry 1 vs. entries 2–4). Going from *para*‐ to *meta*‐ and *ortho*‐substitution decreased the enantiomeric excess and showed also slight drops in the isolated yields (Table 3, entries 4–6). Interestingly, the non‐aromatic ketone residue showed a beneficial effect on the reaction outcome upon increasing its size (Table 3, entries 1, 7 and 8). The bigger sidechain did not interfere with the isolated yield but increased the ee to >90%. The same effect was observed with ketones of higher rigidity (e. g. 1‐tetralone and chromanone, see Table 3, entries 9 and 10). As expected and observed previously,^[11]^ ketones with a non‐cyclic residue performed poor with regard to the asymmetric induction, even when an aromatic residue is present in higher distance to the reacting electrophilic site (Table 3, entry 12).

**Table 3:** TRIP‐catalyzed allylation of different ketones under optimized conditions.

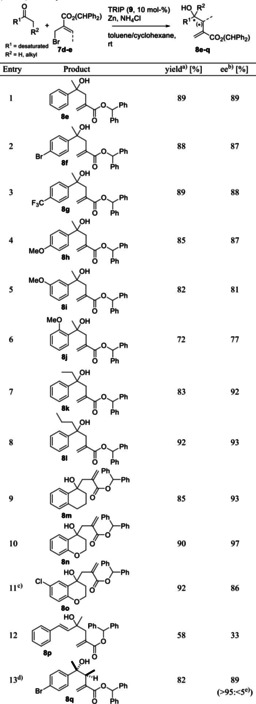

Reaction conditions: Ketone (0.1 mmol), zinc (5 eq.), NH_4_Cl (8 eq.), (*S*)‐TRIP [(*S*)‐**9**, 10 mol‐%], **7 d** (1.5 eq.), 0.5–2 mL toluene/cyclohexane mixture (for details see ESI); stirred at rt for 16 h. ^[a]^ Isolated yields are given. ^[b]^ The enantiomeric excess was determined on a chiral stationary phase via normal phase HPLC‐UV. ^[c]^ (*R*)‐TRIP [(*R*)‐**9**, 10 mol‐%] was used. ^[d]^ Benzhydryl (*Z*)‐2‐(bromomethyl)but‐2‐enoate (**7 e**) was used as allylating reagent. ^[e]^ The diastereomeric ratio (d.r.) was determined via ^1^H‐NMR (given in brackets); relative stereoconfiguration was determined by ^1^H,^1^H‐NOESY spectra of lactone **10 q** (see Scheme 3 and the ESI).

Using benzhydryl (*Z*)‐2‐(bromomethyl)but‐2‐enoate (**7 e**) as allylating reagent, 4‐bromoacetophenone was smoothly converted into ester **8 q**, with full control over the relative stereochemistry and with 89% ee (see Table 3, entry 13).

In order to determine the absolute configuration of the obtained homoallylic alcohols, we compared the optical rotation values for lactonized **10 b** to its literature values (see Scheme 3a).^[20]^ Again, slight racemization was observed under the acidic conditions of the lactonization and the ee value dropped from 89 to 72%. Whereas in the initial report (*S*)‐TRIP afforded the (*R*)‐configured alcohol,^[11]^ the optical rotation values show a complete switch of the stereopreference with the ester allylating reagents **7 b**–**e** and the (*S*)‐catalyst yields the (*S*)‐alcohol.

**Scheme 3:**
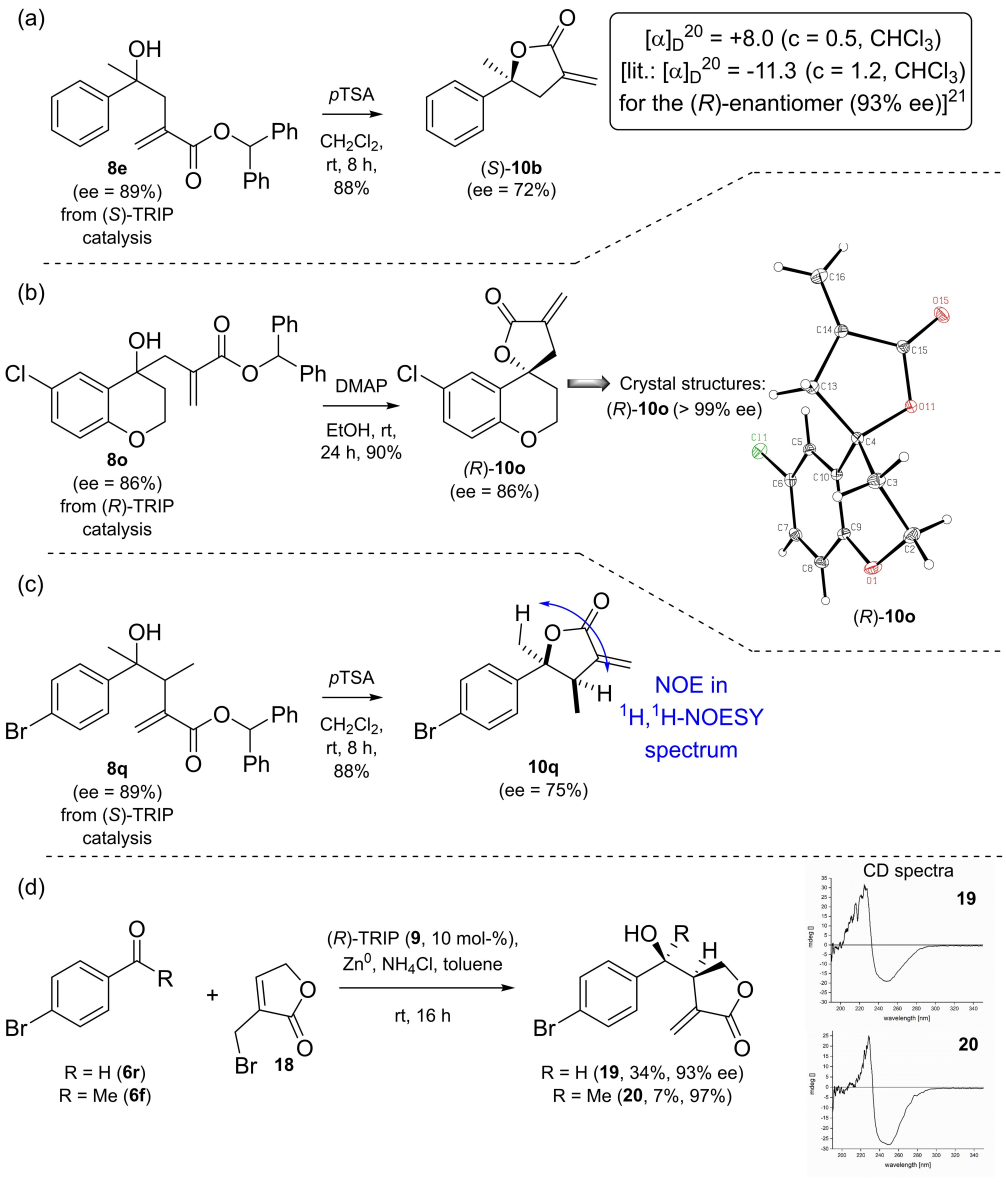
Determination of the absolute and relative configuration.

As the optical rotation values are low and even switch from plus to minus depending on the solvent,^[20]^ we lactonized compound **8 o** and crystallized the corresponding lactone **10 o** (see Scheme 3).

Under acidic conditions we observed complete racemization of the tertiary alcohol. Nevertheless, we were able to pick two crystals from the sample, which provided enantioenriched material [ee=50 and 98% for the (*S*)‐enantiomer] and whose structures were determined by the means of X‐ray diffraction (for details see ESI). Additionally we found that under basic conditions **8 o** lactonizes without any racemization.^[21]^ By crystallization of the obtained lactone (*R*)‐**10 o** and resubjecting the single crystals from the X‐ray analyses to HPLC‐UV analysis on a chiral stationary phase we were able to confirm the switch in enantiopreference of the catalyst compared to the initial report (see Scheme 3b and ESI). In order to identify the origin of the switch we reacted 4‐bromoacetophenone with the lactone based reagent **18** from our initial report (see Scheme 3d).^[11]^ The comparison to the same reaction with 4‐bromobenzaldehyde gave the same Cotton effects in the CD spectra. Thus, the switch in stereopreference finds its origin in the nature of the organozinc reagent.

A rationale of this remarkable switch in stereoselectivity may be found in the higher flexibility of the open‐chained reagent. Therefore the dipole moments of the reagent^[22]^ become less dominating and steric factors, reflected in the size of the ester group become stereo‐determining. Nevertheless, these assumptions just demonstrate the working basis of more detailed calculations currently performed in our laboratories.

This switch in stereoselectivity with the same catalyst represents an intriguing feature. By the use of the open‐chain reagent **7 b** the same stereopreference of the catalytic reaction is observed as for the congeneric allylation using allylboronate reagent **15**.^[19]^ In a mechanistic context, this fact renders a highly similar reaction outcome and increases the chemical proximity of both reactions. Further studies on the origin of this switch in stereopreference are on the way and will be reported in due course.

In general, we have been able to extend the substrate scope of the zinc mediated, TRIP‐catalyzed allylation reaction to ketones. The formed chiral, tertiary alcohols are obtained with high enantiopurity and up to two stereocenters can be generated with full stereocontrol. The allylating reagent still requires a carbonyl function nearby the zinc insertion site, most probably to stabilize the reagent and make it less reactive and thus accessible to the timeframe of the catalytic reaction. The observed switch in stereopreference increases the proximity of the outlined reaction to its boron congener using the same catalyst, as both reactions provide the same stereochemical outcome for open‐chain reagents. Further studies are required to evaluate if these phenomena are just a coincident or if the reactions are more similar than initially anticipated. These studies are currently performed in our laboratories.

## Experimental Section

### General Procedure for the Asymmetric Allylation of Ketones (Procedure C)

A 5 mL screw cap vial was charged with zinc (33.0 mg, 500 μmol, 5 eq.), NH_4_Cl (43.0 mg, 800 μmol, 8 eq.) and (*S*)‐3,3′‐bis(2,4,6‐triisopropylphenyl)‐1,1′‐binaphthyl‐2,2′‐diyl hydrogenphosphate (TRIP, 7.5 mg, 10.0 μmol, 0.1 eq.) followed by the respective solvent mixture. The ketone (100 μmol) and allyl bromide **7 d** (50.0 mg, 150 μmol, 1.5 eq.) were added [in case of product **8 q** benzhydryl (Z)‐2‐(bromomethyl)but‐2‐enoate (**7 e**, 52.0 mg, 150 μmol, 1.5 eq.). The mixture was stirred (720 rpm) at room temperature for 16 h, quenched by the addition of NH_4_Cl_sat., aq._ solution (5 mL) and extracted with EtOAc (3×10 mL). The combined organic phase was dried over Na_2_SO_4_, filtered and concentrated under reduced pressure. The obtained crude product was purified via flash chromatography (SiO_2_, eluent is indicated below) to give the pure product.

Detailed experimental procedures, NMR‐ and HPLC‐spectra (PDF) and determination of the crystal structures can be found in the supporting information.

CCDC 1944605–1944607 contain the supplementary crystallographic data for this paper [(*S*)‐**10 o**‐Cry1, (*S*)‐**10 o**‐Cry2 and (*R*)‐**10 o**)]. These data can be obtained free of charge via http://www.ccdc.cam.ac.uk/conts/retrieving.html (or from the CCDC, 12 Union Road, Cambridge CB2 1EZ, UK; Fax: +44 1223 336033; E‐mail: deposit@ccdc.cam.ac.uk).

## Supporting information

As a service to our authors and readers, this journal provides supporting information supplied by the authors. Such materials are peer reviewed and may be re‐organized for online delivery, but are not copy‐edited or typeset. Technical support issues arising from supporting information (other than missing files) should be addressed to the authors.

Supplementary
