# Supplementary material for: Asymmetric Allylation Catalyzed by Chiral Phosphoric Acids: Stereoselective Synthesis of Tertiary Alcohols and a Reagent‐Based Switch in Stereopreference
Source: Adv Synth Catal. 2021 May 5;363(12):3138–43. doi: 10.1002/adsc.202100037 (PMC8359974; doi:10.1002/adsc.202100037)
Supplement: Supplementary file 1 — Supplementary [file ADSC-363-3138-s001.pdf]

Advanced Synthesis & Catalysis

Supporting Information

Asymmetric Allylation Catalyzed by Chiral Phosphoric Acids: Stereoselective Synthesis of Tertiary Alcohols and a Reagent-Based Switch in Stereopreference

Mattia Lazzarotto,[+] Peter Hartmann,[+] Jakob Pletz, Ferdinand Belaj, Wolfgang Kroutil, Stefan E. Payer, and Michael Fuchs*© 2021 The Authors. Advanced Synthesis & Catalysis published by Wiley-VCH GmbH. This is an open access article under the terms of the Creative Commons Attribution License, which permits use, distribution and reproduction in any medium, provided the original work is properly cited.

Supporting Information -

Asymmetric Allylation Catalysed by Chiral Phosphoric Acids: Stereoselective Synthesis of Tertiary Alcohols and a Reagent-Based Switch in Stereopreference

Mattia Lazzarotto,[a),⊥] Peter Hartmann,[a),⊥] Jakob Pletz,[a)] Ferdinand Belaj,[b)] Wolfgang Kroutil,[a)] Stefan E. Payer,[a)] Michael Fuchs[a),*].

[a)] University of Graz, Institute of Chemistry, Bioorganic and Organic Chemistry, Heinrichstrasse 28/II, 8010 Graz, Austria, Europe.

[b)] University of Graz, Institute of Chemistry, Inorganic Chemistry, Schubertstraße 1/III, 8010 Graz, Austria, Europe.

Table of contents

Table of contents S2

General Information........ S6

Instrumentation S6

Experimentals. S7

Benzyl acrylate (**SI01**). S7

Benzyl 2-(bromomethyl)acrylate (**7c**). S7

Benzhydryl acrylate (**SI02**)........ S7

Benzhydryl 2-(hydroxymethyl)acrylate (**SI03**). S7

Benzhydryl 2-(bromomethyl)acrylate (**7d**). S8

Benzhydryl 3-hydroxy-2-methylenebutanoate (**SI04**). S8

Benzhydryl (*Z*)-2-(bromomethyl)but-2-enoate (**7e**)........ S8

Synthesis of substitued acrylamides........ S9

N,*N*-diethylacrylamide (**SI5**). S9

N,*N*-dibenzylacrylamide (**SI6**). S9

Baylis-Hillman reaction of substitued acrylamides. S9

N,*N*-diethyl-2-(hydroxymethyl)acrylamide (**SI7**)........ S9

N,*N*-dibenzyl-2-(hydroxymethyl)acrylamide (**SI8**). S10

N,*N*-diethyl-2-(bromomethyl)acrylamide (**SI9**)........ S10

N,*N*-dibenzyl-2-(bromomethyl)acrylamide (**7f**). S10

General procedure for the asymmetric allylation of ketones (Procedure A). S11

1-Phenylbut-3-en-1-ol (**8a**)........ S11

3-Methylene-5-phenyldihydrofuran-2(3*H*)-one (**10a**). S11

5-Methyl-3-methylene-5-phenyldihydrofuran-2(3*H*)-one (**10b**). S11

Benzyl 4-hydroxy-2-methylene-4-phenylpentanoate (**8d**). S12

General procedure for the preparation of racemic reference material (Procedure B)........ S12

General procedure for the asymmetric allylation of ketones (Procedure C). S12

Benzhydryl 4-hydroxy-2-methylene-4-phenylpentanoate (**8e**). S12

Benzhydryl 4-(4-bromophenyl)-4-hydroxy-2-methylenepentanoate (**8f**)........ S13

Benzhydryl 4-hydroxy-2-methylene-4-[4-(trifluoromethyl)phenyl]pentanoate (**8g**). S13

Benzhydryl 4-hydroxy-4-(4-methoxyphenyl)-2-methylenepentanoate (**8h**). S13

Benzhydryl 4-hydroxy-4-(3-methoxyphenyl)-2-methylenepentanoate (**8i**)........ S14

Benzhydryl 4-hydroxy-4-(2-methoxyphenyl)-2-methylenepentanoate (**8j**)........ S14

Benzhydryl 4-hydroxy-2-methylene-4-phenylhexanoate (**8k**). S14

Benzhydryl 4-hydroxy-2-methylene-4-phenylheptanoate (**8l**). S15

Benzhydryl 2-[(1-hydroxy-1,2,3,4-tetrahydronaphthalen-1-yl)methyl]acrylate (**8m**)........ S15

Benzhydryl 2-[(4-hydroxychroman-4-yl)methyl]acrylate (**8n**). S15

Benzhydryl 2-[(6-chloro-4-hydroxychroman-4-yl)methyl]acrylate (**8o**)....S16
Benzhydryl (*E*)-4-hydroxy-4-methyl-2-methylene-6-phenylhex-5-enoate (**8p**). S16
Benzhydryl 4-(4-bromophenyl)-4-hydroxy-3-methyl-2-methylenepentanoate (**8q**). S16
General procedure for the asymmetric allylation of ketones with amide reagents **SI9** and **SI10** (Procedure D). S17
N,*N*-dibenzyl-4-hydroxy-2-methylene-4-phenylpentanamide (**8r**). S17
N,*N*-diethyl-4-hydroxy- 2-methylene-4-phenylpentanamide (**SI12**). S17
Determination of absolute configuration and diastereoselectivity S17
(*S*)-5-Methyl-3-methylene-5-phenyldihydrofuran-2(3*H*)-one (**10b**). S17
Preparation of (*R*)-6-chloro-4'-methylene-3',4'-dihydro-5'H-spiro[chromane-4,2'-furan]-5'-one (**10o**) with DMAP. S18
Preparation of *rac*-6-chloro-4'-methylene-3',4'-dihydro-5'H-spiro[chromane-4,2'-furan]-5'-one (**10o**) with *p*TSA. S18
Crystallization of **10o**. S18
5-(4-bromophenyl)-4,5-dimethyl-3-methylenedihydrofuran-2(3H)-one (**10q**). S19
Investigations of the origin of the switch and the responsible structural motif S19
General procedure for the asymmetric allylation of ketones with the lactone based organozinc reagent. S19
4-[(4-bromophenyl)(hydroxy)methyl]-3-methylenedihydrofuran-2(3*H*)-one (**19**). S20
4-[1-(4-bromophenyl)-1-hydroxyethyl]-3-methylenedihydrofuran-2(3*H*)-one (**20**). S20
CD spectra of compounds **19** and **20**. S21
Crystal Structure Determination of **10o**. S21
rac-**10o**-Cry1 ....S21
rac-**10o**-Cry2 ....S27
(*R*)-**10o** S33
NMR data S39
Benzyl acrylate (**SI01**) in $CDCl_3$ @ 300.13 MHz (^{1}H) and 75. 47 MHz (^{13}C). S40
Benzhydryl 2-(hydroxymethyl)acrylate (**SI03**) in $CDCl_3$ @ 300.13 MHz (^{1}H) and 75. 47 MHz (^{13}C). S41
Benzhydryl 2-(bromomethyl)acrylate (**7d**) in $CDCl_3$ @ 300.13 MHz (^{1}H) and 75. 47 MHz (^{13}C). S42
Benzhydryl 3-hydroxy-2-methylenebutanoate (**SI04**) in $CDCl_3$ @ 300.13 MHz (^{1}H) and 75.47 MHz (^{13}C). S43
Benzhydryl (Z)-2-(bromomethyl)but-2-enoate (**7e**) in $CDCl_3$ @ 300.13 MHz (^{1}H) and 75. 47 MHz (^{13}C). S44
N,*N*-diethyl-2-(hydroxymethyl)acrylamide (**SI7**) S46
N,*N*-dibenzyl-2-(hydroxymethyl)acrylamide (**SI8**). S47
N,*N*-diethyl-2-(bromomethyl)acrylamide (**SI9**). S48
N,*N*-dibenzyl-2-(bromomethyl)acrylamide (**7f**). S49

1-Phenylbut-3-en-1-ol (**8a**) in $CDCl_3$ @ 300.13 MHz (^{1}H) and 75. 47 MHz (^{13}C). S50

3-Methylene-5-phenyldihydrofuran-2(3*H*)-one (**10a**) in $CDCl_3$ @ 300.13 MHz (^{1}H) and 75. 47 MHz (^{13}C). S51

5-Methyl-3-methylene-5-phenyldihydrofuran-2(3*H*)-one (**10b**) in $CDCl_3$ @ 300.13 MHz (^{1}H) and 75. 47 MHz (^{13}C). S52

Benzhydryl 4-hydroxy-2-methylene-4-phenylpentanoate (**8e**) in $CDCl_3$ @ 300.13 MHz (^{1}H) and 75. 47 MHz (^{13}C). S53

Benzhydryl 4-(4-bromophenyl)-4-hydroxy-2-methylenepentanoate (**8f**) in $CDCl_3$ @ 300.13 MHz (^{1}H) and 75. 47 MHz (^{13}C). S54

Benzhydryl 4-hydroxy-2-methylene-4-[4-(trifluoromethyl)phenyl]pentanoate (**8g**) in $CDCl_3$ @ 300.13 MHz (^{1}H), 282.39 MHz (^{19}F) and 75. 47 MHz (^{13}C). S55

Benzhydryl 4-hydroxy-4-(4-methoxyphenyl)-2-methylenepentanoate (**8h**) in $CDCl_3$ @ 300.13 MHz (^{1}H) and 75. 47 MHz (^{13}C). S57

Benzhydryl 4-hydroxy-4-(3-methoxyphenyl)-2-methylenepentanoate (**8i**) in $CDCl_3$ @ 300.13 MHz (^{1}H) and 75. 47 MHz (^{13}C). S58

Benzhydryl 4-hydroxy-4-(2-methoxyphenyl)-2-methylenepentanoate (**8j**) in $CDCl_3$ @ 300.13 MHz (^{1}H) and 75. 47 MHz (^{13}C). S59

Benzhydryl 4-hydroxy-2-methylene-4-phenylhexanoate (**8k**) in $CDCl_3$ @ 300.13 MHz (^{1}H) and 75. 47 MHz (^{13}C). S60

Benzhydryl 4-hydroxy-2-methylene-4-phenylheptanoate (**8l**) in $CDCl_3$ @ 300.13 MHz (^{1}H) and 75. 47 MHz (^{13}C). S61

Benzhydryl 2-[(1-hydroxy-1,2,3,4-tetrahydronaphthalen-1-yl)methyl]acrylate (**8m**) in $CDCl_3$ @ 300.13 MHz (^{1}H) and 75. 47 MHz (^{13}C). S62

Benzhydryl 2-[(4-hydroxychroman-4-yl)methyl]acrylate (**8n**) in $CDCl_3$ @ 300.13 MHz (^{1}H) and 75. 47 MHz (^{13}C). S63

Benzhydryl 2-[(6-chloro-4-hydroxychroman-4-yl)methyl]acrylate **(8o)** in $CDCl_3$ @ 300.13 MHz (^{1}H) and 75. 47 MHz (^{13}C). S64

Benzhydryl (*E*)-4-hydroxy-4-methyl-2-methylene-6-phenylhex-5-enoate (**8p**) in $CDCl_3$ @ 300.13 MHz (^{1}H) and 75. 47 MHz (^{13}C). S65

Benzhydryl 4-(4-bromophenyl)-4-hydroxy-3-methyl-2-methylenepentanoate (**8q**) in $CDCl_3$ @ 300.13 MHz (^{1}H) and 75. 47 MHz (^{13}C). S66

(*R*)-6-chloro-4'-methylene-3',4'-dihydro-5'H-spiro[chromane-4,2'-furan]-5'-one **(10o)** in $CDCl_3$ @ 300.13 MHz (^{1}H) and 75. 47 MHz (^{13}C). S67

5-(4-bromophenyl)-4,5-dimethyl-3-methylenedihydrofuran-2(3H)-one (**10q**) in $CDCl_3$ @ 300.13 MHz (^{1}H) and 75. 47 MHz (^{13}C). S68

4-[(4-bromophenyl)(hydroxy)methyl]-3-methylenedihydrofuran-2(3*H*)-one (**19**) in $CDCl_3$ @ 300.13 MHz (^{1}H) and 75. 47 MHz (^{13}C). S70

4-[1-(4-bromophenyl)-1-hydroxyethyl]-3-methylenedihydrofuran-2(3*H*)-one (**20**) in $CDCl_3$ @ 300.13 MHz (^{1}H) and 75. 47 MHz (^{13}C). S71

HPLC chromatograms S73

Benzhydryl 4-hydroxy-2-methylene-4-phenylpentanoate (**8e**). S74

Benzhydryl 4-(4-bromophenyl)-4-hydroxy-2-methylenepentanoate (**8f**). S75

Benzhydryl 4-hydroxy-2-methylene-4-[4-(trifluoromethyl)phenyl]pentanoate (**8g**). S76
Benzhydryl 4-hydroxy-4-(4-methoxyphenyl)-2-methylenepentanoate (**8h**). S77
Benzhydryl 4-hydroxy-4-(3-methoxyphenyl)-2-methylenepentanoate (**8i**)........................ S78
Benzhydryl 4-hydroxy-4-(2-methoxyphenyl)-2-methylenepentanoate (**8j**)........................ S79
Benzhydryl 4-hydroxy-2-methylene-4-phenylhexanoate (**8k**). S80
Benzhydryl 4-hydroxy-2-methylene-4-phenylheptanoate (**8l**). S81
Benzhydryl 2-[(1-hydroxy-1,2,3,4-tetrahydronaphthalen-1-yl)methyl]acrylate (**8m**)......... S82
Benzhydryl 2-[(4-hydroxychroman-4-yl)methyl]acrylate (**8n**). S83
Benzhydryl 2-[(6-chloro-4-hydroxychroman-4-yl)methyl]acrylate **(8o)**............................. S84
Benzhydryl (*E*)-4-hydroxy-4-methyl-2-methylene-6-phenylhex-5-enoate (**8p**). S85
Benzhydryl 4-(4-bromophenyl)-4-hydroxy-3-methyl-2-methylenepentanoate (**8q**). S86
Determination of Absolute Configuration. S87
5-Methyl-3-methylene-5-phenyldihydrofuran-2(3H)-one (**10b**)................................... S87
(R)-6-chloro-4'-methylene-3',4'-dihydro-5'H-spiro[chromane-4,2'-furan]-5'-one **(10o)**. S88
HPLC chromatograms of single crystals from X-Ray Analysis. S89
5-(4-bromophenyl)-4,5-dimethyl-3-methylenedihydrofuran-2(3H)-one (**10q**). S91
4-[(4-bromophenyl)(hydroxy)methyl]-3-methylenedihydrofuran-2(3*H*)-one (**19**). S92
4-[1-(4-bromophenyl)-1-hydroxyethyl]-3-methylenedihydrofuran-2(3H)-one (**20**). S93
References. S95

General Information.

All chemicals were purchased from Sigma Aldrich or Acros Organics and were used as received, unless otherwise noted. All solvents were purchased from Roth, except Dioxane (Alfa Aasar) and dry toluene (Sigma Aldrich). Moisture sensitive reactions were performed using standard Schlenk techniques with argon 5.0.

Analytical thin layer chromatography (TLC) was carried out on Merck TLC silica gel aluminum sheets (silica gel 60, F_{254}, 20 x 20 cm) and spots were visualized by UV light (λ = 254 nm) and by staining with cerium ammonium molybdate solution (50 g $(NH_4)_6Mo_7O_{24}$ were dissolved in 400 mL H_2O and 50 mL conc. H_2SO_4 was added followed by 2.0 g $Ce(SO_4)_2$) or $KMnO_4$ solution (1 g $KMnO_4$ and 2 g Na_2CO_3 were dissolved in 100 mL H_2O) and developed by heating with a heat gun.

Column chromatography was performed on silica gel 60 from Merck with particle sizes 40-63 µm. A 30- to 100-fold excess of silica gel was used with respect to the mass of dry crude product, depending on the separation problem. For sticky crude products, the crude material was dissolved in MeOH and subsequently adsorbed on the 2.5-fold excess of silica gel. Afterwards the solvent was removed in vacuum and the adsorbed crude material was dried in oil pump vacuum. The dimension of the column was adjusted to the required amount of silica gel and formed a pad between 20 and 40 cm of height. In general, the silica gel was mixed with the eluent and charged into the column before equilibration. Subsequently, the dissolved or adsorbed crude material was loaded onto the top of the silica gel and the mobile phase was forced through the column by pressure exerted by a rubber bulb pump.

Instrumentation

^{1}H-, ^{13}C-, ^{31}P- and ^{11}B-NMR spectra were recorded on a Bruker AVANCE III 300 spectrometer (^{1}H: 300.13 MHz; ^{13}C: 75.47 MHz; ^{31}P: 121.49 MHz; ^{11}B: 96.29) with autosampler. Chemical shifts were referenced to the residual proton and carbon signal of the deuterated solvent [$CDCl_3$: δ = 7.26 ppm (^{1}H), 77.16 ppm (^{13}C)]. Chemical shifts δ are given in ppm (parts per million) and coupling constants J in Hz (Hertz). Deuterated solvents for nuclear resonance spectroscopy were purchased from Roth.

Melting points were determined on a Gallenkamp MPD350.BM2.5 apparatus with an integrated microscopical support. They were measured in open capillary tubes with a mercury-in-glass thermometer and were not corrected.

IR-spectra were recorded neat on a Bruker Alpha-P (ATR) instrument.

The specific optical rotation was determined on a Perkin Elmer Polarimeter 341 with an integrated sodium vapor lamp. All samples were measured in $CHCl_3$ and CH_2Cl_2 (both were purchased from Sigma Aldrich, ACS specrophotometric grade, ≥99.8%) at the D-line of the sodium light (λ = 589 nm) under non-tempered conditions between 22 °C and 27 °C.

High resolution mass spectra were recorded on an Agilent 6230 TOF LC/MS using ESI (positive mode, capillary voltage 3.5 kV) or APCI (negative mode, 5.0 kV) methods.

Chiral HPLC analysis was performed on a Shimadzu HPLC system [DGU-20A (degasser), LC-20A (pump), SIL-20A (autosampler), CTO-20AC (column oven), SPD-M20A (detector), CBM-20AC (controller)] with *n*-heptane/2-PrOH as eluent using a Daicel columns [dimension: 4.6 x 250 mm, 5 µm particle size, except Chiralpak AD (10 µm) and Chiralcel OJ (10 µm)] and conditions as specified below.

Experimentals.

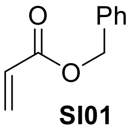

SI01

Benzyl acrylate (SI01).

Acrylic acid (2.16 g, 30.0 mmol, 1 eq.) was dissolved in DMF (20 mL) and cooled to 0 °C with an ice bath. K_2CO_3 (4.15 g, 30.0 mmol, 1 eq.) was added portionwise over 5 min and the mixture was stirred for 20 min. The ice bath was removed and benzyl bromide (5.13 g, 30.0 mmol, 3.56 mL, 1 eq.) was added and the mixture was heated to 85 °C (oil bath temperature) and kept at that temperature for 16 h. The obtained slurry was cooled to room temperature and the reaction was quenched by water (ca. 20 mL). The mixture was extracted with hexanes (3 x 40 mL) and the combined organic phase was washed with saturated aqueous NH_4Cl solution, dried over Na_2SO_4, filtered and concentrated. The remaining crude product was purified via flash chromatography (silica gel, hexanes/EtOAc 10/1) to give the product as a clear oil (2.78 g, 17.0 mmol, 57%).

^{1}H-NMR (300.13 MHz, $CDCl_3$): 7.42-7.31 (m, 5H), 6.47 (dd, J_1 = 17.3, J_2 = 1.5, 1H), 6.19 (dd, J_1 = 17.3, J_2 = 10.4, 1H), 5.86 (dd, J_1 = 10.4, J_2 = 1.5, 1H), 5.22 (s, 2H); ^{13}C-NMR (75.47 MHz, $CDCl_3$): 166.1, 135.9, 131.2, 128.7, 128.4, 128.34, 128.32, 66.4; IR (film) ṽ = 3067, 3035, 2954, 2893, 1720, 1634, 1619, 1498, 1455, 1406, 1372, 1296, 1267, 1172, 1048, 982, 966, 808, 735, 695 cm^{-1}; HRMS: no molecular ion peak could be detected due to the poor ionisation of compound **SI01**.

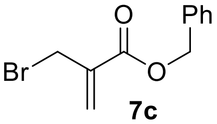

7c

Benzyl 2-(bromomethyl)acrylate (7c).

7c was prepared according to a literature procedure in 77% overall yield over two steps from benzyl acrylate (**SI01**) on a 1.5 g scale (referring to the final product).[1]

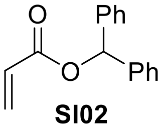

SI02

Benzhydryl acrylate (SI02).

Acrylic acid (1.13 g, 15.7 mmol, 1 eq.) was dissolved in DMF (15 mL) and cooled to 0 °C. K_2CO_3 (2.17 g, 15.7 mmol, 1 eq.) was added over a period of 5 min, the ice bath was removed and a solution of bromodiphenylmethane (3.88 g, 15.7 mmol, 1 eq.) in DMF (5 mL) was added slowly. The mixture was heated to 80 °C (oil bath temperature) and kept at that temperature for 16 h. The obtained slurry was cooled to room temperature and the reaction was quenched by the addition of water (ca. 20 mL). The mixture was extracted with EtOAc (3 x 40 mL) and the combined organic phase was washed with saturated aqueous NH_4Cl solution, dried over Na_2SO_4, filtered and concentrated. The remaining crude product was purified via flash chromatography (silica gel, hexanes/EtOAc 15/1) to give the product including an inseparable impurity of the bromodiphenylmethane as a clear oil. This mixture was used for the subsequent synthesis of **SI03** without further purification.

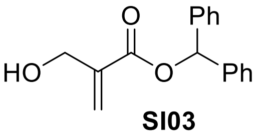

SI03

Benzhydryl 2-(hydroxymethyl)acrylate (SI03).

The mixture of **SI02** and the impurity from the previous experiment (*vide supra*) was dissolved in dioxane/water 1/1 (100 mL). Formaldehyde (5.7 mL, 30% aqueous solution, 157 mmol, 10 eq.) and 1,4-diazabicyclo[2.2.2]octane (DABCO, 1.76 g, 15.7 mmol, 1 eq.) were added and the mixture was stirred at room temperature for 28 h. The reaction was quenched by the addition of water (ca. 20 mL), extracted with EtOAc (3 x 40 mL) and the combined organic phase was dried over Na_2SO_4, filtered and concentrated. The obtained crude product was purified via flash chromatography (silica gel, hexanes/EtOAc 4/1) to give **SI03** as a clear oil (2.13 g, 7.90 mmol, 50% over two steps).

^{1}H-NMR (300.13 MHz, $CDCl_3$): 7.45 – 7.19 (m, 10H), 6.96 (s, 1H), 6.41 (s, 1H), 5.90 (s, 1H), 4.37 (d, J = 5.7 Hz, 2H), 2.31 (br s, 1H); ^{13}C-NMR (75.47 MHz, $CDCl_3$): 165.4, 140.0, 139.6, 128.7, 128.2, 127.1, 126.4, 77.5, 62.6; IR (film) ṽ = 3385 (br), 3085, 3059, 3027, 1713, 1656, 1597, 1493, 1446,

1270, 1172, 1154, 1031, 1017, 851, 752, 734, 695, 651, 600, 540 cm^{-1}; HRMS(APCI-negative): *m/z*: calc. for $C_{17}H_{15}O_3^-$: 267.1027 [M-H]$^-$, found: 267.1029.

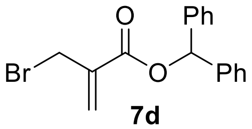
7d

Benzhydryl 2-(bromomethyl)acrylate (7d).

Alcohol **SI04** (1.89 g, 7.00 mmol, 1 eq.) was dissolved in diethyl ether (10 mL) and the mixture was cooled to 0 °C. PBr_3 (333 µL, 949 mg, 3.50 mmol, 0.5 eq.) was added slowly via syringe and cannula and the mixture was stirred for 10 min at 0 °C and 3 h at room temperature. The reaction was cooled to 0 °C and quenched by the addition of cold water (10 mL). The obtained solution was extracted with EtOAc (3 x 10 mL), the combined organic phase was dried over Na_2SO_4, filtered and concentrated. The obtained crude product was via flash chromatography (silica gel, hexanes/EtOAc 10/1) to give the allylic bromide **7d** as a clear oil (1.88 g, 5.70 mmol, 81%).

^{1}H-NMR (300.13 MHz, $CDCl_3$): 7.43-7.28 (m, 10H), 6.99 (s, 1H), 6.48 (d, *J* = 0.6, 1H), 6.02 (d, *J* = 0.7, 1H), 4.24 (d, *J* = 0.8, 2H); ^{13}C-NMR (75.47 MHz, $CDCl_3$): 164.0, 140.0, 137.5, 130.0, 128.7, 128.2, 127.2, 78.1, 29.3; IR (film) ṽ = 3088, 3063, 3031, 1720, 1495, 1453, 1398, 1323, 1301, 1220, 1168, 1112, 914, 807, 757, 742, 694, 627, 567 cm^{-1}; HRMS: no molecular ion peak could be detected due to the poor ionisation of compound **7d**.

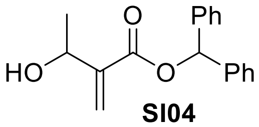
SI04

Benzhydryl 3-hydroxy-2-methylenebutanoate (SI04).

The crude acrylate **SI02** (1.50 g, 6.29 mmol, 1 eq.) was dissolved in dioxane/water 1/1 (42 mL). Acetaldehyde (1.06 mL, 833 mg, 18.9 mmol, 3 eq.) and 1,4-diazabicyclo[2.2.2]octane (DABCO, 705 mg, 6.29 mmol, 1 eq.) were added and the mixture was stirred at room temperature for 32 h. The reaction was quenched by the addition of water (ca. 15 mL), extracted with EtOAc (3 x 40 mL) and the combined organic phase was dried over Na_2SO_4, filtered and concentrated. The obtained crude product was purified via flash chromatography (silica gel, hexanes/EtOAc 5/1) to give the product as colorless oil (994 mg, 3.52 mmol, 56%).

^{1}H-NMR (300.13 MHz, $CDCl_3$): 7.38-7.28 (m, 10H), 6.97 (s, 1H), 6.40 (s, 1H), 5.91 (t, *J* = 1.0, 1H), 4.67 (q, *J* = 6.4, 1H), 2.61 (br s, 1H), 1.39 (d, *J* = 6.5, 3H); ^{13}C-NMR (75.47 MHz, $CDCl_3$): 165.7, 143.8, 140.1, 128.7, 128.2, 127.1, 124.8, 77.6 (overlapping with $CDCl_3$ signal), 67.2, 22.1; IR (film) ṽ = 3431 (br), 3089, 3064, 3032, 2975, 2928, 1710, 1629, 1495, 1452, 1257, 1155, 1079, 958, 913, 816, 757, 742, 694, 601, 579 cm^{-1}; HRMS(ESI): *m/z*: calc. for $C_{18}H_{18}O_3Na^+$: 305.1148 [M+Na]$^+$, found: 305.1148.

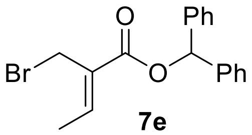
7e

Benzhydryl (*Z*)-2-(bromomethyl)but-2-enoate (7e).

Alcohol **SI04** (617 mg, 2.19 mmol, 1 eq.) was dissolved in diethyl ether (3.5 mL) and the mixture was cooled to 0 °C. PBr_3 (104 µL, 296 mg, 1.09 mmol, 0.5 eq.) was added slowly via syringe and canula and the mixture was stirred for 10 min at 0 °C and 3 h at room temperature. The reaction was cooled to 0 °C and quenched by the addition of cold water (5 mL). The obtained mixture was extracted with EtOAc (3 x 10 mL), the combined organic phase was dried over Na_2SO_4, filtered and concentrated. The obtained crude product was via flash chromatography (silica gel, hexanes/EtOAc 10/1) to give the allylic bromide **7e** as a clear oil (391 mg, 1.13 mmol, 52%). The (*Z*)-geometry was confirmed via the correlations in a ^{1}H,^{1}H-NOESY spectrum (see NMR spectra for details).

^{1}H-NMR (300.13 MHz, $CDCl_3$): 7.33-7.18 (m, 10H), 7.13 (q, *J* = 7.3, 1H), 6.89 (s, 1H), 4.21 (s, 2H), 1.87 (d, *J* = 7.3, 3H); ^{13}C-NMR (75.47 MHz, $CDCl_3$): 164.6, 144.2, 140.3, 130.5, 128.7, 128.1, 127.2, 77.8, 24.1, 14.8; IR (film) ṽ = 3088, 3063, 3031, 2939, 2850, 1715, 1643, 1495, 1452, 1382, 1353, 1262, 1216, 1159, 1124, 1045, 1030, 1002, 966, 759, 742, 696, 666, 597, 577 cm^{-1}; HRMS: no molecular ion peak could be detected due to the poor ionisation of compound **7e**.

Synthesis of substitued acrylamides.

Acrylic acid (1.18 g, 16.5 mmol, 1 eq.) was dissolved in DCM (50 mL) and DMF was added (1.22 mL, 16.5 mmol, 1 eq.) and cooled to 0 °C. A 2 M solution of $(COCl)_2$ in DCM (8.25 mL, 16.5 mmol, 1 eq.) was added over a period of 5 min, the ice bath was removed and the reaction stirred at room temperature for 3 hours. Separately, a solution of diethylamine (853 µL, 8.25 mmol, 1 eq.) or dibenzylamine (1.59 mL, 8.25 mmol, 1 eq.) was prepared in 25 mL of DCM. Half of the acryloyl chloride solution was added slowly in the amine solution and the reaction stirred at room temperature overnight. The mixture was diluted with DCM (25 mL) and washed with saturated aqueous $NaHCO_3$ solution (10 mL) and brine (10 mL). The organic phase was dried over Na_2SO_4, filtered and concentrated. The remaining crude product was purified via flash chromatography.

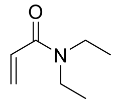

SI5

N,*N*-diethylacrylamide (SI5).

Flash chromatography (silica gel, from cyclohexane/EtOAc 3/1 to 2/1) gave the product including an inseparable impurity of the Micheal addition product as a clear oil. This mixture was used for the subsequent synthesis of **SI7** without further purification.

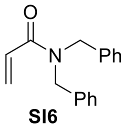

SI6

N,*N*-dibenzylacrylamide (SI6).

Flash chromatography (silica gel, from cyclohexane/EtOAc 6/1 to 3/1) gave the product including an inseparable impurity of the Micheal addition product as a clear yellowish oil. This mixture was used for the subsequent synthesis of **SI8** without further purification.

Baylis-Hillman reaction of substitued acrylamides.

The mixture of **SI5** or **SI6** and the impurity from the previous experiment (*vide supra*) was dissolved in *tert*-Butyl alcohol (5 mL) in a biotage vial. Formaldehyde (5.7 mL, 37% aqueous solution, 70 mmol, 10 eq.) and 1,4-diazabicyclo[2.2.2]octane (DABCO, 794 mg, 7 mmol, 1 eq.) and phenol (165 mg, 1.75 mmol, 0.25 eq.) were added and the vial was sealed. The mixture was heated up to 80 °C and stirred for 3 days (in case of the *N*,*N*-dibenzylacrylamide) or 7 days (in case of the *N*,*N*-diethylacrylamide). The reaction was cooled down and the *tert*-Butyl alcohol was evaporated under vacuum. The resulting crude was diluted with water (ca. 7 mL), extracted with EtOAc (3 x 30 mL) and the combined organic phase was dried over Na_2SO_4, filtered and concentrated. The obtained crude product was purified via flash chromatography.

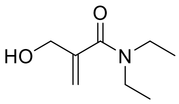

SI7

N,*N*-diethyl-2-(hydroxymethyl)acrylamide (SI7).

Flash chromatography (silica gel, from cyclohexane/EtOAc 1/1 to EtOAc) gave the product including an inseparable impurity as a clear oil (150 mg, 0.96 mmol, 11% over three steps). The compound was used for the subsequent synthesis of **SI9** without further purification.

^{1}H NMR (300 MHz, Chloroform-*d*) δ 5.45 – 5.34 (m, 1H), 5.16 (m, 1H), 4.27 (dt, J_1 = 7.9, J_2 = 1.3 Hz, 2H), 3.80 (br s, 1H), 3.47 – 3.20 (m, 4H), 1.20 – 1.04 (m, 6H). ^{13}C NMR (75 MHz, $CDCl_3$) δ 170.9, 144.7, 113.7, 63.8, 41.9, 40.1, 14.1, 13.0. IR (film) ῦ = 3373 (br), 2973, 2936, 1601, 1480, 1458, 1434, 1380, 1363, 1316, 1291, 1250, 1218, 1132, 1098, 1061, 1039, 921, 791, 752, 732, 627, 567 cm^{-1}; HRMS(ESI): *m*/*z*: calc. for $C_8H_{16}NO_2^+$: 158.11785 $[M+H]^+$, found: 158.117555.

N,*N*-dibenzyl-2-(hydroxymethyl)acrylamide (SI8).

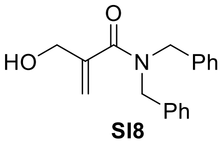

SI8

Flash chromatography (silica gel, from cyclohexane/EtOAc 3/1 to 1/1) gave the as a clear colorless oil (929 mg, 3.3 mmol, 40% over three steps).

^{1}H NMR (300 MHz, Chloroform-*d*) δ 7.50 – 7.10 (m, 10H), 5.46 (s, 1H), 5.30 (s, 1H), 4.59 (s, 4H), 4.39 (s, 2H), 3.10 (br s, 1H).^{13}C NMR (75 MHz, $CDCl_3$) δ 171.8, 143.7, 136.5, 129.2, 128.9, 128.3, 127.7, 127.1, 115.4, 64.4, 51.3, 46.8. IR (film) ṽ = 3372, 3029, 2923, 1647, 1601, 1495, 1468, 1450, 1426, 1362, 1311, 1266, 1223, 1203, 1179, 1068, 1028, 919, 730, 696, 609, 555, 488, 456 cm^{-1}; HRMS(ESI): *m*/*z*: calc. for $C_{18}H_{20}NO_2^+$: 282.148855 [M+H]$^+$, found: 282.14908.

N,*N*-diethyl-2-(bromomethyl)acrylamide (SI9).

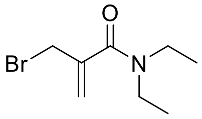

SI9

Alcohol **SI7** (100 mg, 0.64 mmol, 1 eq.) was dissolved in diethyl ether (5 mL) and the mixture was cooled to 0 °C. PBr_3 (45 µL, 129 mg, 0.48 mmol, 0.5 eq.) was added slowly via syringe and cannula and the mixture was stirred for 10 min at 0 °C and 3 h at room temperature. The reaction was cooled to 0 °C and quenched by the addition of cold water (5 mL). The obtained solution was extracted with EtOAc (3 x 30 mL), the combined organic phase was dried over Na_2SO_4, filtered and concentrated. The obtained crude product was via flash chromatography (silica gel, cyclohexane /EtOAc 3/1 to 2/1) to give the allylic bromide **SI9** as a colourless oil (31 mg, 0.14 mmol, 22%).

^{1}H NMR (300 MHz, Chloroform-*d*) δ 5.45 (s, 1H), 5.18 (s, 1H), 4.22 (d, *J* = 1.0 Hz, 2H), 3.47 (dd, J_1 = 12.1, J_2 = 6.6 Hz, 4H), 1.18 (t, *J* = 7.1 Hz, 6H). ^{13}C NMR (75 MHz, $CDCl_3$) δ 169.0, 140.8, 116.8, 43.0, 39.1, 33.6, 14.5, 12.6; IR (film) ṽ = 2972, 2935, 2875, 1639, 1612, 1475, 1459, 1433, 1381, 1318, 1242, 1213, 1142, 1104, 930, 777, 734, 629, 572, 519, 478 cm^{-1}; HRMS(ESI): *m*/*z*: calc. for $C_8H_{15}BrNO^+$: 220.033136 [M+H]$^+$, found: 220.033153.

N,*N*-dibenzyl-2-(bromomethyl)acrylamide (7f).

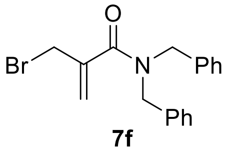

7f

Alcohol **SI8** (929 mg, 3.30 mmol, 1 eq.) was dissolved in diethyl ether (6 mL) and the mixture was cooled to 0 °C. PBr_3 (155 µL, 447 mg, 1.65 mmol, 0.5 eq.) was added slowly via syringe and cannula and the mixture was stirred for 10 min at 0 °C and 3 h at room temperature. The reaction was cooled to 0 °C and quenched by the addition of cold water (5 mL). The obtained solution was extracted with EtOAc (3 x 30 mL), the combined organic phase was dried over Na_2SO_4, filtered and concentrated. The obtained crude product was via flash chromatography (silica gel, cyclohexane /EtOAc 3/1) to give the allylic bromide **7f** as a yellowish solid (291 mg, 0.85 mmol, 26%).

Mp: 59-61 °C (from $CDCl_3$); ^{1}H NMR (300 MHz, Chloroform-*d*) δ 7.55 – 7.15 (m, 10H), 5.49 (d, *J* = 1.0 Hz, 1H), 5.31 (s, 1H), 4.82 – 4.50 (m, 4H), 4.36 (d, *J* = 0.9 Hz, 2H). ^{13}C NMR (75 MHz, $CDCl_3$) δ 170.1, 139.9, 136.4, 129.0, 128.7, 128.3, 127.7, 127.6, 126.9, 117.7, 51.2, 47.1, 33.8. IR (film) ṽ = 3059, 3027, 2955, 2927, 1645, 1619, 1603, 1495, 1473, 1450, 1433, 1420, 1398, 1363, 1353, 1315, 1210, 1169, 1026, 965, 963, 900, 741, 724, 693, 650, 569, 520, 458 cm^{-1}; HRMS(ESI): *m*/*z*: calc. for $C1_8H_{19}BrNO^+$: 344.063994 [M+H]$^+$, found: 344.064454.

General procedure for the asymmetric allylation of ketones (Procedure A).

A 5 mL screw cap vial with magnetic stirring bar was charged with zinc (33.0 mg, 500 µmol, 5 eq.), ammonium chloride (43.0 mg, 800 µmol, 8 eq.) and (*S*)-3,3′-bis(2,4,6-triisopropylphenyl)-1,1′-binaphthyl-2,2′-diyl hydrogenphosphate (TRIP, 7.5 mg, 10.0 µmol, 0.1 eq.) followed by the respective solvent mixture (see compounds below for details), the ketone (100 µmol) and the corresponding allyl bromide (150 µmol, 1.5 eq.). The mixture was stirred (720 rpm) at room temperature for 16 h and was quenched consecutively by the addition of saturated aqueous NH_4Cl solution (5 mL). The mixture was extracted with EtOAc (3 x 10 mL) and the combined organic phase was dried over Na_2SO_4, filtered and concentrated under reduced pressure. The obtained crude product was purified via flash chromatography (silica gel, the respective eluents are indicated below) to give the pure product.

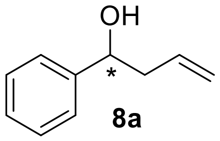

8a

1-Phenylbut-3-en-1-ol (8a).

Procedure A: Benzaldehyde, allyl bromide **7a**; solvent: toluene (1.0 mL); flash chromatography (silica gel, hexanes/EtOAc 5/1), yield: 14.0 mg, 95%, colorless oil; ^{1}H-NMR (300.13 MHz, $CDCl_3$): 7.37-7.24 (m, 5H), 5.87-5.73 (m, 1H), 5.19-5.11 (m, 2H), 4.72 (dd, J_1 = 7.3, J_2 = 5.7, 1H), 2.53-2.47 (m, 2H), 2.14 (br s, 1H); ^{13}C-NMR (75.47 MHz, $CDCl_3$): 144.0, 134.6, 128.5, 127.7, 125.9, 118.5, 73.4, 43.9; IR (film) ṽ = 3375 (br), 1492, 1453, 1197, 1077, 1043, 1029, 913, 756, 698, 641, 608, 537 cm^{-1}; HPLC analysis on chiral stationary phase {Daicel Chiralcel OD-H, *n*-heptane/2-propanol 99/1, 0.8 mL/min, 25 °C, UV 215 nm, t_{ret}(enantiomer 1) = 19.7 min, t_{ret}(enantiomer 2) = 23.0 min}: only racemic material was obtained; analytical data is accordance with literature.[2]

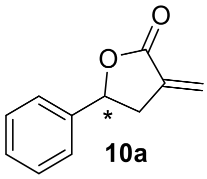

10a

3-Methylene-5-phenyldihydrofuran-2(3*H*)-one (10a).

Procedure A: Benzaldehyde, allyl bromide **7b**; solvent: toluene (1.0 mL); flash chromatography (silica gel, hexanes/EtOAc 5/1); yield: 14.6 mg, 84%, colorless oil; $[\alpha]_D^{20}$ = +4.4 (c = 1.9, $CHCl_3$); lit.: $[\alpha]_D^{23}$ = +12.7 (c = 1.0, $CHCl_3$, (*S*)-enantiomer (77% ee);[3] $[\alpha]_D^{20}$ = -2.0 (c = 1.9, CH_2Cl_2); lit.: $[\alpha]_D^{25}$ = -15.7 [c = 1.3, CH_2Cl_2, (*S*)-enantiomer (56% ee];[4] ^{1}H-NMR (300.13 MHz, $CDCl_3$): 7.46 – 7.28 (m, 5H), 6.31 (t, *J* = 2.8 Hz, 1H), 5.69 (t, *J* = 2.5 Hz, 1H), 5.53 (dd, J_1 = 7.9, J_2 = 6.6 Hz, 1H), 3.41 (ddt, J_1 = 17.1, J_2 = 8.1, J_3 = 2.5 Hz, 1H), 2.91 (ddt, J_1 = 17.1, J_2 = 6.4, J_3 = 2.9 Hz, 1H); ^{13}C-NMR (75.47 MHz, $CDCl_3$): 170.3, 139.9, 134.3, 129.0, 128.7, 125.5, 122.6, 78.1, 36.4; IR (film) ṽ = 3093, 3066, 3050, 3037, 2974, 2919, 2853, 1752, 1602, 1551, 1496, 1459, 1437, 1402, 1375, 1319, 1277, 1240, 1215, 1126, 1080, 1020, 985, 962, 938, 818, 761, 701, 639, 562 cm^{-1}; HPLC analysis on chiral stationary phase {Daicel Chiralcel IC, *n*-heptane/2-propanol 85/15, 1.0 mL/min, 20 °C, UV 215 nm, t_{ret}(enantiomer 1) = 10.1 min, t_{ret}(enantiomer 2) = 13.9 min}: t_{ret}(major isomer) = 13.7 min, 50% ee; HRMS(ESI): *m/z*: calc. for $C_{11}H_{11}O_2^+$: 175.0754 $[M+H]^+$, found: 175.0755.

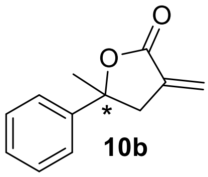

10b

5-Methyl-3-methylene-5-phenyldihydrofuran-2(3*H*)-one (10b).

Procedure A: Acetophenone, allyl bromide **7b**; solvent: toluene (1.0 mL); flash chromatography (silica gel, hexanes/EtOAc 3/1); yield: 16.9 mg, 90%, colorless oil; ^{1}H-NMR (300.13 MHz, $CDCl_3$): 7.42 – 7.27 (m, 5H), 6.26 (t, *J* = 2.8 Hz, 1H), 5.64 (t, *J* = 2.4 Hz, 1H), 3.19 – 3.13 (m, 2H), 1.73 (s, 3H); ^{13}C-NMR (75.47 MHz, $CDCl_3$): 169.8, 144.7, 135.2, 128.8, 127.8, 124.3, 122.8, 84.1, 42.8, 30.2; IR (film) ṽ = 2957, 2921, 2851, 1760, 1690, 1653, 1601, 1496, 1446, 1378, 1238, 1052, 1027, 947, 763, 697 cm^{-1}; HPLC analysis on chiral stationary phase {Daicel Chiralcel OJ, *n*-heptane/2-propanol 85/15, 0.7 mL/min, 20 °C, UV 215 nm, t_{ret}(enantiomer 1) = 18.4 min, t_{ret}(enantiomer 2) = 21.8 min}: t_{ret}(major isomer) = 17.5 min, 70% ee; HRMS(ESI): *m/z*: calc. for $C_{12}H_{13}O_2^+$: 189.0910 $[M+H]^+$, found: 189.0911.

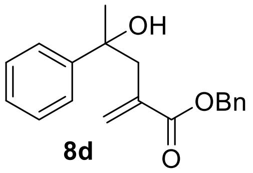

8d

Benzyl 4-hydroxy-2-methylene-4-phenylpentanoate (8d).

Procedure A: Acetophenone, allyl bromide **7c**; solvent: toluene (1.0 mL); compound **8d** was prepared on analytical scale only. The sample was quenched in saturated aqueous NH_4Cl solution (500 µL), extracted with CH_2Cl_2 (3 x 5 mL) and the combined organic phase was dried over Na_2SO_4, filtered and the filtrate was treated with trifluoroacetic acid (1.1 mg, 0.8 µL, 10 µmol, 0.1 eq.) and stirred at room temperature for 16 h to yield lactone **10b**. The reaction was quenched by the addition of saturated aqueous $NaHCO_3$ solution (5 mL), the organic phase was separated, dried over Na_2SO_4, filtered and subjected to HPLC analysis on chiral stationary phase {Daicel Chiralcel OJ, *n*-heptane/2-propanol 85/15, 0.7 mL/min, 20 °C, UV 215 nm, t_{ret}(enantiomer 1) = 18.4 min, t_{ret}(enantiomer 2) = 21.8 min}: t_{ret}(major isomer) = 17.5 min, 77% ee.

General procedure for the preparation of racemic reference material (Procedure B).

A HPLC vial with magnetic stirring bar was charged with the ketone (25.0 µmol, 1 eq.), zinc (8.0 mg, 125 µmol, 5 eq.), NH_4Cl (11.0 mg, 200 µmol, 8 eq.) and diphenyl phosphate (2.0 mg, 8.0 µmol, 0.3 eq.). Allylic bromide **7d** or **7e** (36.0 µmol, 1.5 eq.) dissolved in toluene (200 µL) was added and the reaction mixture was stirred at room temperature for 16 h. The suspension was filtered through a plug of silica gel (~1 g), the plug was rinsed with additional EtOAc (ca. 1 mL) and the combined filtrates were concentrated. The residue was dissolved in a small amount of EtOAc (ca. 100 µL) and half of the solution was adsorbed on the starting line of a silica gel TLC plate (~8 cm wide). The plate was developed in the solvent indicated for flash chromatography for the specific compound (indicated below) and the product band was scratched off. The obtained silica gel with the adsorbed product was transferred into a HPLC vial with magnetic stirring bar and was extracted by stirring with 2-propanol (800 µL) for 30 min at room temperature. The suspension was filtered through a syringe filter (Nylon, 0.2 µm) and subjected to HPLC-MS on an achiral stationary phase and HPLC-UV analysis on a chiral stationary phase.

General procedure for the asymmetric allylation of ketones (Procedure C).

A 5 mL screw cap vial was charged with zinc (33.0 mg, 500 µmol, 5 eq.), NH_4Cl (43.0 mg, 800 µmol, 8 eq.) and (*S*)-3,3'-bis(2,4,6-triisopropylphenyl)-1,1'-binaphthyl-2,2'-diyl hydrogenphosphate (TRIP, 7.5 mg, 10.0 µmol, 0.1 eq.) followed by the respective solvent mixture (see compounds below for details).The ketone (100 µmol) and the allyl bromide **7d** (50.0 mg, 150 µmol, 1.5 eq.) were added [in case of product **8q** benzhydryl (*Z*)-2-(bromomethyl)but-2-enoate (**7e**, 52.0 mg, 150 µmol, 1.5 eq.). The mixture was stirred (720 rpm) at room temperature for 16 h, quenched by the addition of $NH_4Cl_{sat., aq.}$ solution (5 mL) and extracted with EtOAc (3 x 10 mL). The combined organic phase was dried over Na_2SO_4, filtered and concentrated under reduced pressure. The obtained crude product was purified via flash chromatography (SiO_2, eluent is indicated below) to give the pure product.

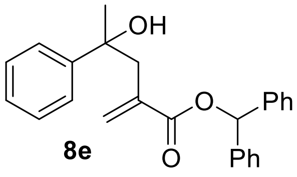

8e

Benzhydryl 4-hydroxy-2-methylene-4-phenylpentanoate (8e).

Procedure C: Acetophenone (12.0 mg, 100 µmol, 1 eq.); solvent: toluene/cyclohexane 1/1 (2 mL); flash chromatography (silica gel, hexanes/EtOAc 5/1); yield: 33.0 mg, 89.0 µmol, 89%, colorless oil; $[\alpha]_D^{20}$ = +21.4 (c = 0.28, $CHCl_3$); ^{1}H-NMR (300.13 MHz, $CDCl_3$): 7.44-7.19 (m, 15H), 6.91 (s, 1H), 6.36 (d, *J* = 1.3, 1H), 5.51 (d, *J* =1.0, 1H), 3.39 (br s, 1H), 2.93 (dd, J_1 = 14.0, J_2 = 0.6, 1H), 1.55 (s, 3H); ^{13}C-NMR (75.47 MHz, $CDCl_3$): 168.0, 147.5, 140.04, 140.01, 136.6, 129.9, 128.69, 128.67, 128.15, 128.12, 127.2, 127.1, 126.7, 126.6, 125.1, 78.0, 74.2, 46.5, 29.9; IR (film) ũ = 3462 (br), 3062, 3030, 2974, 2930, 1698, 1624, 1494, 1447, 1300, 1150, 953, 910, 863, 759, 741, 695, 590 cm^{-1}; HPLC analysis on chiral stationary phase {Daicel Chiralpak IA, *n*-heptane/2-propanol 90/10, 1.0 mL/min, 20 °C, UV 230 nm, t_{ret}(enantiomer 1) = 10.1 min,

t_{ret}(enantiomer 2) = 12.0 min}: t_{ret}(major isomer) = 10.1 min, 90% ee; HRMS(ESI): *m/z*: calc. for $C_{25}H_{24}O_3Na^+$: 395.1617 [M+Na]$^+$, found: 395.1618.

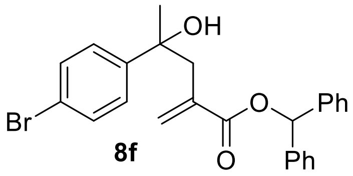

8f

Benzhydryl 4-(4-bromophenyl)-4-hydroxy-2-methylenepentanoate (8f).

Procedure C: 4'-Bromoacetophenone (20.0 mg, 100 µmol, 1 eq.); solvent: toluene/cyclohexane 2/1 (1.5 mL); flash chromatography (silica gel, hexanes/EtOAc 5/1); yield: 41.0 mg, 88.0 µmol, 88%, colorless oil; $[\alpha]_D^{20}$ = +26.9 (c = 1.0, $CHCl_3$); ^{1}H-NMR (300.13 MHz, $CDCl_3$): 7.40-7.22 (m, 14H), 6.87 (s, 1H), 6.34 (d, *J* = 1.2, 1H), 5.48 (d, *J* = 0.8, 1H), 3.59 (br s, 1H), 2.89 (dd, J_1 = 14.0, J_2 = 0.6, 1H), 2.78 (d, *J* = 14.1, 1H), 1.51 (s, 3H); ^{13}C-NMR (75.47 MHz, $CDCl_3$): 168.0, 146.5, 139.91, 139.88, 136.4, 131.1, 130.2, 128.72, 128.70, 128.22, 128.17, 127.2, 127.1, 127.0, 120.5, 78.1, 74.0, 46.5, 30.1; IR (film) ῦ = 3445 (br), 3063, 3031, 2975, 2930, 1698, 1624, 1487, 1453, 1341, 1276, 1151, 1077, 1008, 911, 826, 742, 696, 589 cm^{-1}; HPLC analysis on chiral stationary phase {Daicel Chiralpak IA, *n*-heptane/2-propanol 90/10, 1.0 mL/min, 20 °C, UV 230 nm, t_{ret}(enantiomer 1) = 11.0 min, t_{ret}(enantiomer 2) = 13.9 min}: t_{ret}(major isomer) = 11.0 min, 87% ee; HRMS(ESI): *m/z*: calc. for $C_{25}H_{23}BrO_3Na^+$: 473.0723 and 475.0706 [M+Na]$^+$, found: 473.0722 and 475.0709 (for the two most prominent isotopes).

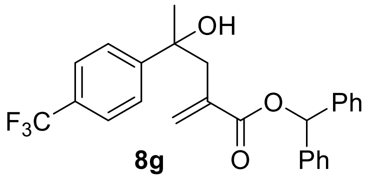

8g

Benzhydryl 4-hydroxy-2-methylene-4-[4-(trifluoromethyl)phenyl]pentanoate (8g).

Procedure C: 4'-(Trifluoromethyl)-acetophenone (16.0 mg, 100 µmol, 1 eq.); solvent: toluene/cyclohexane 2/1 (1.5 mL); flash chromatography (silica gel, hexanes/EtOAc 5/1); yield: 39.0 mg, 89.0 µmol, 89%, colorless oil; $[\alpha]_D^{20}$ = +24.7 (c = 1.0, $CHCl_3$); ^{1}H-NMR (300.13 MHz, $CDCl_3$): 7.69 (br s, 1H), 7.57 (d, *J* =7.7, 1H), 7.47 (d, *J* = 7.7, 1H), 7.41-7.29 (m, 11H), 6.90 (s, 1H), 6.35 (d, *J* = 1.2, 1H), 5.48 (d, *J* = 0.9, 1H), 3.72 (br s, 1H), 2.93 (dd, J_1 = 14.1, J_2 = 0.6, 1H), 2.80 (d, *J* = 14.1, 1H), 1.54 (s, 3H); ^{13}C NMR (75.47 MHz, $CDCl_3$) δ 168.2, 148.6, 139.87, 139.84, 136.2, 130.42 (C–F, $^2J_{C-F}$ = 31.9 Hz), 130.37, 128.7 (C–F, $^4J_{C-F}$ = 1.9 Hz), 128.6, 128.3, 127.2, 127.0, 124.4 (C–F, $^1J_{C-F}$ = 272.8 Hz), 123.5 (C–F, $^3J_{C-F}$ = 3.9 Hz), 122.0 (C–F, $^3J_{C-F}$ = 3.9 Hz). 78.2, 74.1, 46.6, 30.0; ^{19}F-NMR (282.39 MHz, $CDCl_3$): -62.43 (s); IR (film) ῦ = 3449 (br), 3065, 3033, 2976, 2930, 1697, 1625, 1495, 1454, 1327, 1161, 1119, 1071, 956, 804, 755, 743, 696, 654, 588 cm^{-1}; HPLC analysis on chiral stationary phase {Daicel Chiralpak IA, *n*-heptane/2-propanol 90/10, 1.0 mL/min, 20 °C, UV 230 nm, t_{ret}(enantiomer 1) = 10.1 min, t_{ret}(enantiomer 2) = 12.0 min}: t_{ret}(major isomer) = 10.1 min, 88% ee; HRMS(ESI): *m/z*: calc. for $C_{26}H_{23}F_3O_3Na^+$: 463.1492 [M+Na]$^+$, found: 463.1495.

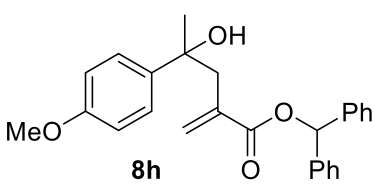

8h

Benzhydryl 4-hydroxy-4-(4-methoxyphenyl)-2-methylenepentanoate (8h).

Procedure C: 4'-Methoxyacetophenone (15.0 mg, 100 µmol, 1 eq.); solvent: toluene/cyclohexane 2/1 (1.5 mL); flash chromatography (silica gel, hexanes/EtOAc 10/1); yield: 34.0 mg, 85.0 µmol, 85%, colorless oil; $[\alpha]_D^{20}$ = +26.7 (c = 1.0, $CHCl_3$); ^{1}H-NMR (300.13 MHz, $CDCl_3$): 7.38-7.27 (m, 12H), 6.88 (s, 1H), 6.84-6.79 (m, 2H), 6.33 (d, *J* = 1.3, 1H), 5.48 (d, *J* = 1.0, 1H), 3.78 (s, 3H), 3.28 (br s, 1H), 2.89 (d, *J* = 14.4, 1H), 2.81 (d, *J* = 13.9, 1H), 1.51 (s, 3H); ^{13}C-NMR (75.47 MHz, $CDCl_3$): 168.0, 158.2, 140.1, 140.0, 139.7, 136.7, 129.8, 128.67, 128.66, 128.13, 128.10, 127.2, 127.1, 126.2, 113.4, 77.9, 73.9, 55.3, 46.6, 30.0; IR (film) ῦ = 3466 (br), 3032, 2971, 2932, 2835, 1713, 1611, 1510, 1454, 1299, 1246, 1151, 1031, 954, 908, 864, 811, 731, 696 cm^{-1}; HPLC analysis on chiral stationary phase {Daicel Chiralpak IA, *n*-heptane/2-propanol 90/10, 1.0 mL/min, 20 °C, UV 215 nm, t_{ret}(enantiomer 1) = 13.7 min, t_{ret}(enantiomer 2) = 18.5 min}:

t_{ret}(major isomer) = 14.3 min, 87% ee; HRMS(ESI): *m/z*: calc. for $C_{26}H_{26}O_4Na^+$: 425.1723 $[M+Na]^+$, found: 425.1722.

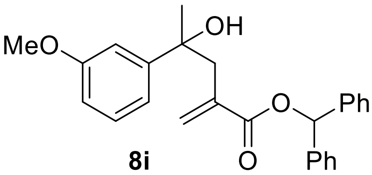

8i

Benzhydryl 4-hydroxy-4-(3-methoxyphenyl)-2-methylenepentanoate (8i).

Procedure C: 3'-Methoxyacetophenone (15.0 mg, 100 µmol, 1 eq.); solvent: toluene/cyclohexane 2/1 (1.5 mL); flash chromatography (silica gel, hexanes/EtOAc 5/1); yield: 33.0 mg, 82.0 µmol, 82%, colorless oil; $[\alpha]_D^{20}$ = +15.6 (c = 1.0, $CHCl_3$); ^{1}H-NMR (300.13 MHz, $CDCl_3$): 7.46 – 7.27 (m, 10H), 7.21 (t, *J* = 7.9 Hz, 1H), 7.03 – 6.98 (m, 1H), 6.95 (ddd, J_1 = 7.7, J_2 = 1.6, J_3 = 0.9 Hz, 1H), 6.90 (s, 1H), 6.75 (ddd, J_1 = 8.2, J_2 = 2.6, J_3 = 0.8 Hz, 1H), 6.34 (d, *J* = 1.3 Hz, 1H), 5.51 (d, *J* = 1.0 Hz, 1H), 3.77 (s, 3H), 2.90 (dd, J_1 = 14.0, J_2 = 0.6, 1H), 2.82 (d, *J* = 14.0, 1H), 1.51 (s, 3H); ^{13}C-NMR (75.47 MHz, $CDCl_3$): 168.1, 159.6, 149.4, 140.03, 140.00, 136.6, 130.0, 129.1, 128.69, 128.68, 128.2, 128.1, 127.2, 127.1, 117.5, 112.0, 111.0, 78.0, 74.2, 55.3, 46.4, 29.9; IR (film) ῦ = 3467 (br), 3063, 3031, 2961, 2852, 1713, 1600, 1583, 1487, 1453, 1432, 1288, 1255, 1241, 1153, 10.43, 955, 758, 741, 695 cm^{-1}; HPLC analysis on chiral stationary phase {Daicel Chiralpak IA, *n*-heptane/2-propanol 90/10, 1.0 mL/min, 20 °C, UV 230 nm, t_{ret}(enantiomer 1) = 9.9 min, t_{ret}(enantiomer 2) = 17.1 min}: t_{ret}(major isomer) = 9.8 min, 82% ee; HRMS(ESI): *m/z*: calc. for $C_{26}H_{26}O_4Na^+$: 425.1723 $[M+Na]^+$, found: 425.1722.

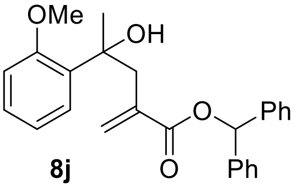

8j

Benzhydryl 4-hydroxy-4-(2-methoxyphenyl)-2-methylenepentanoate (8j).

Procedure C: 2'-Methoxyacetophenone (15.0 mg, 100 µmol, 1 eq.); solvent: toluene/cyclohexane 2/1 (1.5 mL); flash chromatography (silica gel, hexanes/EtOAc 10/1); yield: 29.0 mg, 72.0 µmol, 72%, colorless oil; $[\alpha]_D^{20}$ = +21.4 (c = 1.0, $CHCl_3$); ^{1}H-NMR (300.13 MHz, $CDCl_3$): 7.40 – 7.16 (m, 12H), 6.89-6.83 (m, 3H), 6.25 (d, *J* = 1.6 Hz, 1H), 5.54 – 5.45 (m, 1H), 4.51 (brs, 1H), 3.82 (s, 3H), 3.16 (d, *J* = 13.8 Hz, 1H), 2.91 (dd, *J* = 13.8, J_2 = 0.7 Hz, 1H), 1.56 (s, 3H); ^{13}C-NMR (75.47 MHz, $CDCl_3$): 167.8, 156.4, 140.20, 140.17, 137.5, 134.5, 129.0, 128.63, 128.59, 128.3, 128.1, 128.0, 127.3, 127.1, 127.0, 120.8, 111.0, 77.6, 74.6, 55.2, 43.8, 27.1; IR (film) ῦ = 3465, 3063, 3032, 2928, 2851, 1716, 1694, 1623, 1600, 1583, 1488, 1454, 1436, 1398, 1362, 1295, 1280, 1234, 1152, 1121, 1060, 1047, 1026, 1001, 955, 812, 798, 753, 651, 591 cm^{-1}; HPLC analysis on chiral stationary phase {Daicel Chiralpak IA, *n*-heptane/2-propanol 90/10, 1.0 mL/min, 20 °C, UV 215 nm, t_{ret}(enantiomer 1) = 11.4 min, t_{ret}(enantiomer 2) = 14.9 min}: t_{ret}(major isomer) = 11.1 min, 77% ee; HRMS(ESI): *m/z*: calc. for $C_{26}H_{26}O_4Na^+$: 425.1723 $[M+Na]^+$, found: 425.1723.

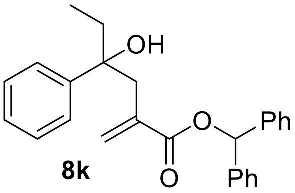

8k

Benzhydryl 4-hydroxy-2-methylene-4-phenylhexanoate (8k).

Procedure C: Propiophenone (14.0 mg, 100 µmol, 1 eq.); solvent: toluene/cyclohexane 2/1 (1.5 mL); flash chromatography (silica gel, hexanes/EtOAc 10/1); yield: 32.0 mg, 83.0 µmol, 83%, colorless oil; $[\alpha]_D^{20}$ = +25.4 (c = 1.0, $CHCl_3$); ^{1}H-NMR (300.13 MHz, $CDCl_3$): 7.42 – 7.23 (m, 14H), 7.23 – 7.14 (m, 1H), 6.87 (s, 1H), 6.26 (d, *J* = 1.4 Hz, 1H), 5.39 (d, *J* = 1.0 Hz, 1H), 3.36 (s, 1H), 2.97 (dd, J_1 = 14.0, J_2 = 0.7 Hz, 1H), 2.78 (dd, J_1 = 14.0, J_2 = 0.5 Hz, 1H), 1.97 – 1.72 (m, 2H), 0.75 (t, *J* = 7.4 Hz, 3H); ^{13}C-NMR (75.47 MHz, $CDCl_3$): 168.2, 145.3, 140.0, 136.5, 129.7, 128.67, 128.65, 128.15, 128.07, 127.97, 127.3, 127.2, 127.1, 126.4, 125.8, 77.9, 76.7, 45.6, 35.4, 7.9; IR (film) ῦ = 3459, 3088, 3062, 3031, 2967, 2929, 2878, 1696, 1624, 1494, 1447, 1299, 1123, 1080, 978, 961, 910, 813, 759, 741, 696, 592 cm^{-1}; HPLC analysis on chiral stationary phase {Daicel Chiralpak IC, *n*-heptane/2-propanol 98/2, 1.0 mL/min, 20 °C, UV 230 nm, t_{ret}(enantiomer 1) = 9.1 min, t_{ret}(enantiomer 2) = 13.1 min}: t_{ret}(major isomer) = 13.0 min, 92% ee; HRMS(ESI): *m/z*: calc. for $C_{26}H_{26}O_3Na^+$: 409.1774 $[M+na]^+$, found: 409.1773.

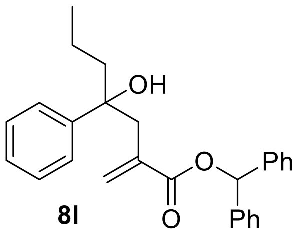

8l

Benzhydryl 4-hydroxy-2-methylene-4-phenylheptanoate (8l).

Procedure C: Butyrophenone (15.0 mg, 100 µmol, 1 eq.); solvent: toluene/cyclohexane 2/1 (1.5 mL); flash chromatography (silica gel, hexanes/EtOAc 10/1); yield: 37.0 mg, 92.0 µmol, 92%, colorless oil; $[\alpha]_D^{20}$ = +29.6 (c = 0.5, $CHCl_3$); ^{1}H-NMR (300.13 MHz, $CDCl_3$): 7.39 – 7.23 (m, 14H), 7.21 – 7.13 (m, 1H), 6.87 (s, 1H), 6.27 (d, *J* = 1.3 Hz, 1H), 5.40 (d, *J* = 0.9 Hz, 1H), 3.37 (br s, 1H), 2.97 (dd, J_1 = 14.0, J_2 = 0.4 Hz, 1H), 2.78 (d, *J* = 14.2 Hz, 1H), 1.79 (m, 2H), 1.44 – 1.24 (m, 1H), 1.13 – 0.92 (m, 1H), 0.81 (t, *J* = 7.3 Hz, 3H); ^{13}C-NMR (75.47 MHz, $CDCl_3$): 168.2, 145.7, 140.0, 136.5, 129.8, 128.68, 128.65, 128.2, 128.1, 128.0, 127.3, 127.1, 126.3, 125.6, 77.9, 76.6, 45.9, 45.1, 16.9, 14.5; IR (film) ũ = 3469 (br s), 3087, 3062, 3030, 2957, 2930, 2871, 1670, 1495, 1448, 1299, 1149, 1126, 1030, 953, 858, 813, 758, 741, 695, 591 cm^{-1}; HPLC analysis on chiral stationary phase {Daicel Chiralpak IC, *n*-heptane/2-propanol 98/2, 1.0 mL/min, 20 °C, UV 215 nm, t_{ret}(enantiomer 1) = 8.0 min, t_{ret}(enantiomer 2) = 12.2 min}: t_{ret}(major isomer) = 12.8 min, 93% ee; HRMS(ESI): *m/z*: calc. for $C_{27}H_{28}O_3Na^+$: 423.1931 [M+Na]$^+$, found: 423.1932.

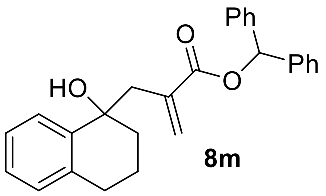

8m

Benzhydryl 2-[(1-hydroxy-1,2,3,4-tetrahydronaphthalen-1-yl)methyl]acrylate (8m).

Procedure C: α-Tetralone (15.0 mg, 100 µmol, 1 eq.); solvent: toluene/cyclohexane 2/1 (1.5 mL); flash chromatography (silica gel, hexanes/EtOAc 5/1); yield: 34.0 mg, 85.0 µmol, 85%, colorless oil; $[\alpha]_D^{20}$ = +35.5 (c = 1.0, $CHCl_3$); ^{1}H-NMR (300.13 MHz, $CDCl_3$): 7.54-7.51 (m, 1H), 7.41-7.27 (m, 10H), 7.17-7.12 (m, 2H), 7.06-7.03 (m, 1H), 6.94 (s, 1H), 6.49 (d, *J* = 1.4, 1H), 5.68 (d, *J* = 0.7, 1H), 3.23 (br s, 1H), 3.02 (d, *J* = 14.1, 1H), 2.78-2.74 (m, 3H), 1.97-1.90 (m, 1H), 1.87-1.74 (m, 3H); ^{13}C-NMR (75.47 MHz, $CDCl_3$): 168.0, 142.5, 140.2, 140.0, 136.9, 136.4, 129.9, 128.8, 128.7, 128.6, 128.1, 127.21, 127.19, 127.1, 126.7, 126.6, 126.2, 78.0, 72.2, 44.5, 35.7, 29.7, 20.1; IR (film) ũ = 3457 (br), 3063, 3030, 2936, 2836, 2869, 2838, 1713, 1494, 1451, 1300, 1233, 1141, 1080, 1022, 975, 951, 877, 759, 737, 696, 651, 618 cm^{-1}; HPLC analysis on chiral stationary phase {Daicel Chiralpak IA, *n*-heptane/2-propanol 80/20, 1.0 mL/min, 20 °C, UV 230 nm, t_{ret}(enantiomer 1) = 7.8 min, t_{ret}(enantiomer 2) = 13.1 min}: t_{ret}(major isomer) = 7.8 min, 93% ee; HRMS(ESI): *m/z*: calc. for $C_{27}H_{26}O_3Na^+$: 421.1774 [M+Na]$^+$, found: 421.1778.

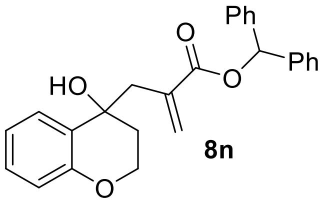

8n

Benzhydryl 2-[(4-hydroxychroman-4-yl)methyl]acrylate (8n).

Procedure C: 4-Chromanone (15.0 mg, 100 µmol, 1 eq.); solvent: toluene/cyclohexane 2/1 (1.5 mL); flash chromatography (silica gel, hexanes/EtOAc 5/1); yield: 36.0 mg, 90.0 µmol, 90%, colorless oil; $[\alpha]_D^{20}$ = +15.4 (c = 1.0, $CHCl_3$); ^{1}H-NMR (300.13 MHz, $CDCl_3$): 7.44 (dd, J_1 = 7.8, J_2 = 1.6 Hz, 1H), 7.40 – 7.27 (m, 10H), 7.15 (ddd, J_1 = 8.3, J_2 = 7.3, J_3 = 1.7 Hz, 1H), 6.90 (td, J_1 = 7.8, J_2 = 1.2 Hz, 1H), 6.80 (dd, J_1 = 8.2, J_2 = 1.1 Hz, 1H), 6.52 (d, *J* = 1.2 Hz, 1H), 5.71 (d, *J* = 0.6 Hz, 1H), 4.19 (t, *J* = 5.8 Hz, 2H), 3.20 (d, *J* = 13.9 Hz, 1H), 2.77 (d, *J* = 14.2 Hz, 1H), 1.97 (dd, J_1 = 11.2, J_2 = 4.9 Hz, 2H); ^{13}C-NMR (75.47 MHz, $CDCl_3$): 167.9, 154.0, 140.0, 139.9, 136.4, 130.5, 129.1, 128.7, 128.24, 128.21, 128.16, 127.20, 127.17, 126.8, 120.8, 117.1, 78.2, 68.0, 63.4, 43.9, 34.9; IR (film) ũ = 3457 (br), 3063, 3032, 2959, 2927, 2885, 1712, 1607, 1581, 1487, 1451, 1306, 1254, 1220, 1079, 1056, 975, 956, 908, 856, 804, 754, 733, 696, 591 cm^{-1}; HPLC analysis on chiral stationary phase {Daicel Chiralpak IA, *n*-heptane/2-propanol 80/20, 1.0 mL/min, 20 °C, UV 230 nm, t_{ret}(enantiomer 1) = 7.8 min, t_{ret}(enantiomer 2) = 13.1 min}: t_{ret}(major isomer) = 8.8 min, 97% ee; HRMS(ESI): *m/z*: calc. for $C_{26}H_{24}O_4Na^+$: 423.1567 [M+Na]$^+$, found: 423.1569.

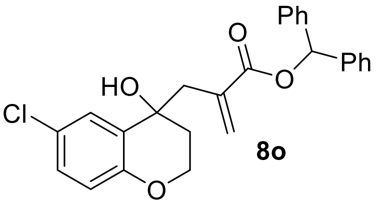

8o

Benzhydryl 2-[(6-chloro-4-hydroxychroman-4-yl)methyl]acrylate (8o).

Procedure C [but (*R*)-TRIP (0.1 eq. was used)]: 6-Chloro-4-Chromanone (18.3 mg, 100 µmol, 1 eq.); solvent: toluene/cyclohexane 2/1 (1.5 mL); flash chromatography (silica gel, hexanes/EtOAc 5/1); yield: 40.0 mg, 92.0 µmol, 92%, colorless oil; $[\alpha]_D^{20}$ = -16.8 (c = 1.0, $CHCl_3$); ^{1}H-NMR (300.13 MHz, $CDCl_3$): 7.41 (d, *J* = 2.6 Hz, 1H), 7.38 – 7.27 (m, 10H), 7.09 (dd, J_1 = 8.7, J_2 = 2.6 Hz, 1H), 6.96 (s, 1H), 6.73 (d, *J* = 8.8 Hz, 1H), 6.55 (d, *J* = 1.2 Hz, 1H), 5.72 (d, *J* = 1.1 Hz, 1H), 4.17 (td, J_1 = 5.7, J_2 = 1.4 Hz, 2H), 3.12 (dd, J_1 = 14.3, J_2 = 1.0 Hz, 1H), 2.74 (d, J = 14.2 Hz, 1H), 1.94 (ddd, J_1 = 6.2, J_2 = 4.4, J_3 = 2.7 Hz, 2H); ^{13}C-NMR (75.47 MHz, $CDCl_3$): 168.0, 152.6, 139.9, 139.8, 136.0, 131.0, 129.8, 129.1, 128.80, 128.76, 128.32, 128.28, 127.2, 126.7, 125.5, 118.5, 78.4, 67.9, 63.6, 44.1, 34.6; IR (film) ῦ = 3445 (br), 1710, 1624, 1483, 1455, 1411, 1295, 1253, 1224, 1149, 1093, 1780, 1052, 1031, 977, 957, 907, 814, 732, 697, 652 cm^{-1}; HPLC analysis on chiral stationary phase {Daicel Chiralpak IA, *n*-heptane/2-propanol 80/20, 1.0 mL/min, 30 °C, UV 230 nm, t_{ret}(enantiomer 1) = 7.2 min, t_{ret}(enantiomer 2) = 10.4 min}: t_{ret}(major isomer) = 10.5 min, 86% ee; HRMS(ESI): *m/z*: calc. for $C_{26}H_{23}ClO_4Na^+$: 457.1177 [M+Na]$^+$, found: 457.1176.

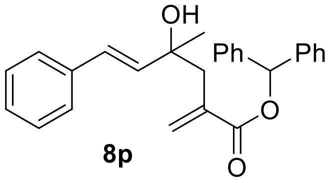

8p

Benzhydryl (*E*)-4-hydroxy-4-methyl-2-methylene-6-phenylhex-5-enoate (8p).

Procedure C: (*E*)-4-Phenylbut-3-en-2-one (15.0 mg, 100 µmol, 1 eq.); solvent: toluene/cyclohexane 2/1 (1.5 mL); flash chromatography (silica gel, hexanes/EtOAc 5/1); yield: 23.0 mg, 58.0 µmol, 58%, colorless oil; $[\alpha]_D^{20}$ = +15.0 (c = 1.0, $CHCl_3$); ^{1}H-NMR (300.13 MHz, $CDCl_3$): 7.46 – 7.13 (m, 15H), 6.86 (s, 1H), 6.54 (d, *J* = 16.0 Hz, 1H), 6.45 (d, *J* = 1.4 Hz, 1H), 6.23 (d, *J* = 16.0 Hz, 1H), 5.73 (d, *J* = 1.1 Hz, 1H), 2.85 (s, 1H), 2.79 (d, *J* = 13.8 Hz, 1H), 2.67 (d, *J* = 13.8 Hz, 1H), 1.36 (s, 3H); ^{13}C-NMR (75.47 MHz, $CDCl_3$): 167.6, 140.1, 140.0, 137.1, 136.8, 136.1, 129.7, 128.71, 128.67, 128.64, 128.5, 128.1, 127.4, 127.3, 127.2, 127.1, 126.6, 77.9, 72.6, 44.9, 28.5; IR (film) ῦ = 3461 (br), 3061, 3029, 2963, 2925, 2852, 1713, 1625, 1494, 1449, 1299, 1160, 965, 911, 853, 814, 743, 693, 652, 585 cm^{-1}; HPLC analysis on chiral stationary phase {Daicel Chiralpak IA, *n*-heptane/2-propanol 90/10, 1.0 mL/min, 20 °C, UV 230 nm, t_{ret}(enantiomer 1) = 11.0 min, t_{ret}(enantiomer 2) = 19.0 min}: t_{ret}(major isomer) = 10.7 min, 33% ee; HRMS(ESI): *m/z*: calc. for $C_{27}H_{26}O_3Na^+$: 421.1774 [M+Na]$^+$, found: 421.1776.

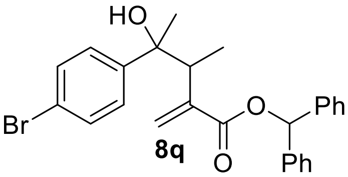

8q

Benzhydryl 4-(4-bromophenyl)-4-hydroxy-3-methyl-2-methylenepentanoate (8q).

Procedure C: 4'-Bromoacetophenone (10.0 mg, 50.0 µmol, 1 eq.); solvent: toluene (0.5 mL); flash chromatography (silica gel, hexanes/EtOAc 10/1); yield: 19.0 mg, 41.0 µmol, 82%, colorless oil; $[\alpha]_D^{20}$ = -10.5 (c = 1.0, $CHCl_3$); ^{1}H-NMR (300.13 MHz, $CDCl_3$): 7.46 – 7.27 (m, 15H), 6.95 (s, 1H), 6.47 (d, *J* = 0.8 Hz, 1H), 5.69 (s, 1H), 3.16 (q, *J* = 7.2 Hz, 1H), 1.37 (s, 3H), 0.89 (d, *J* = 7.2 Hz, 3H); ^{13}C-NMR (75.47 MHz, $CDCl_3$): 167.9, 145.9, 142.4, 140.0, 131.0, 128.8, 128.7, 128.3, 128.2, 128.0, 127.5, 127.3, 127.0, 126.7, 120.5, 78.1, 75.4, 46.8, 30.0, 14.6; IR (film) ῦ = 3444 (br), 3087, 3064, 3031, 2974, 2935, 2877, 1766, 1697, 1622, 1588, 1488, 1454, 1395, 1259, 1158, 1075, 1031, 1007, 955, 919, 822, 757, 740, 696, 588 cm^{-1}; HPLC analysis on chiral stationary phase {Daicel Chiralpak IA, *n*-heptane/2-propanol 90/10, 1.0 mL/min, 20 °C, UV 215 nm, t_{ret}(enantiomer 1) = 8.5 min, t_{ret}(enantiomer 2) = 12.6 min}: t_{ret}(major isomer) = 8.5 min, 89% ee; HRMS(ESI): *m/z*: calc. for $C_{27}H_{26}O_3Na^+$: 487.0879 [M+Na]$^+$, found: 487.0884.

General procedure for the asymmetric allylation of ketones with amide reagents **SI9** and **SI10** (Procedure D).

A 5 mL screw cap vial was charged with zinc (33.0 mg, 500 µmol, 5 eq.), NH_4Cl (43.0 mg, 800 µmol, 8 eq.) and (*R*)-3,3′-bis(2,4,6-triisopropylphenyl)-1,1′-binaphthyl-2,2′-diyl hydrogenphosphate ((*R*)-TRIP, 7.5 mg, 10.0 µmol, 0.1 eq.) followed by the respective solvent mixture (see compounds below for details). The ketone (100 µmol) and reagent **SI9** or **SI10** were added (see below for details). The mixture was stirred (720 rpm) at room temperature for 16 h, quenched by the addition of $NH_4Cl_{sat., aq.}$ solution (5 mL) and extracted with EtOAc (3 x 10 mL). The combined organic phase was dried over Na_2SO_4, filtered and concentrated under reduced pressure. The obtained crude product was purified via flash chromatography (SiO_2, eluent is indicated below) to give the pure product.

N,*N*-dibenzyl-4-hydroxy-2-methylene-4-phenylpentanamide (**8r**).

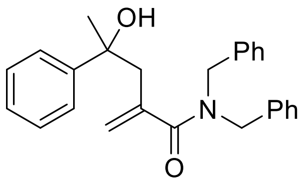

8r

Procedure D: Acetophenone (12.0 mg, 100 µmol, 1 eq.); *N*,*N*-dibenzyl-2-(bromomethyl)acrylamide (**7f**) (51.6 mg, 150 µmol, 1.5 eq.); solvent: toluene/pentane 1/1 (2 mL); preparative HPLC [Phenomenex LUNA AXIA™ pack (5 µm, C18(2), 100 Å, 250 x 21.2 mm), 30 mL/min flow, gradient eluent: H_2O/MeCN from 90/10 to 0/100 in 30 min.] yield compound **8r**: 11.6 mg, 30.0 µmol, 30%, colorless oil; $[\alpha]_D^{20}$ = -1.2 (c = 1.0, $CHCl_3$); ^{1}H NMR (300 MHz, Chloroform-*d*) δ 7.59 – 7.42 (m, 2H), 7.41 – 7.26 (m, 8H), 7.20 (tt, J_1 = 6.3, J_2 =1.3 Hz, 3H), 7.10 (d, J_1 = 6.6 Hz, 2H), 5.18 (d, *J* = 0.7 Hz, 1H), 4.90 (d, *J* = 1.0 Hz, 1H), 4.73 – 4.30 (m, 4H), 3.24 (s, 1H), 2.87 – 2.63 (m, 2H), 1.63 (s, 3H). ^{13}C NMR (75 MHz, $CDCl_3$) δ 174.5, 147.9, 138.7, 136.5, 136.2, 129.1, 128.8, 128.6, 128.0, 127.8, 127.0, 126.4, 125.3, 120.5, 74.2, 51.5, 49.2, 47.1, 30.7. IR (film) ṽ = 3310 (br), 3028, 2974, 1684, 1637, 1596, 1494, 1472, 1451, 1432, 1364, 1312, 1200, 1135, 1078, 1066, 1028, 909, 750, 728, 697, 665, 576, 490 cm^{-1}; HPLC analysis on chiral stationary phase {Diacel Chiralpack IA, *n*-heptane/2-propanol 95/5, 1.0 mL/min, 20 °C, UV 215 nm, t_{ret} (enantiomer 1) = 13.8 min, t_{ret} (enantiomer 2) = 17.0 min, t_{ret} (major isomer) = 13.9 min, 11% ee; HRMS(ESI): *m*/*z*: calc. for $C_{26}H_{28}NO_2^+$: 386.211294 $[M+H]^+$, found: 386.211456.

N,*N*-diethyl-4-hydroxy- 2-methylene-4-phenylpentanamide (**SI12**).

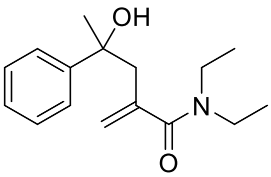

SI12

Procedure D: Acetophenone (9.6 mg, 80 µmol, 1 eq.); *N*,*N*-diethyl-2-(bromomethyl)acrylamide (**SI9**) (24.2 mg, 110 µmol, 1.35 eq.); solvent: toluene/pentane 1/1 (2 mL); no product could be observe.

Determination of absolute configuration and diastereoselectivity

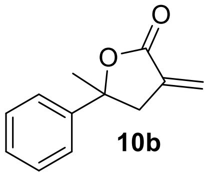

10b

(*S*)-5-Methyl-3-methylene-5-phenyldihydrofuran-2(3*H*)-one (**10b**).

Compound **8c** (12.6 mg, 34.0 µmol) was treated with *para*-toluenesulfonic acid (2.0 mg, 11.0 µmol) in $CHCl_3$ (500 µL) at room temperature for 16 h. The reaction mixture was quenched with brine (5 mL), extracted with $CHCl_3$ (3 x 10 mL), the combined organic phase was dried over Na_2SO_4, filtered and concentrated. The crude product was purified via flash chromatography (silica gel, cyclohexane/EtOAc 10/1) to give lactone **10b** (5.6 mg, 30 µmol, 88%) with the physical properties described above.

[α]$_D^{20}$ = +8.0 (c = 0.5, $CHCl_3$), lit.: [α]$_D^{20}$ = -11.3 (c = 1.2, $CHCl_3$) for the (*R*)-enantiomer (93% ee);[5] [α]$_D^{20}$ = -3.4 (c = 0.5, CH_2Cl_2), lit.: [α]$_D^{20}$ = -2.2 (c = 0.25, CH_2Cl_2) for the (*S*)-enantiomer (45% ee)];[6] HPLC analysis on chiral stationary phase {Daicel Chiralcel OJ, *n*-heptane/2-propanol 85/15, 0.7 mL/min, 20 °C, UV 215 nm, t_{ret}(enantiomer 1) = 18.4 min, t_{ret}(enantiomer 2) = 21.8 min}: t_{ret}(major isomer) = 17.2 min, 72% ee.

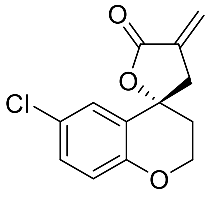

(*R*)-**10o**

Preparation of (*R*)-6-chloro-4'-methylene-3',4'-dihydro-5'H-spiro[chromane-4,2'-furan]-5'-one (10o) with DMAP.

Compound **8o** (96 mg, 221 µmol) was treated with 4-(dimethylamino)-pyridine (54 mg, 442 µmol) in EtOH (2.2 mL) at room temperature for 16 h. The reaction mixture was quenched with brine (5 mL), extracted with CH_2Cl_2 (3 x 15 mL), the combined organic phase was dried over Na_2SO_4, filtered and concentrated. The crude product was purified via flash chromatography (silica gel, cyclohexane/EtOAc 5/1) to give lactone (*R*)-**10o** (77 mg, 307 µmol, 90%) as a colorless oil.

[α]$_D^{20}$ = +79.8 (c = 1.1, $CHCl_3$); ^{1}H-NMR (300.13 MHz, $CDCl_3$): 7.19 – 7.14 (m, 2H), 6.79 (dd, J_1 = 8.4, J_2 = 0.7 Hz, 1H), 6.38 (t, J = 2.9 Hz, 1H), 5.78 (t, J = 2.5 Hz, 1H), 4.43 – 4.18 (m, 2H), 3.31 (dt, J_1 = 17.5, J_2 = 2.7 Hz, 1H), 3.03 (dt, J_1 = 17.5, J_2 = 2.7 Hz, 1H), 2.31 (ddd, J_1 = 14.2, J_2 = 6.5, J_3 = 3.1 Hz, 1H), 2.14 (ddd, J_1 = 14.2, J_2 = 8.8, J_3 = 3.7 Hz, 1H); ^{13}C-NMR (75.47 MHz, $CDCl_3$): 168.9, 153.3, 134.5, 130.6, 126.3, 126.0, 125.0, 123.5, 119.1, 77.4, 63.1, 41.2, 35.6; IR (film) $\tilde{\upsilon}$ = 1757, 1663, 1574, 1482, 1414, 1398, 1254, 1226, 1167, 1132, 1097, 1079, 1045, 1010, 974, 942, 883, 851, 787, 699, 687, 644, 604 cm^{-1}; HPLC analysis on chiral stationary phase {Daicel Chiralpak IA, *n*-heptane/2-propanol 80/20, 1.0 mL/min, 30 °C, UV 230 nm, t_{ret}(enantiomer 1) = 6.7 min, t_{ret}(enantiomer 2) = 7.4 min}: t_{ret}(major isomer) = 7.5 min, 86% ee; HRMS(ESI): *m/z*: calc. for $C_{13}H_{12}ClO_3^+$: 251.0469 $[M+H]^+$, found: 251.0471.

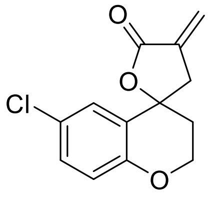

rac-**10o**

Preparation of *rac*-6-chloro-4'-methylene-3',4'-dihydro-5'H-spiro[chromane-4,2'-furan]-5'-one (10o) with *p*TSA.

Compound **8o** (146 mg, 340 µmol) was treated with *para*-toluenesulfonic acid monohydrate (20 mg, 110 µmol) in CH_2Cl_2 (700 µL) at room temperature for 16 h. The reaction mixture was quenched with brine (5 mL), extracted with CH_2Cl_2 (3 x 15 mL), the combined organic phase was dried over Na_2SO_4, filtered and concentrated. The crude product was purified via flash chromatography (silica gel, cyclohexane/EtOAc 5/1) to give lactone *rac*-**10o** (83 mg, 330 µmol, 97%) as a colorless oil.

All physical data was in accordance with the one reported above. No optical rotation was observed. HPLC analysis on chiral stationary phase {Daicel Chiralpak IA, *n*-heptane/2-propanol 80/20, 1.0 mL/min, 30 °C, UV 230 nm, t_{ret}(enantiomer 1) = 6.7 min, t_{ret}(enantiomer 2) = 7.4 min}: t_{ret}(major isomer) = 6.7 min, 3% ee.

Crystallization of 10o.

Crystals of lactone **10o** were grown from diethylether/pentane (1/1) at room temperature over 1 d: Compound **10o** [70 mg of (*R*)-**10o** and 75 mg *rac*-**10o**] was dissolved in diethylether (ca. 500 µL) and the solution was covered by a layer of pentane (ca. 500 µL). The crystallization was induced by slow diffusion of the pentane into the solution of **10o** and crystalline material was obtained the next day. Two samples were crystallized and three crystal structures determined:

rac-**10o**: *rac*-**10o**-Cry1, 50% ee [(*S*)-enantiomer] for the picked single crystal.

rac-**10o**-Cry2, 98% ee [(*S*)-enantiomer] for the picked single crystal.

(*R*)-**10o**: (*R*)-**10o**-Cry1, > 99% ee [(*R*)-enantiomer] for the picked single crystal.

Each single crystal was redissolved in 2-PrOH and subjected to HPLC-UV analysis on a chiral stationary phase after the structure elucidation (see HPLC data below).

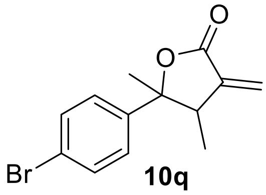

10q

5-(4-bromophenyl)-4,5-dimethyl-3-methylenedihydrofuran-2(3H)-one (**10q**).

Compound **8q** (12.6 mg, 34.0 µmol) was treated with *para*-toluenesulfonic acid (2.0 mg, 11 µmol) in $CHCl_3$ (500 µL) at room temperature for 16 h. The reaction mixture was quenched with brine (5 mL), extracted with $CHCl_3$ (3 x 10 mL), the combined organic phase was dried over Na_2SO_4, filtered and concentrated. The crude product was purified via flash chromatography (silica gel, cyclohexane/EtOAc 10/1) to give lactone **10q** (3.1 mg, 30 µmol, 88%).

$[\alpha]_D^{20}$ = -10.0 (c = 0.3, Me); ^{1}H-NMR (300.13 MHz, $CDCl_3$): 7.51 – 7.46 (m, 2H), 7.13 – 7.08 (m, 2H), 6.28 (d, *J* = 2.7 Hz, 1H), 5.52 (d, *J* = 2.4 Hz, 1H), 3.08 (qt, J_1 = 7.0, J_2 = 2.5 Hz, 1H), 1.78 (s, 3H), 0.79 (d, *J* = 7.0 Hz, 3H); ^{13}C-NMR (75.47 MHz, $CDCl_3$): 170.0, 141.0, 139.6, 131.6, 127.2, 122.1, 122.0, 87.1, 46.0, 28.3, 16.5; IR (film) ῦ = 1759, 1489, 1451, 1396, 1379, 1255, 1194, 1110, 1074, 1057, 1007, 938, 823, 813, 737, 503; HPLC analysis on chiral stationary phase {Daicel Chiralcel OJ, *n*-heptane/2-propanol 85/15, 0.7 mL/min, 20 °C, UV 230 nm, t_{ret}(enantiomer 1) = 14.5 min, t_{ret}(enantiomer 2) = 18.5 min}: t_{ret}(major isomer) = 18.5 min, 75% ee; HRMS(ESI): *m*/*z*: calc. for $C_{13}H_{14}O_2Br^+$: 281.0172 [M+H]$^+$, found: 281.0174.

Investigations of the origin of the switch and the responsible structural motif

General procedure for the asymmetric allylation of ketones with the lactone based organozinc reagent.

The lactone-based organozinc reagent was prepared according to literature.[11]

A 20 mL microwave-tube (Biotage) was charged with zinc dust (164.0 mg, 2.5 mmol, 5 eq.), NH_4Cl (215.0 mg, 4.0 mmol, 8 eq.) and (*R*)-3,3′-bis(2,4,6-triisopropylphenyl)-1,1′-binaphthyl-2,2′-diyl hydrogenphosphate (TRIP, 37.8 mg, 50.2 µmol, 0.1 eq.) followed by toluene (5.0 mL). The 4-bromobenzaldehyde or 4-bromoacetophenone (502 µmol) and 3-(bromomethyl)furan-2(5*H*)-one **18** (89.0 mg, 502 µmol, 1 eq.) were added. The vial was sealed with a crimp-cap and the mixture was stirred (720 rpm) at room temperature for 16 h, quenched by the addition of $NH_4Cl_{sat., aq.}$ solution (25 mL) and extracted with EtOAc (3 x 50 mL). The combined organic phase was dried over Na_2SO_4, filtered and concentrated under reduced pressure. The obtained crude product was purified via preparative HPLC according to following procedure to give the pure product:

A Shimadzu prominence preparative liquid chromatograph system equipped with a 250 x 21.2 mm Phenomenex Luna C18(2) 100 Å column and a Shimadzu SPD-M20A PDA detector was used. The pure product was obtained running a gradient from 10% MeCN in H_2O to 100% MeCN over 23 min with a flow of 30 mL/min. The sample fractioning was done manually.

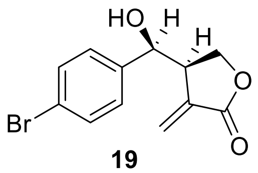

19

4-[(4-bromophenyl)(hydroxy)methyl]-3-methylenedihydrofuran-2(3*H*)-one (**19**).

4-Bromobenzaldehyde (93.0 mg, 502 µmol, 1 eq.); yield: 48.0 mg, 169 µmol, 34%, colorless oil; $[\alpha]_D^{20}$ = +11.1 (c = 0.9, $CHCl_3$); ^{1}H-NMR (300.13 MHz, $CDCl_3$): 7.56–7.50 (m, 2H), 7.25–7.20 (m, 2H), 6.37 (d, J = 2.2, 1H), 5.75 (d, J = 1.9, 1H), 4.71 (d, J = 7.4, 1H), 4.21 (d, J = 8.3, 0.4H), 4.18 (d, J = 8.2, 0.6H), 4.09 (d, J = 4.1, 0.6H), 4.06 (d, J = 4.1, 0.4H), 3.41–3.33 (m, 1H), 2.04 (bs, 1H); ^{13}C-NMR (75.47 MHz, $CDCl_3$): 170.7, 139.7, 134.7, 132.1 (2C), 128.4 (2C), 125.8, 122.7, 75.1, 67.6, 45.5; IR (film) ῦ = 3434 (br), 2974, 2912, 1741, 1657, 1591, 1486, 1401, 1317, 1271, 1210, 1188, 1116, 1070, 1009, 949, 909, 818, 730, 623 cm^{-1}; HPLC analysis on chiral stationary phase {Daicel Chiralpak IE, *n*-heptane/2-propanol 90/10, 0.7 mL/min, 10 °C, UV 230 nm, t_{ret}(enantiomer 1) = 33.3 min, t_{ret}(enantiomer 2) = 43.6 min}: t_{ret}(major isomer) = 43.0 min, 93% ee; HRMS(ESI): *m/z*: calc. for $C_{12}H_{12}BrO_3^+$: 282.9965 and 284.9944 [M+H]$^+$, found: 282.9964 and 284.9945 (for the two most prominent isotopes).

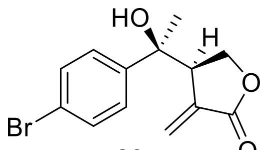

20

4-[1-(4-bromophenyl)-1-hydroxyethyl]-3-methylenedihydrofuran-2(3*H*)-one (**20**).

4-Bromoacetophenone (100.0 mg, 502 µmol, 1 eq.); yield: 10.4 mg, 35.0 µmol, 7%, colorless oil; $[\alpha]_D^{20}$ = +35.7 (c = 1.05, $CHCl_3$); ^{1}H-NMR (300.13 MHz, $CDCl_3$): 7.54–7.47 (m, 2H), 7.34–7.28 (s, 2H), 6.27 (d, *J* = 2.3, 1H), 5.27 (d, *J* = 1.9, 1H), 4.37 (d, J = 4.0, 0.3H), 4.34 (d, J = 4.0, 0.7H), 4.30 (d, J = 8.0, 0.7H, 4.26 (d, J = 8.1, 0.3H), 3.44–3-38 (m, 1H), 2.01 (bs, 1H), 1.58 (s, 3H). ^{13}C-NMR (75.47 MHz, $CDCl_3$):170.8, 143.8, 134.5, 131.8 (2C), 127.3 (2C), 125.9, 122.0, 104.1 (rotamer 1), 103.1 (rotamer 2), 75.5, 67.1, 49.3, 26.3, 18.6 (rotamer 1), 18.2 (romater 2); IR (film) ῦ = 3415 (br), 2993,2939, 1744, 1488, 1446, 1395, 1375, 1319, 1277, 1243, 1221, 1127, 1078, 1041, 1008, 953, 823, 752 cm^{-1}; HPLC analysis on chiral stationary phase {Daicel Chiralpak IA, *n*-heptane/2-propanol 85/15, 0.7 mL/min, 18 °C, UV 230 nm, t_{ret}(enantiomer 1) = 13.7 min, t_{ret}(enantiomer 2) = 15.6 min}: t_{ret}(major isomer) = 13.7 min, 97% ee; HRMS(ESI): *m/z*: calc. for $C_{13}H_{14}BrO_3^+$: 297.0121 and 299.0101 [M+H$^+$], found: 297.0125 and 299.0107 (for the two most prominent isotopes).

Racemic reference material for HPLC analysis on a chiral stationary phase was prepared according to procedure D with diphenylphosphate instead of TRIP catalyst in toluene/1,2-dimethoxyethane 1:1 at 70 °C under otherwise identical conditions: *tert*. alcohol **20**: 3.3 mg, 11.1 µmol, 2.2%; *sec*. alcohol **19**: 107 mg, 378 µmol, 75%.

CD spectra of compounds 19 and 20.

The CD spectra were recorded on a Jasco J-1500 CD Spectrometer instrument. The spectra determined from 190-350 nm at 20 °C in a 1 mm quartz cuvette. The samples were dissolved in MeOH (final concentration: 1.0 mg/mL).

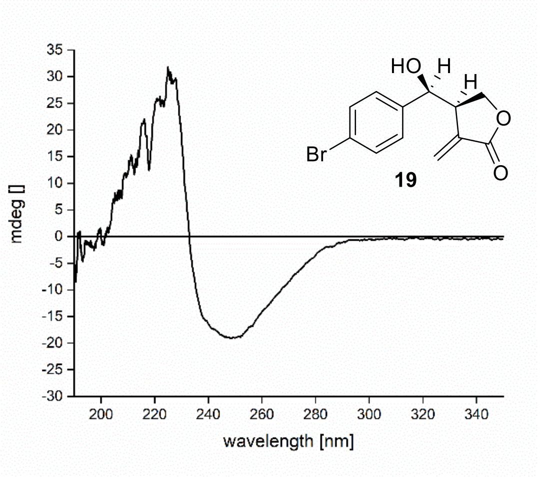

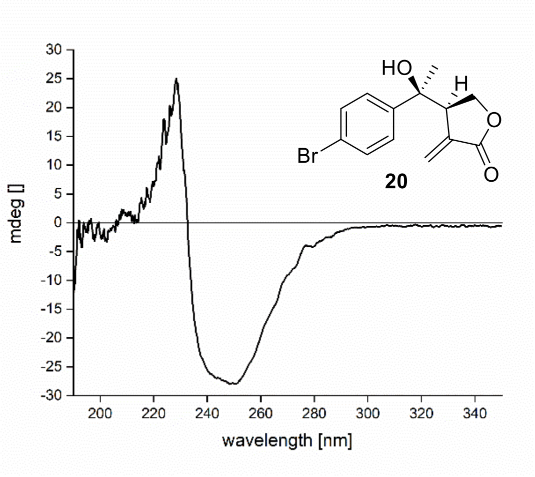

Crystal Structure Determination of 10o.

rac-10o-Cry1

Crystal Structure Determination of *rac*-10o-Cry1. All the measurements were performed using monochromatized Mo K_α radiation at 100K: $C_{13}H_{11}ClO_3$, M_r 250.67, orthorhombic, space group P 2_1 2_1 2_1, a = 6.4316(4)Å, b = 7.3722(5)Å, c = 23.2745(14)Å, V = 1103.56(12)Å^3, Z = 4, d_{calc} = 1.509g cm^{-3}, μ = 0.338mm^{-1}. A total of 21090 reflections were collected (Θ_{max} = 40.0°), from which 6826 were unique (R_{int} = 0.0263), with 6475 having I > 2σ(I). The structure was solved by direct methods (SHELXS-97)[7] and refined by full-matrix least-squares techniques against F^2 (SHELXL-2014/6)[8]. The non-hydrogen atoms were refined with anisotropic displacement parameters without any constraints. The absolute configuration was established by anomalous dispersion effects in the diffraction measurements on the crystal. The structure was refined as a 2-component inversion twin resulting in a scale factor of 0.32(4) for the fractional contribution of the less prominent twin component [hence 32(4)% (4*R*)-enantiomer]. The H atoms of the terminal CH_2 group were refined with a common isotropic displacement parameter and idealized geometry with the hydrogen atoms in the plane through the atoms C13, C15, C16 and C–H distances of 0.95Å. The H atoms of the other CH_2 groups were refined with common isotropic displacement parameters for the H atoms of the same group and idealized geometry with approximately tetrahedral angles and C–H distances of 0.99Å. The H atoms of the phenyl ring were put at the external bisectors of the C–C–C angles at C–H distances of 0.95Å and a common isotropic displacement parameter was refined for these H atoms. For 160 parameters final *R* indices of R1 = 0.0294 and wR^2 = 0.0792 (GOF = 1.088) were obtained. The largest peak in a difference Fourier map was 0.531eÅ^{-3}.

Results and Discussion

Crystal Structure. The crystal structure analysis of *rac*-**10o**-Cry1 confirmed the compound as (4*S*/4*R*)-6-chloro-4'-methylene-2,3,3',4'-tetrahydro-5'*H*-spiro[chromene-4,2'-furan]-5'-one [4*S*:4*R* = 68(4):32(4)%]. All atoms lie on general positions. The determination of the absolute configuration from anomalous dispersion effects resulted in a Flack-parameter[9] of 0.32(4), hence in an enantiomeric ratio (4*S*):(4*R*) of 68(4):32(4)% .

Table SI01. Crystal data and structure refinement for *rac*-**10o**-Cry1.

Crystal data	
CCDC number	1944605
Identification code	FE111
Empirical formula	$C_{13}H_{11}ClO_3$
Formula weight	250.67
Crystal description	block, colourless
Crystal size	0.43 x 0.35 x 0.32mm
Crystal system, space group	orthorhombic, P 2_1 2_1 2_1
Unit cell dimensions: a	6.4316(4)Å
b	7.3722(5)Å
c	23.2745(14)Å
Volume	1103.56(12)Å^3
Z	4
Calculated density	1.509Mg/m^3
F(000)	520
Linear absorption coefficient μ	0.338mm^{-1}
Absorption correction	semi-empirical from equivalents
Max. and min. transmission	1.000 and 0.870
Unit cell determination	2.90° < Θ < 40.72°
	9936 reflections used at 100K
Data collection	
Temperature	100K
Diffractometer	Bruker APEX-II CCD
Radiation source	Incoatec microfocus sealed tube
Radiation and wavelength	MoK$_\alpha$, 0.71073Å
Monochromator	multilayer monochromator
Scan type	ϕ and ω scans
Θ range for data collection	2.90 to 40.00°
Reflections collected / unique	21090 / 6826
Significant unique reflections	6475 with I > 2σ(I)
R(int), R(sigma)	0.0263, 0.0266
Completeness to Θ = 40.0°	99.9%
Refinement	
Refinement method	Full-matrix least-squares on F^2
Data / parameters / restraints	6826 / 160 / 0
Goodness-of-fit on F^2	1.088
Final R indices [I > 2σ(I)]	R1 = 0.0294, wR2 = 0.0777
R indices (all data)	R1 = 0.0317, wR2 = 0.0792
Absolute structure parameter	0.32(4)
Extinction expression	none
Weighting scheme	$w = 1/[\sigma^2(F_o^2)+(aP)^2+bP]$ where $P = (F_o^2+2F_c^2)/3$
Weighting scheme parameters a, b	0.0471, 0.0547
Largest Δ/σ in last cycle	0.003
Largest difference peak and hole	0.531 and -0.185e/Å^3
Structure Solution Program	SHELXS-97 (Sheldrick, 2008)
Structure Refinement Program	SHELXL-2014/6 (Sheldrick, 2015)

Table SI02. Atomic coordinates and equivalent isotropic displacement parameters (Å^2) for *rac*-**10o**-Cry1. U_{eq} is defined as one third of the trace of the orthogonalized U_{ij} tensor.

	x	y	z	U_{eq}
O1	0.77565(12)	0.80027(11)	0.62041(3)	0.01506(12)
C2	0.78949(17)	0.69698(14)	0.56812(4)	0.01565(16)
C3	0.73056(15)	0.50165(14)	0.57929(4)	0.01383(14)
C4	0.50451(14)	0.48750(11)	0.59804(3)	0.00955(12)
C5	0.27255(14)	0.63032(12)	0.67393(4)	0.01031(12)
C6	0.23722(13)	0.75848(13)	0.71613(3)	0.01102(12)
C7	0.38129(15)	0.89646(13)	0.72690(4)	0.01244(14)
C8	0.55946(15)	0.90539(13)	0.69379(4)	0.01250(14)
C9	0.59593(14)	0.77814(11)	0.65027(3)	0.01046(13)
C10	0.45492(13)	0.63619(11)	0.64077(3)	0.00915(12)
O11	0.37416(11)	0.51686(10)	0.54675(3)	0.01185(11)
C13	0.44875(15)	0.29384(12)	0.61895(4)	0.01082(13)
C14	0.25983(14)	0.24392(13)	0.58499(3)	0.01101(12)
C15	0.23734(14)	0.38040(12)	0.53844(3)	0.01051(13)
O15	0.11852(12)	0.38056(11)	0.49804(3)	0.01497(13)
C16	0.12672(17)	0.10704(15)	0.59140(4)	0.01805(17)
Cl1	0.01630(4)	0.74311(3)	0.75900(2)	0.01614(5)

Table SI03. Hydrogen coordinates and isotropic displacement parameters (Å^2) for *rac*-**10o**-Cry1.

	x	y	z	U_{iso}
H21	0.9332	0.7027	0.5530	0.022(3)
H22	0.6951	0.7494	0.5389	0.022(3)
H31	0.8215	0.4510	0.6096	0.018(3)
H32	0.7518	0.4296	0.5439	0.018(3)
H5	0.1728	0.5376	0.6673	0.022(3)
H7	0.3571	0.9827	0.7565	0.022(3)
H8	0.6581	0.9988	0.7006	0.022(3)
H131	0.5641	0.2083	0.6113	0.023(3)
H132	0.4182	0.2934	0.6606	0.023(3)
H161	0.0128	0.0943	0.5657	0.030(4)
H162	0.1457	0.0221	0.6216	0.030(4)

Table SI04. Anisotropic displacement parameters (Å^2) for *rac*-**10o**-Cry1. The anisotropic displacement factor exponent takes the form: $-2\pi^2[h^2 a^{*2}U_{11} + ... + 2 h k a^* b^* U_{12}]$.

	U_{11}	U_{22}	U_{33}	U_{23}	U_{13}	U_{12}
O1	0.0132(3)	0.0148(3)	0.0172(3)	-0.0027(2)	0.0037(2)	-0.0063(2)
C2	0.0163(4)	0.0160(4)	0.0146(3)	-0.0007(3)	0.0044(3)	-0.0049(3)
C3	0.0117(3)	0.0133(3)	0.0165(3)	-0.0021(3)	0.0028(3)	-0.0014(3)
C4	0.0105(3)	0.0090(3)	0.0092(2)	-0.0001(2)	-0.0009(2)	-0.0016(3)
C5	0.0104(3)	0.0090(3)	0.0116(3)	-0.0003(2)	0.0002(2)	-0.0008(2)
C6	0.0111(3)	0.0105(3)	0.0115(3)	0.0001(2)	0.0004(2)	0.0015(3)
C7	0.0146(4)	0.0103(3)	0.0125(3)	-0.0022(2)	-0.0019(3)	0.0014(3)
C8	0.0134(3)	0.0098(3)	0.0143(3)	-0.0017(2)	-0.0025(3)	-0.0020(3)
C9	0.0108(3)	0.0088(3)	0.0117(3)	0.0002(2)	-0.0003(2)	-0.0024(2)
C10	0.0096(3)	0.0083(3)	0.0095(2)	0.0001(2)	-0.0005(2)	-0.0012(2)
O11	0.0154(3)	0.0106(3)	0.0096(2)	0.00120(18)	-0.0027(2)	-0.0031(2)
C13	0.0131(3)	0.0084(3)	0.0110(3)	0.0004(2)	-0.0012(2)	-0.0008(2)
C14	0.0124(3)	0.0099(3)	0.0107(2)	-0.0004(2)	0.0003(2)	-0.0019(3)
C15	0.0110(3)	0.0107(3)	0.0098(3)	-0.0013(2)	0.0004(2)	-0.0010(3)
O15	0.0144(3)	0.0181(3)	0.0124(2)	-0.0008(2)	-0.0039(2)	-0.0013(2)
C16	0.0184(4)	0.0169(4)	0.0189(4)	0.0032(3)	-0.0023(3)	-0.0080(3)
Cl1	0.01533(9)	0.01483(9)	0.01825(9)	-0.00059(7)	0.00599(7)	0.00253(8)

CCDC 1944605 contains the supplementary crystallographic data for this structure. This data can be obtained free of charge via http://www.ccdc.cam.ac.uk/conts/retrieving.html (or from the CCDC, 12 Union Road, Cambridge CB2 1EZ, UK; Fax: +44 1223 336033; E-mail: deposit@ccdc.cam.ac.uk).

Table SI05. Full list of bond lengths [Å] and angles [°] for *rac*-**10o**-Cry1.

O1-C9	1.3586(11)
O1-C2	1.4383(12)
C2-C3	1.5116(15)
C2-H21	0.99
C2-H22	0.99
C3-C4	1.5215(13)
C3-H31	0.99
C3-H32	0.99
C4-O11	1.4747(10)
C4-C10	1.5140(11)
C4-C13	1.5504(12)
C5-C6	1.3817(12)
C5-C10	1.4047(12)
C5-H5	0.95
C6-C7	1.3987(13)
C6-Cl1	1.7400(9)
C7-C8	1.3826(14)
C7-H7	0.95
C8-C9	1.4003(12)
C8-H8	0.95
C9-C10	1.4024(12)
O11-C15	1.3505(11)
C13-C14	1.4956(12)
C13-H131	0.99
C13-H132	0.99
C14-C16	1.3318(14)
C14-C15	1.4855(13)
C15-O15	1.2116(11)
C16-H161	0.95
C16-H162	0.95

C9-O1-C2	114.95(7)
O1-C2-C3	110.08(8)
O1-C2-H21	109.6
C3-C2-H21	109.6
O1-C2-H22	109.6
C3-C2-H22	109.6
H21-C2-H22	108.2
C2-C3-C4	110.75(8)
C2-C3-H31	109.5
C4-C3-H31	109.5
C2-C3-H32	109.5
C4-C3-H32	109.5
H31-C3-H32	108.1
O11-C4-C10	107.80(7)
O11-C4-C3	107.52(6)
C10-C4-C3	109.88(7)
O11-C4-C13	104.94(6)
C10-C4-C13	114.31(6)
C3-C4-C13	111.98(8)
C6-C5-C10	120.45(8)
C6-C5-H5	119.8
C10-C5-H5	119.8
C5-C6-C7	121.05(8)
C5-C6-Cl1	119.83(7)
C7-C6-Cl1	119.05(7)
C8-C7-C6	118.93(8)
C8-C7-H7	120.5
C6-C7-H7	120.5
C7-C8-C9	120.68(8)
C7-C8-H8	119.7
C9-C8-H8	119.7
O1-C9-C8	115.60(8)
O1-C9-C10	124.00(8)
C8-C9-C10	120.37(8)
C9-C10-C5	118.45(7)
C9-C10-C4	120.51(7)
C5-C10-C4	120.96(7)
C15-O11-C4	112.15(7)
C14-C13-C4	104.39(7)
C14-C13-H131	110.9
C4-C13-H131	110.9
C14-C13-H132	110.9
C4-C13-H132	110.9
H131-C13-H132	108.9
C16-C14-C15	122.16(8)
C16-C14-C13	130.50(9)
C15-C14-C13	107.33(7)
O15-C15-O11	121.43(8)
O15-C15-C14	128.90(9)
O11-C15-C14	109.67(7)
C14-C16-H161	120.0
C14-C16-H162	120.0
H161-C16-H162	120.0

C9-O1-C2-C3	-48.83(12)
O1-C2-C3-C4	63.85(11)
C2-C3-C4-O11	74.18(9)
C2-C3-C4-C10	-42.88(10)
C2-C3-C4-C13	-171.05(7)
C10-C5-C6-C7	-0.06(13)
C10-C5-C6-Cl1	-177.05(6)
C5-C6-C7-C8	1.24(13)
Cl1-C6-C7-C8	178.26(7)
C6-C7-C8-C9	-0.24(14)
C2-O1-C9-C8	-166.40(8)
C2-O1-C9-C10	15.47(13)
C7-C8-C9-O1	179.87(8)
C7-C8-C9-C10	-1.94(13)
O1-C9-C10-C5	-178.90(8)
C8-C9-C10-C5	3.06(12)
O1-C9-C10-C4	4.42(13)
C8-C9-C10-C4	-173.62(8)
C6-C5-C10-C9	-2.08(12)
C6-C5-C10-C4	174.59(8)
O11-C4-C10-C9	-106.33(8)
C3-C4-C10-C9	10.56(10)
C13-C4-C10-C9	137.43(8)
O11-C4-C10-C5	77.07(9)
C3-C4-C10-C5	-166.04(8)
C13-C4-C10-C5	-39.17(11)
C10-C4-O11-C15	-115.59(8)
C3-C4-O11-C15	126.00(8)
C13-C4-O11-C15	6.63(9)
O11-C4-C13-C14	-11.31(8)
C10-C4-C13-C14	106.58(8)
C3-C4-C13-C14	-127.64(8)
C4-C13-C14-C16	-167.92(10)
C4-C13-C14-C15	12.08(9)
C4-O11-C15-O15	-179.36(8)
C4-O11-C15-C14	1.03(10)
C16-C14-C15-O15	-8.20(16)
C13-C14-C15-O15	171.79(9)
C16-C14-C15-O11	171.37(9)
C13-C14-C15-O11	-8.64(10)

rac-**10o**-Cry2

All the measurements were performed using monochromatized Mo K_{α} radiation at 100K: $C_{13}H_{11}ClO_3$, M_r 250.67, orthorhombic, space group P 2_1 2_1 2_1, a = 6.4281(3)Å, b = 7.3763(3)Å, c = 23.3211(9)Å, V = 1105.78(8)Å^3, Z = 4, d_{calc} = 1.506g cm^{-3}, μ = 0.337mm^{-1}. A total of 20844 reflections were collected (Θ_{max} = 39.9°), from which 6851 were unique (R_{int} = 0.0267), with 6631 having I > 2σ(I). The structure was solved by direct methods (SHELXS-97)[7] and refined by full-matrix least-squares techniques against F^2 (SHELXL-2014/6)[8]. The non-hydrogen atoms were refined with anisotropic displacement parameters without any constraints. The absolute configuration was established by anomalous dispersion effects in the diffraction measurements on the crystal. The H atoms of the terminal CH_2 group were refined with a common isotropic displacement parameter and idealized geometry with the hydrogen atoms in the plane through the atoms C13, C15, C16 and C–H distances of 0.95Å. The H atoms of the other CH_2 groups were refined with common isotropic displacement parameters for the H atoms of the same group and idealized geometry with approximately tetrahedral angles and C–H distances of 0.99Å. The H atoms of the phenyl ring were put at the external bisectors of the C–C–C angles at C–H distances of 0.95Å and a common isotropic displacement parameter was refined for these H atoms. For 159 parameters final *R* indices of R1 = 0.0269 and wR^2 = 0.0738 (GOF = 1.135) were obtained. The largest peak in a difference Fourier map was 0.500eÅ^{-3}.

Results and Discussion

Crystal Structure. The crystal structure analysis of *rac*-**10o**-Cry2 confirmed the compound as (4*S*)-6-chloro-4'-methylene-2,3,3',4'-tetrahydro-5'*H*-spiro[chromene-4,2'-furan]-5'-one. All atoms lie on general positions (s. Fig. SI01). The determination of the absolute configuration from anomalous dispersion effects resulted in a Flack-parameter[9] of 0.03(4).

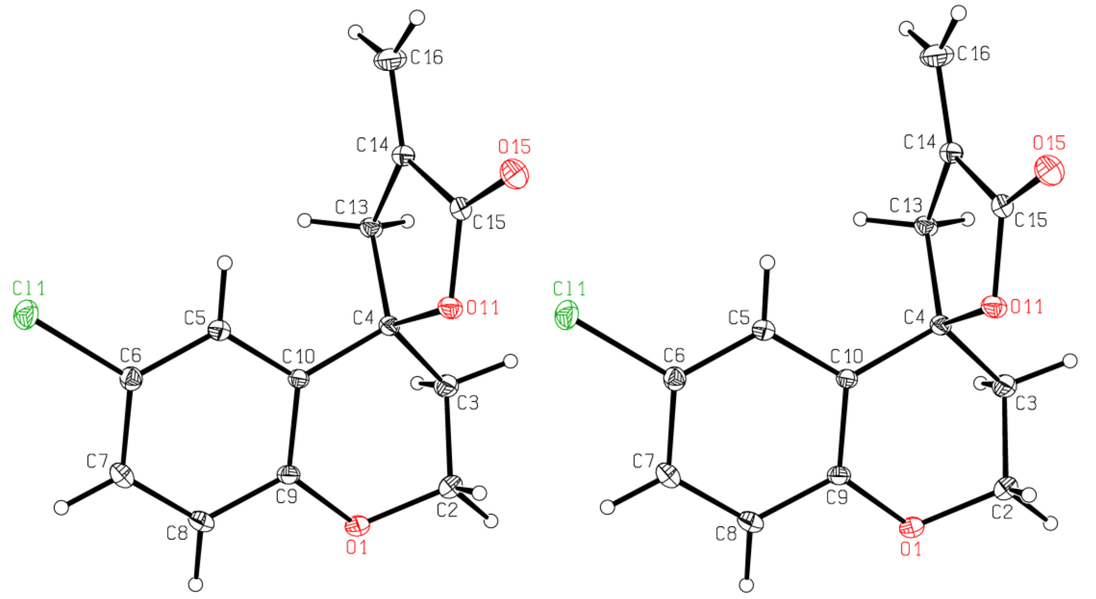

Figure SI01. Stereoscopic ORTEP[10] plot of *rac*-**10o**-Cry2 showing the atomic numbering scheme. The probability ellipsoids are drawn at the 50% probability level. The H atoms are drawn with arbitrary radii.

Table SI06. Crystal data and structure refinement for *rac*-**10o**-Cry2.

Crystal data	
CCDC number	1944606
Identification code	FE111B
Empirical formula	$C_{13}H_{11}ClO_3$
Formula weight	250.67
Crystal description	block, colourless
Crystal size	0.44 x 0.44 x 0.35mm
Crystal system, space group	orthorhombic, P 2_1 2_1 2_1
Unit cell dimensions: a	6.4281(3)Å
b	7.3763(3)Å
c	23.3211(9)Å
Volume	1105.78(8)$Å^3$
Z	4
Calculated density	1.506Mg/m^3
F(000)	520
Linear absorption coefficient μ	0.337mm^{-1}
Absorption correction	semi-empirical from equivalents
Max. and min. transmission	1.000 and 0.880
Unit cell determination	2.76° < Θ < 40.66°<br>9939 reflections used at 100K
Data collection	
Temperature	100K
Diffractometer	Bruker APEX-II CCD
Radiation source	Incoatec microfocus sealed tube
Radiation and wavelength	MoK_α, 0.71073Å
Monochromator	multilayer monochromator
Scan type	ϕ and ω scans
Θ range for data collection	2.90 to 39.99°
Reflections collected / unique	20844 / 6851
Significant unique reflections	6631 with I > 2σ(I)
R(int), R(sigma)	0.0267, 0.0269
Completeness to Θ = 39.99°	99.9%
Refinement	
Refinement method	Full-matrix least-squares on F^2
Data / parameters / restraints	6851 / 159 / 0
Goodness-of-fit on F^2	1.135
Final R indices [I > 2σ(I)]	R1 = 0.0269, wR2 = 0.0732
R indices (all data)	R1 = 0.0281, wR2 = 0.0738
Absolute structure parameter	0.03(4)
Extinction expression	none
Weighting scheme	$w = 1/[\sigma^2(F_o^2)+(aP)^2+bP]$ where $P = (F_o^2+2F_c^2)/3$
Weighting scheme parameters a, b	0.0396, 0.0669
Largest Δ/σ in last cycle	0.003
Largest difference peak and hole	0.500 and -0.294e/$Å^3$
Structure Solution Program	SHELXS-97 (Sheldrick, 2008)
Structure Refinement Program	SHELXL-2014/6 (Sheldrick, 2015)

Table SI07. Atomic coordinates and equivalent isotropic displacement parameters (Å^2) for *rac*-**10o**-Cry2. U_{eq} is defined as one third of the trace of the orthogonalized U_{ij} tensor.

	x	y	z	U_{eq}
O1	0.77586(12)	0.80028(9)	0.62044(3)	0.01466(12)
C2	0.78972(16)	0.69701(13)	0.56815(4)	0.01513(15)
C3	0.73090(14)	0.50160(12)	0.57930(4)	0.01346(14)
C4	0.50481(13)	0.48715(10)	0.59799(3)	0.00916(11)
C5	0.27257(13)	0.62994(10)	0.67389(4)	0.00999(12)
C6	0.23736(13)	0.75832(11)	0.71614(3)	0.01064(11)
C7	0.38151(14)	0.89615(11)	0.72695(4)	0.01218(13)
C8	0.55959(14)	0.90533(11)	0.69376(4)	0.01212(13)
C9	0.59604(13)	0.77797(10)	0.65025(4)	0.01015(12)
C10	0.45499(13)	0.63617(10)	0.64077(3)	0.00868(11)
O11	0.37449(11)	0.51673(9)	0.54667(3)	0.01140(10)
C13	0.44911(14)	0.29368(10)	0.61896(4)	0.01060(12)
C14	0.26002(13)	0.24373(11)	0.58492(3)	0.01069(11)
C15	0.23747(13)	0.38006(11)	0.53839(4)	0.01027(12)
O15	0.11876(12)	0.38043(10)	0.49802(3)	0.01471(12)
C16	0.12685(17)	0.10699(14)	0.59132(4)	0.01778(16)
Cl1	0.01627(3)	0.74280(3)	0.75903(2)	0.01565(5)

Table SI08. Hydrogen coordinates and isotropic displacement parameters (Å^2) for *rac*-**10o**-Cry2.

	x	y	z	U_{iso}
H21	0.9335	0.7029	0.5530	0.021(3)
H22	0.6951	0.7494	0.5390	0.021(3)
H31	0.8219	0.4511	0.6096	0.017(3)
H32	0.7523	0.4296	0.5440	0.017(3)
H5	0.1729	0.5372	0.6673	0.017(2)
H7	0.3576	0.9820	0.7566	0.017(2)
H8	0.6582	0.9989	0.7005	0.017(2)
H131	0.5646	0.2082	0.6114	0.022(3)
H132	0.4183	0.2935	0.6606	0.022(3)
H161	0.0129	0.0943	0.5657	0.032(4)
H162	0.1457	0.0222	0.6215	0.032(4)

Table SI09. Anisotropic displacement parameters (Å^2) for *rac*-**10o**-Cry2. The anisotropic displacement factor exponent takes the form: $-2\pi^2[h^2 a^{*2}U_{11} + ... + 2 h k a^* b^* U_{12}]$.

	U_{11}	U_{22}	U_{33}	U_{23}	U_{13}	U_{12}
O1	0.0135(3)	0.0145(2)	0.0160(3)	-0.0027(2)	0.0038(2)	-0.0063(2)
C2	0.0160(4)	0.0155(3)	0.0139(3)	-0.0009(3)	0.0045(3)	-0.0047(3)
C3	0.0118(3)	0.0127(3)	0.0159(4)	-0.0019(3)	0.0029(3)	-0.0012(3)
C4	0.0106(3)	0.0086(2)	0.0082(3)	0.0001(2)	-0.0006(2)	-0.0012(2)
C5	0.0106(3)	0.0086(2)	0.0108(3)	-0.0002(2)	0.0003(2)	-0.0007(2)
C6	0.0115(3)	0.0099(2)	0.0106(3)	0.0002(2)	0.0006(2)	0.0013(2)
C7	0.0148(3)	0.0102(3)	0.0115(3)	-0.0021(2)	-0.0019(2)	0.0013(2)
C8	0.0134(3)	0.0095(3)	0.0135(3)	-0.0018(2)	-0.0022(3)	-0.0019(2)
C9	0.0109(3)	0.0089(3)	0.0107(3)	0.0001(2)	-0.0004(2)	-0.0022(2)
C10	0.0100(3)	0.0077(2)	0.0084(3)	0.0001(2)	-0.0005(2)	-0.0011(2)
O11	0.0153(3)	0.0102(2)	0.0088(2)	0.00088(18)	-0.0026(2)	-0.0028(2)
C13	0.0134(3)	0.0080(2)	0.0104(3)	0.0005(2)	-0.0011(2)	-0.0010(2)
C14	0.0122(3)	0.0096(2)	0.0102(3)	-0.0002(2)	0.0003(2)	-0.0021(3)
C15	0.0113(3)	0.0106(3)	0.0089(3)	-0.0013(2)	0.0004(2)	-0.0010(2)
O15	0.0148(3)	0.0177(3)	0.0116(3)	-0.0008(2)	-0.0038(2)	-0.0012(2)
C16	0.0191(4)	0.0164(3)	0.0179(4)	0.0028(3)	-0.0022(3)	-0.0082(3)
Cl1	0.01536(9)	0.01426(8)	0.01733(9)	-0.00054(6)	0.00593(7)	0.00252(7)

CCDC 1944606 contains the supplementary crystallographic data for this structure. This data can be obtained free of charge via http://www.ccdc.cam.ac.uk/conts/retrieving.html (or from the CCDC, 12 Union Road, Cambridge CB2 1EZ, UK; Fax: +44 1223 336033; E-mail: deposit@ccdc.cam.ac.uk).

Table SI10. Full list of bond lengths [Å] and angles [°] for *rac*-**10o**-Cry2.

O1-C9	1.3589(11)
O1-C2	1.4405(12)
C2-C3	1.5126(13)
C2-H21	0.99
C2-H22	0.99
C3-C4	1.5210(12)
C3-H31	0.99
C3-H32	0.99
C4-O11	1.4769(10)
C4-C10	1.5187(11)
C4-C13	1.5505(11)
C5-C6	1.3852(11)
C5-C10	1.4049(12)
C5-H5	0.95
C6-C7	1.3985(12)
C6-Cl1	1.7417(8)
C7-C8	1.3835(13)
C7-H7	0.95
C8-C9	1.4025(12)
C8-H8	0.95
C9-C10	1.4018(11)
O11-C15	1.3525(10)
C13-C14	1.4979(12)
C13-H131	0.99
C13-H132	0.99
C14-C16	1.3313(12)
C14-C15	1.4866(12)
C15-O15	1.2119(11)
C16-H161	0.95
C16-H162	0.95

C9-O1-C2	114.94(7)
O1-C2-C3	110.06(8)
O1-C2-H21	109.6
C3-C2-H21	109.6
O1-C2-H22	109.6
C3-C2-H22	109.6
H21-C2-H22	108.2
C2-C3-C4	110.78(7)
C2-C3-H31	109.5
C4-C3-H31	109.5
C2-C3-H32	109.5
C4-C3-H32	109.5
H31-C3-H32	108.1
O11-C4-C10	107.81(6)
O11-C4-C3	107.42(6)
C10-C4-C3	109.82(7)
O11-C4-C13	105.11(6)
C10-C4-C13	114.22(6)
C3-C4-C13	112.06(7)
C6-C5-C10	120.34(7)
C6-C5-H5	119.8
C10-C5-H5	119.8
C5-C6-C7	121.13(8)
C5-C6-Cl1	119.79(6)
C7-C6-Cl1	119.01(6)
C8-C7-C6	118.88(8)
C8-C7-H7	120.6
C6-C7-H7	120.6
C7-C8-C9	120.68(8)
C7-C8-H8	119.7
C9-C8-H8	119.7
O1-C9-C10	124.04(7)
O1-C9-C8	115.54(7)
C10-C9-C8	120.39(8)
C9-C10-C5	118.52(7)
C9-C10-C4	120.49(7)
C5-C10-C4	120.90(7)
C15-O11-C4	112.03(6)
C14-C13-C4	104.27(7)
C14-C13-H131	110.9
C4-C13-H131	110.9
C14-C13-H132	110.9
C4-C13-H132	110.9
H131-C13-H132	108.9
C16-C14-C15	122.12(8)
C16-C14-C13	130.45(8)
C15-C14-C13	107.43(7)
O15-C15-O11	121.29(8)
O15-C15-C14	129.05(8)
O11-C15-C14	109.66(7)
C14-C16-H161	120.0
C14-C16-H162	120.0
H161-C16-H162	120.0

C9-O1-C2-C3	-48.84(11)
O1-C2-C3-C4	63.90(10)
C2-C3-C4-O11	74.10(9)
C2-C3-C4-C10	-42.89(10)
C2-C3-C4-C13	-170.95(7)
C10-C5-C6-C7	-0.15(12)
C10-C5-C6-Cl1	-177.07(6)
C5-C6-C7-C8	1.42(13)
Cl1-C6-C7-C8	178.36(7)
C6-C7-C8-C9	-0.46(13)
C2-O1-C9-C10	15.46(12)
C2-O1-C9-C8	-166.38(8)
C7-C8-C9-O1	180.00(8)
C7-C8-C9-C10	-1.76(13)
O1-C9-C10-C5	-178.94(8)
C8-C9-C10-C5	2.99(12)
O1-C9-C10-C4	4.47(12)
C8-C9-C10-C4	-173.61(8)
C6-C5-C10-C9	-2.04(12)
C6-C5-C10-C4	174.54(7)
O11-C4-C10-C9	-106.23(8)
C3-C4-C10-C9	10.51(10)
C13-C4-C10-C9	137.37(8)
O11-C4-C10-C5	77.26(9)
C3-C4-C10-C5	-166.00(7)
C13-C4-C10-C5	-39.14(11)
C10-C4-O11-C15	-115.60(7)
C3-C4-O11-C15	126.11(7)
C13-C4-O11-C15	6.61(9)
O11-C4-C13-C14	-11.25(8)
C10-C4-C13-C14	106.71(8)
C3-C4-C13-C14	-127.61(7)
C4-C13-C14-C16	-167.92(10)
C4-C13-C14-C15	12.02(9)
C4-O11-C15-O15	-179.38(8)
C4-O11-C15-C14	1.04(9)
C16-C14-C15-O15	-8.20(15)
C13-C14-C15-O15	171.85(9)
C16-C14-C15-O11	171.35(9)
C13-C14-C15-O11	-8.60(9)

(*R*)-10o

Crystal Structure Determination of (*R*)-10o. All the measurements were performed using monochromatized Mo K_α radiation at 100K: $C_{13}H_{11}ClO_3$, M_r 250.67, orthorhombic, space group P 2_1 2_1 2_1, a = 6.4226(3)Å, b = 7.3771(4)Å, c = 23.2909(11)Å, V = 1103.53(9)Å^3, Z = 4, d_{calc} = 1.509g cm^{-3}, μ = 0.338mm^{-1}. A total of 23480 reflections were collected (Θ_{max} = 39.9°), from which 6832 were unique (R_{int} = 0.0257), with 6562 having I > 2σ(I). The structure was solved by direct methods (SHELXS-97)[7] and refined by full-matrix least-squares techniques against F^2 (SHELXL-2014/6)[8]. The non-hydrogen atoms were refined with anisotropic displacement parameters without any constraints. The absolute configuration was established by anomalous dispersion effects in the diffraction measurements on the crystal. The H atoms of the terminal CH_2 group were refined with a common isotropic displacement parameter and idealized geometry with the hydrogen atoms in the plane through the atoms C13, C15, C16 and C–H distances of 0.95Å. The H atoms of the other CH_2 groups were refined with common isotropic displacement parameters for the H atoms of the same group and idealized geometry with approximately tetrahedral angles and C–H distances of 0.99Å. The H atoms of the phenyl ring were put at the external bisectors of the C–C–C angles at C–H distances of 0.95Å and a common isotropic displacement parameter was refined for these H atoms. For 159 parameters final *R* indices of R1 = 0.0267 and wR^2 = 0.0732 (GOF = 1.087) were obtained. The largest peak in a difference Fourier map was 0.512eÅ^{-3}.

Results and Discussion

Crystal Structure. The crystal structure analysis of (*R*)-**10o** confirmed the compound as (4*R*)-6-chloro-4'-methylene-2,3,3',4'-tetrahydro-5'*H*-spiro[chromene-4,2'-furan]-5'-one. All atoms lie on general positions (see Fig. SI02). The determination of the absolute configuration from anomalous dispersion effects resulted in a Flack-parameter[9] of -0.02(4).

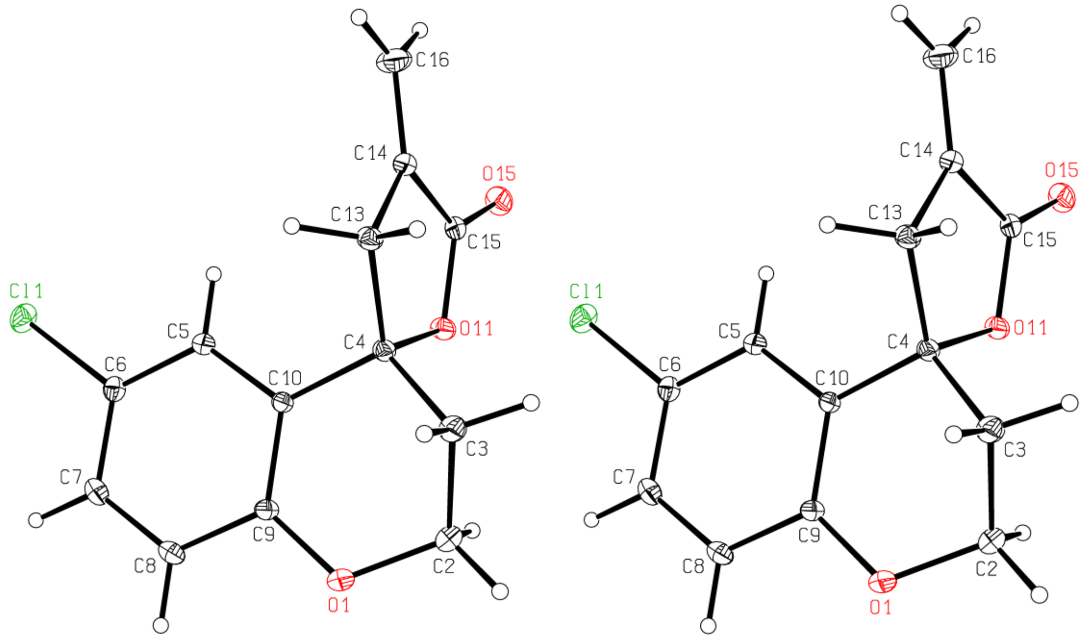

Figure SI02. Stereoscopic ORTEP[10] plot of (*R*)-**10o** showing the atomic numbering scheme. The probability ellipsoids are drawn at the 50% probability level. The H atoms are drawn with arbitrary radii.

Table SI11. Crystal data and structure refinement for (*R*)-**10**.

Crystal data	
CCDC number	1944607
Identification code	FE122
Empirical formula	$C_{13}H_{11}ClO_3$
Formula weight	250.67
Crystal description	needle, colourless
Crystal size	0.45 x 0.38 x 0.30mm
Crystal system, space group	orthorhombic, P 2_1 2_1 2_1
Unit cell dimensions: a	6.4226(3)Å
b	7.3771(4)Å
c	23.2909(11)Å
Volume	1103.53(9)$Å^3$
Z	4
Calculated density	1.509Mg/m^3
F(000)	520
Linear absorption coefficient μ	0.338mm^{-1}
Absorption correction	semi-empirical from equivalents
Max. and min. transmission	1.000 and 0.908
Unit cell determination	2.76° < Θ < 40.69°
	9930 reflections used at 100K
Data collection	
Temperature	100K
Diffractometer	Bruker APEX-II CCD
Radiation source	Incoatec microfocus sealed tube
Radiation and wavelength	MoK_α, 0.71073Å
Monochromator	multilayer monochromator
Scan type	ϕ and ω scans
Θ range for data collection	2.90 to 40.00°
Reflections collected / unique	23480 / 6832
Significant unique reflections	6562 with I > 2σ(I)
R(int), R(sigma)	0.0257, 0.0240
Completeness to Θ = 39.99°	99.9%
Refinement	
Refinement method	Full-matrix least-squares on F^2
Data / parameters / restraints	6832 / 159 / 0
Goodness-of-fit on F^2	1.087
Final R indices [I > 2σ(I)]	R1 = 0.0267, wR2 = 0.0722
R indices (all data)	R1 = 0.0284, wR2 = 0.0732
Absolute structure parameter	-0.016(35)
Extinction expression	none
Weighting scheme	$w = 1/[\sigma^2(F_o^2)+(aP)^2+bP]$ where $P = (F_o^2+2F_c^2)/3$
Weighting scheme parameters a, b	0.0432, 0.0676
Largest Δ/σ in last cycle	0.001
Largest difference peak and hole	0.512 and -0.250e/$Å^3$
Structure Solution Program	SHELXS-97 (Sheldrick, 2008)
Structure Refinement Program	SHELXL-2014/6 (Sheldrick, 2015)

Table SI12. Atomic coordinates and equivalent isotropic displacement parameters (Å^2) for (*R*)-**10**. U_{eq} is defined as one third of the trace of the orthogonalized U_{ij} tensor.

	x	y	z	U_{eq}
O1	0.22403(11)	0.19968(10)	0.37960(3)	0.01445(12)
C2	0.21011(15)	0.30322(13)	0.43186(4)	0.01494(14)
C3	0.26895(14)	0.49832(12)	0.42071(4)	0.01304(13)
C4	0.49509(12)	0.51281(10)	0.40200(3)	0.00888(11)
C5	0.72720(13)	0.37007(11)	0.32608(3)	0.00967(11)
C6	0.76244(12)	0.24180(11)	0.28381(3)	0.01024(11)
C7	0.61847(14)	0.10377(11)	0.27306(4)	0.01183(12)
C8	0.44032(14)	0.09464(11)	0.30627(4)	0.01180(13)
C9	0.40396(12)	0.22195(10)	0.34975(3)	0.00980(12)
C10	0.54470(12)	0.36401(10)	0.35923(3)	0.00842(11)
O11	0.62529(10)	0.48330(9)	0.45330(3)	0.01104(10)
C13	0.55066(13)	0.70619(11)	0.38100(4)	0.01036(12)
C14	0.73974(12)	0.75633(12)	0.41506(3)	0.01031(11)
C15	0.76236(12)	0.61997(11)	0.46158(3)	0.00995(12)
O15	0.88102(11)	0.61967(10)	0.50198(3)	0.01440(12)
C16	0.87276(16)	0.89311(14)	0.40864(4)	0.01738(16)
Cl1	0.98367(3)	0.25725(3)	0.24096(2)	0.01528(5)

Table SI13. Hydrogen coordinates and isotropic displacement parameters (Å^2) for (*R*)-**10**.

	x	y	z	U_{iso}
H21	0.3047	0.2509	0.4611	0.020(3)
H22	0.0662	0.2974	0.4470	0.020(3)
H31	0.2476	0.5703	0.4561	0.016(3)
H32	0.1779	0.5488	0.3904	0.016(3)
H5	0.8270	0.4627	0.3327	0.019(2)
H7	0.6426	0.0177	0.2434	0.019(2)
H8	0.3416	0.0011	0.2995	0.019(2)
H131	0.5815	0.7064	0.3394	0.025(3)
H132	0.4350	0.7916	0.3886	0.025(3)
H161	0.9868	0.9059	0.4343	0.031(3)
H162	0.8537	0.9779	0.3784	0.031(3)

Table SI14. Anisotropic displacement parameters (Å^2) for (*R*)-**10**. The anisotropic displacement factor exponent takes the form: $-2\pi^2[h^2 a^{*2}U_{11} + ... + 2 h k a^* b^* U_{12}]$.

	U_{11}	U_{22}	U_{33}	U_{23}	U_{13}	U_{12}
O1	0.0131(3)	0.0150(3)	0.0152(3)	-0.0028(2)	0.0038(2)	-0.0062(2)
C2	0.0159(3)	0.0157(3)	0.0132(3)	-0.0008(3)	0.0044(3)	-0.0050(3)
C3	0.0113(3)	0.0129(3)	0.0149(3)	-0.0020(3)	0.0029(2)	-0.0013(2)
C4	0.0100(3)	0.0087(2)	0.0079(2)	0.0000(2)	-0.0009(2)	-0.0011(2)
C5	0.0101(3)	0.0089(3)	0.0100(3)	-0.0004(2)	0.0002(2)	-0.0009(2)
C6	0.0108(3)	0.0103(3)	0.0096(3)	0.0002(2)	0.0005(2)	0.0018(2)
C7	0.0142(3)	0.0106(3)	0.0107(3)	-0.0021(2)	-0.0018(2)	0.0013(2)
C8	0.0130(3)	0.0099(3)	0.0125(3)	-0.0020(2)	-0.0019(2)	-0.0018(2)
C9	0.0103(3)	0.0091(3)	0.0100(3)	0.0000(2)	-0.0001(2)	-0.0023(2)
C10	0.0097(3)	0.0078(2)	0.0078(2)	0.0000(2)	-0.0004(2)	-0.0011(2)
O11	0.0149(3)	0.0103(2)	0.0079(2)	0.00115(18)	-0.00256(19)	-0.00295(19)
C13	0.0128(3)	0.0083(3)	0.0100(3)	0.0005(2)	-0.0013(2)	-0.0007(2)
C14	0.0118(3)	0.0097(3)	0.0094(3)	-0.0006(2)	0.0005(2)	-0.0020(2)
C15	0.0109(3)	0.0106(3)	0.0083(3)	-0.0013(2)	0.0004(2)	-0.0008(2)
O15	0.0142(3)	0.0181(3)	0.0108(2)	-0.0011(2)	-0.0040(2)	-0.0011(2)
C16	0.0188(4)	0.0162(4)	0.0171(4)	0.0031(3)	-0.0023(3)	-0.0080(3)
Cl1	0.01476(8)	0.01454(8)	0.01653(9)	-0.00050(6)	0.00601(6)	0.00252(7)

CCDC 1944607 contains the supplementary crystallographic data for this structure. This data can be obtained free of charge via http://www.ccdc.cam.ac.uk/conts/retrieving.html (or from the CCDC, 12 Union Road, Cambridge CB2 1EZ, UK; Fax: +44 1223 336033; E-mail: deposit@ccdc.cam.ac.uk).

Table SI15. Full list of bond lengths [Å] and angles [°] for (*R*)-**10**.

O1-C9	1.3587(10)
O1-C2	1.4397(11)
C2-C3	1.5105(13)
C2-H21	0.99
C2-H22	0.99
C3-C4	1.5202(12)
C3-H31	0.99
C3-H32	0.99
C4-O11	1.4746(10)
C4-C10	1.5161(10)
C4-C13	1.5497(11)
C5-C6	1.3841(11)
C5-C10	1.4042(11)
C5-H5	0.95
C6-C7	1.3981(12)
C6-Cl1	1.7401(8)
C7-C8	1.3828(13)
C7-H7	0.95
C8-C9	1.4007(12)
C8-H8	0.95
C9-C10	1.4015(11)
O11-C15	1.3523(10)
C13-C14	1.4969(11)
C13-H131	0.99
C13-H132	0.99
C14-C16	1.3305(12)
C14-C15	1.4856(12)
C15-O15	1.2109(10)
C16-H161	0.95
C16-H162	0.95

C9-O1-C2	114.92(7)
O1-C2-C3	110.16(7)
O1-C2-H21	109.6
C3-C2-H21	109.6
O1-C2-H22	109.6
C3-C2-H22	109.6
H21-C2-H22	108.1
C2-C3-C4	110.82(7)
C2-C3-H31	109.5
C4-C3-H31	109.5
C2-C3-H32	109.5
C4-C3-H32	109.5
H31-C3-H32	108.1
O11-C4-C10	107.84(6)
O11-C4-C3	107.40(6)
C10-C4-C3	109.77(6)
O11-C4-C13	105.13(6)
C10-C4-C13	114.26(6)
C3-C4-C13	112.04(7)
C6-C5-C10	120.38(7)
C6-C5-H5	119.8
C10-C5-H5	119.8
C5-C6-C7	121.15(7)
C5-C6-Cl1	119.78(6)
C7-C6-Cl1	119.01(6)
C8-C7-C6	118.85(8)
C8-C7-H7	120.6
C6-C7-H7	120.6
C7-C8-C9	120.65(8)
C7-C8-H8	119.7
C9-C8-H8	119.7
O1-C9-C8	115.51(7)
O1-C9-C10	123.94(7)
C8-C9-C10	120.52(7)
C9-C10-C5	118.39(7)
C9-C10-C4	120.62(7)
C5-C10-C4	120.90(6)
C15-O11-C4	112.01(6)
C14-C13-C4	104.31(6)
C14-C13-H131	110.9
C4-C13-H131	110.9
C14-C13-H132	110.9
C4-C13-H132	110.9
H131-C13-H132	108.9
C16-C14-C15	122.18(8)
C16-C14-C13	130.45(8)
C15-C14-C13	107.36(7)
O15-C15-O11	121.28(8)
O15-C15-C14	129.02(8)
O11-C15-C14	109.70(7)
C14-C16-H161	120.0
C14-C16-H162	120.0
H161-C16-H162	120.0

C9-O1-C2-C3	48.76(11)
O1-C2-C3-C4	-63.83(10)
C2-C3-C4-O11	-74.10(8)
C2-C3-C4-C10	42.88(9)
C2-C3-C4-C13	170.94(7)
C10-C5-C6-C7	-0.01(12)
C10-C5-C6-Cl1	177.10(6)
C5-C6-C7-C8	-1.27(12)
Cl1-C6-C7-C8	-178.41(7)
C6-C7-C8-C9	0.34(13)
C2-O1-C9-C8	166.47(8)
C2-O1-C9-C10	-15.46(12)
C7-C8-C9-O1	-179.99(8)
C7-C8-C9-C10	1.87(12)
O1-C9-C10-C5	178.92(8)
C8-C9-C10-C5	-3.10(11)
O1-C9-C10-C4	-4.32(12)
C8-C9-C10-C4	173.65(7)
C6-C5-C10-C9	2.18(12)
C6-C5-C10-C4	-174.56(7)
O11-C4-C10-C9	106.09(8)
C3-C4-C10-C9	-10.62(10)
C13-C4-C10-C9	-137.45(7)
O11-C4-C10-C5	-77.24(9)
C3-C4-C10-C5	166.05(7)
C13-C4-C10-C5	39.23(10)
C10-C4-O11-C15	115.64(7)
C3-C4-O11-C15	-126.11(7)
C13-C4-O11-C15	-6.64(8)
O11-C4-C13-C14	11.24(8)
C10-C4-C13-C14	-106.78(7)
C3-C4-C13-C14	127.58(7)
C4-C13-C14-C16	167.94(10)
C4-C13-C14-C15	-11.97(8)
C4-O11-C15-O15	179.35(8)
C4-O11-C15-C14	-0.98(9)
C16-C14-C15-O15	8.25(15)
C13-C14-C15-O15	-171.83(9)
C16-C14-C15-O11	-171.38(9)
C13-C14-C15-O11	8.54(9)

NMR data

Benzyl acrylate (**SI01**) in $CDCl_3$ @ 300.13 MHz (^1H) and 75. 47 MHz (^{13}C).

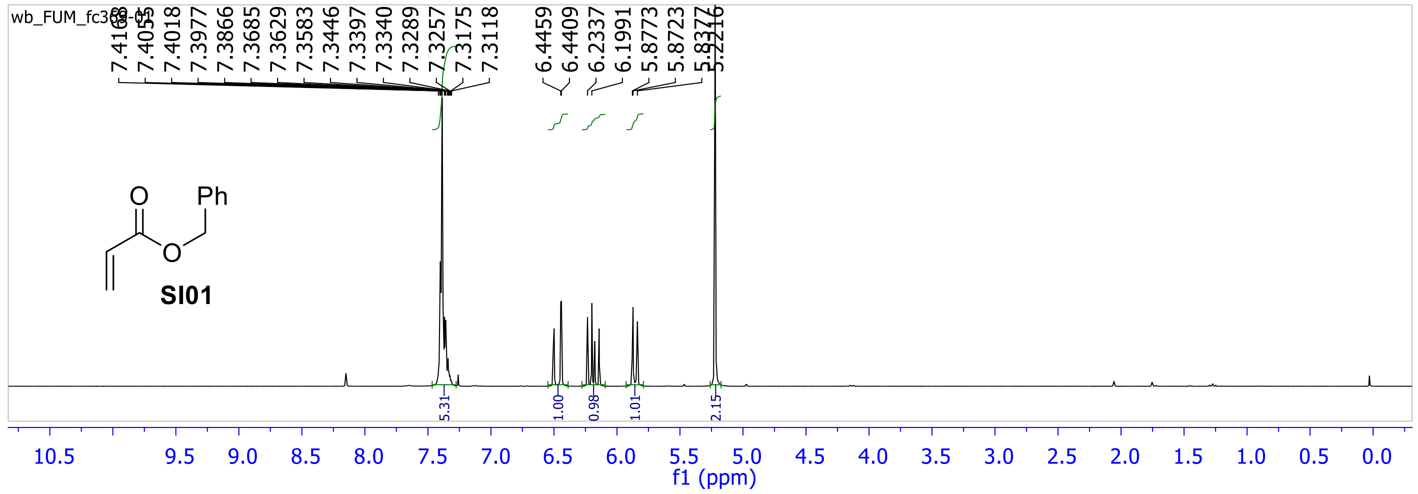

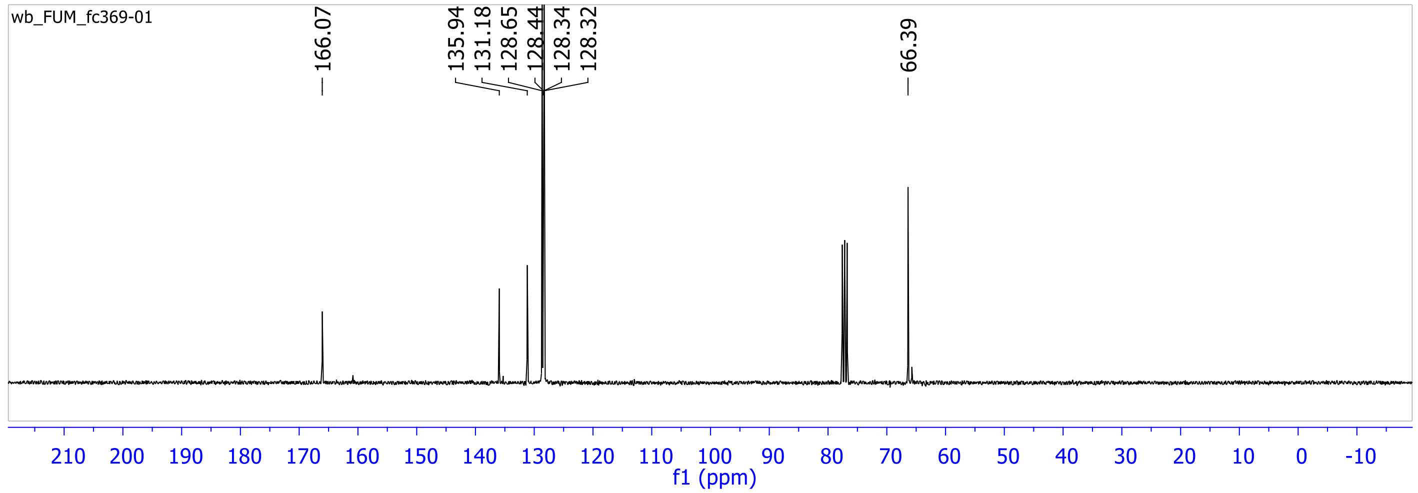

Benzhydryl 2-(hydroxymethyl)acrylate (**SI03**) in $CDCl_3$ @ 300.13 MHz (^{1}H) and 75. 47 MHz (^{13}C).

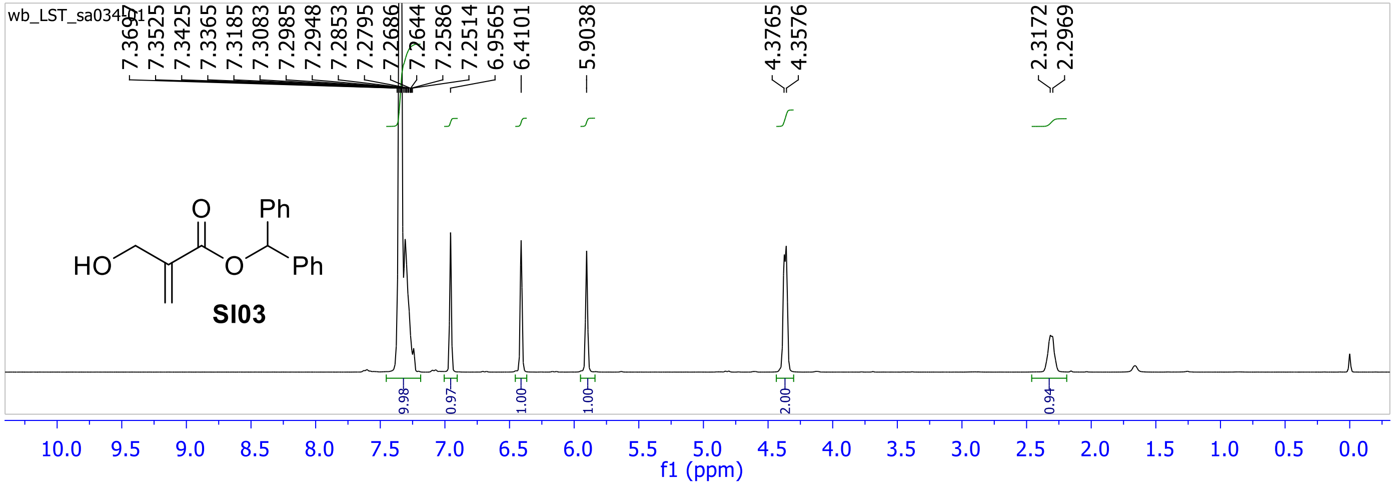

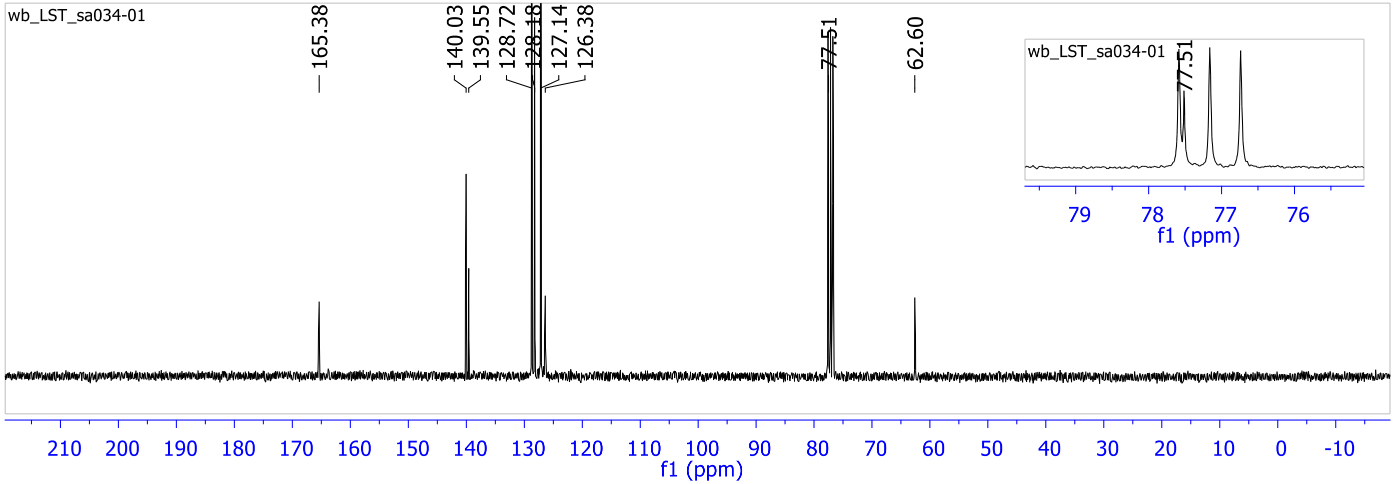

Benzhydryl 2-(bromomethyl)acrylate (**7d**) in $CDCl_3$ @ 300.13 MHz (^1H) and 75. 47 MHz (^{13}C).

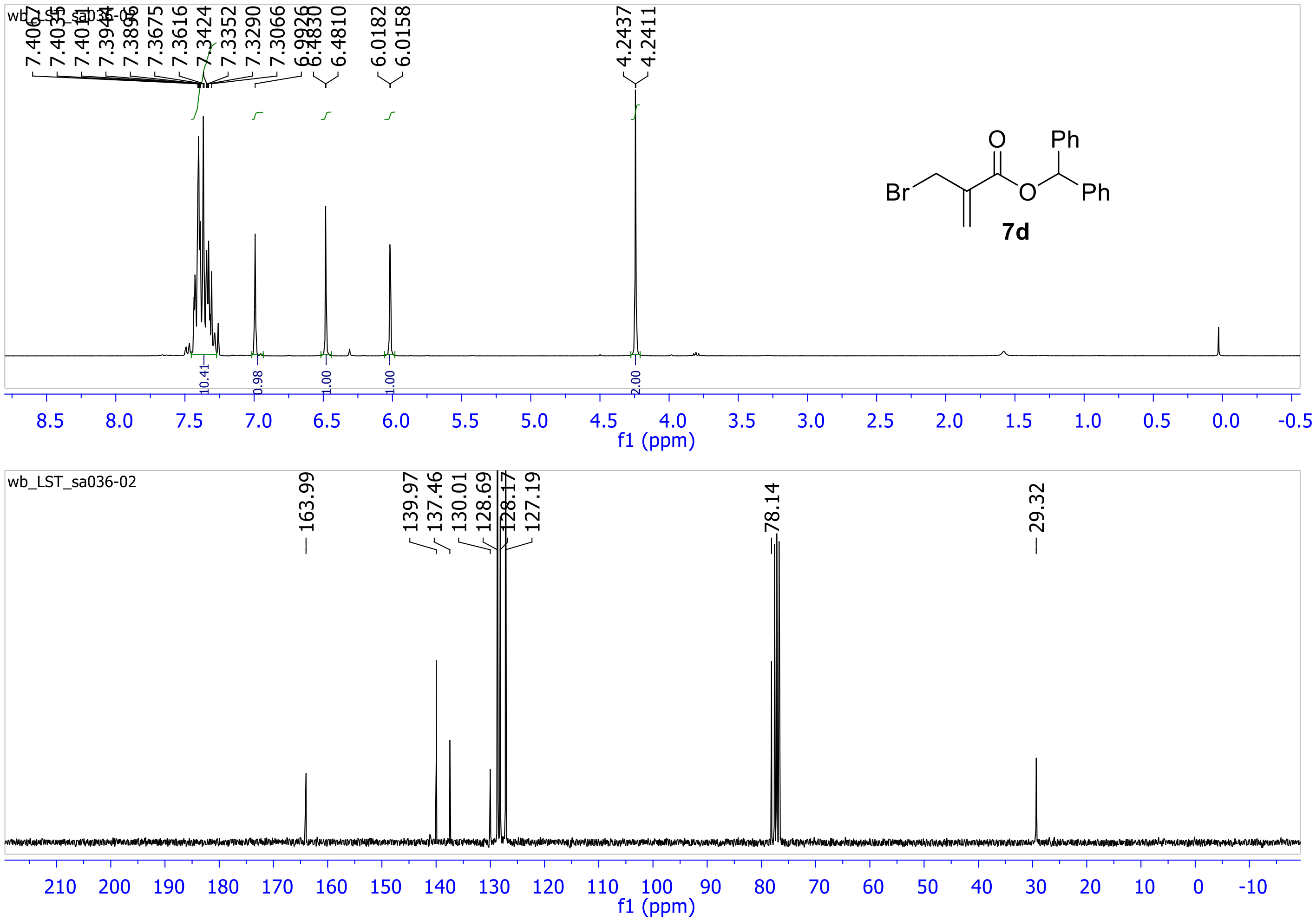

Benzhydryl 3-hydroxy-2-methylenebutanoate (**SI04**) in $CDCl_3$ @ 300.13 MHz (^1H) and 75.47 MHz (^{13}C).

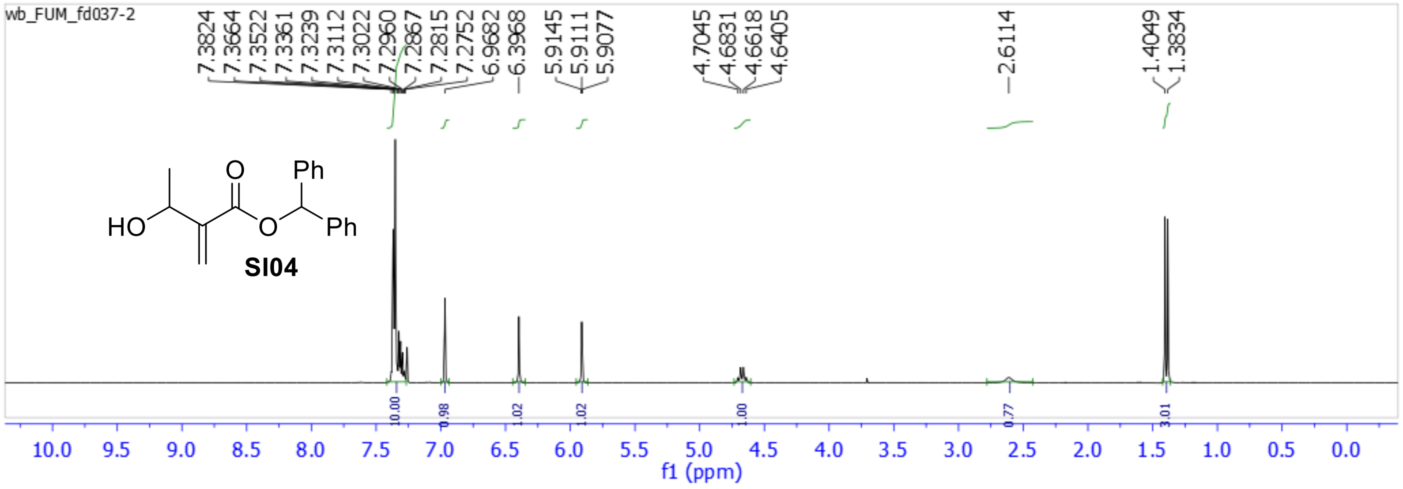

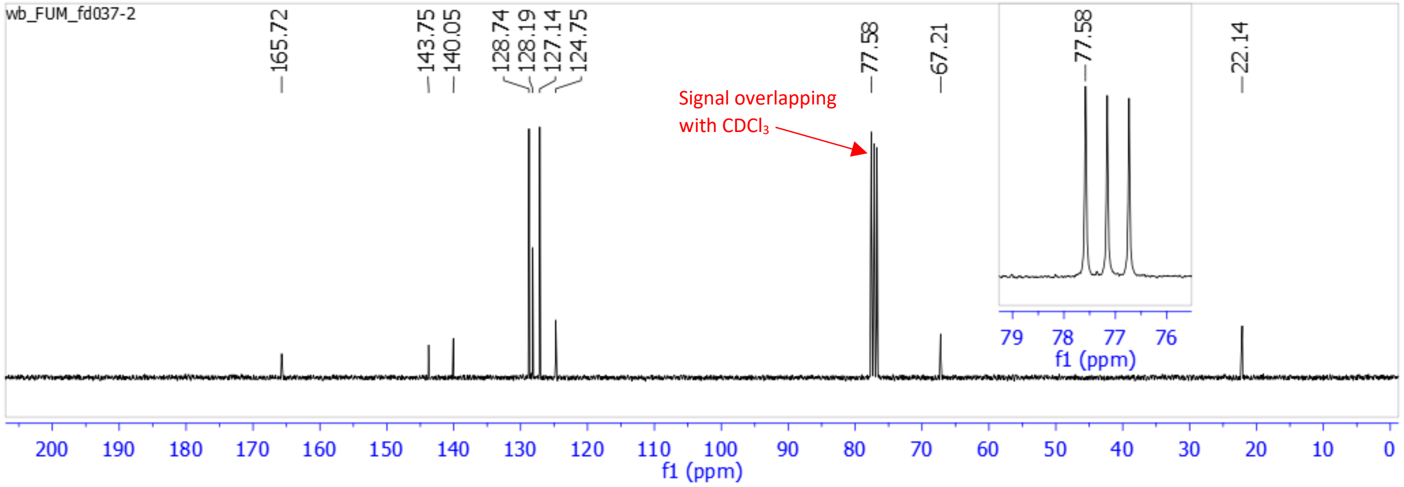

Benzhydryl (Z)-2-(bromomethyl)but-2-enoate (**7e**) in $CDCl_3$ @ 300.13 MHz (^{1}H) and 75. 47 MHz (^{13}C).

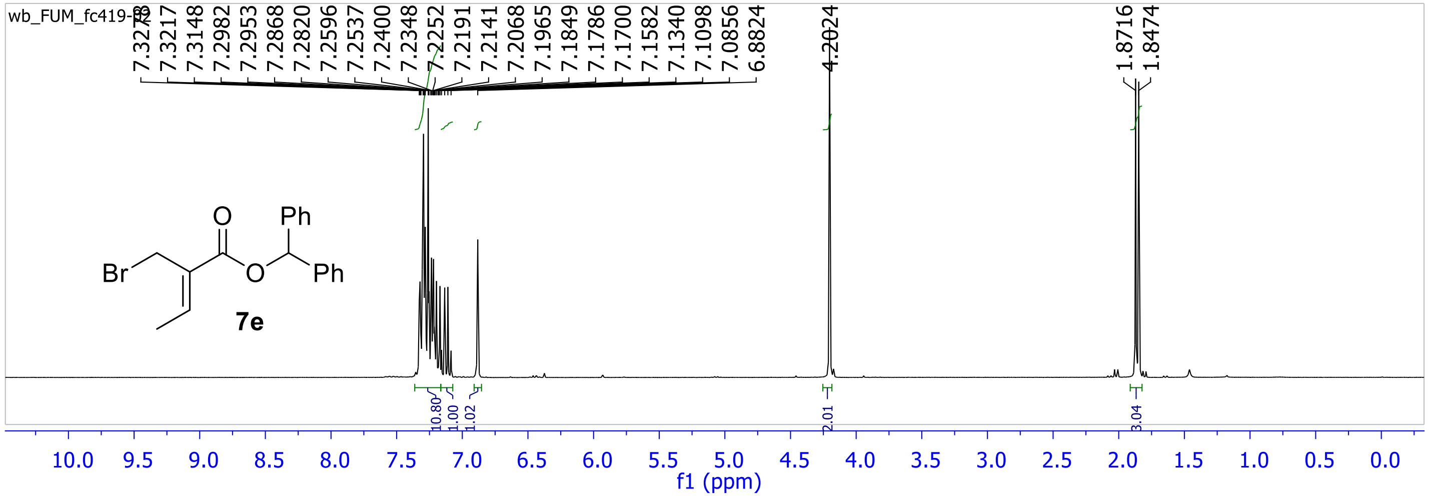

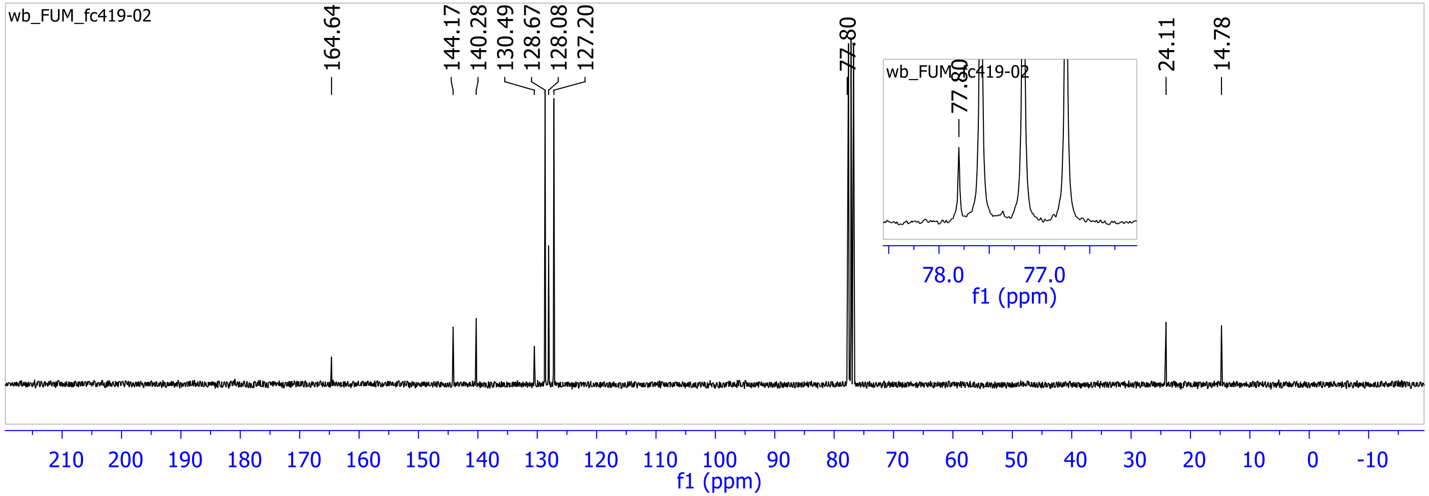

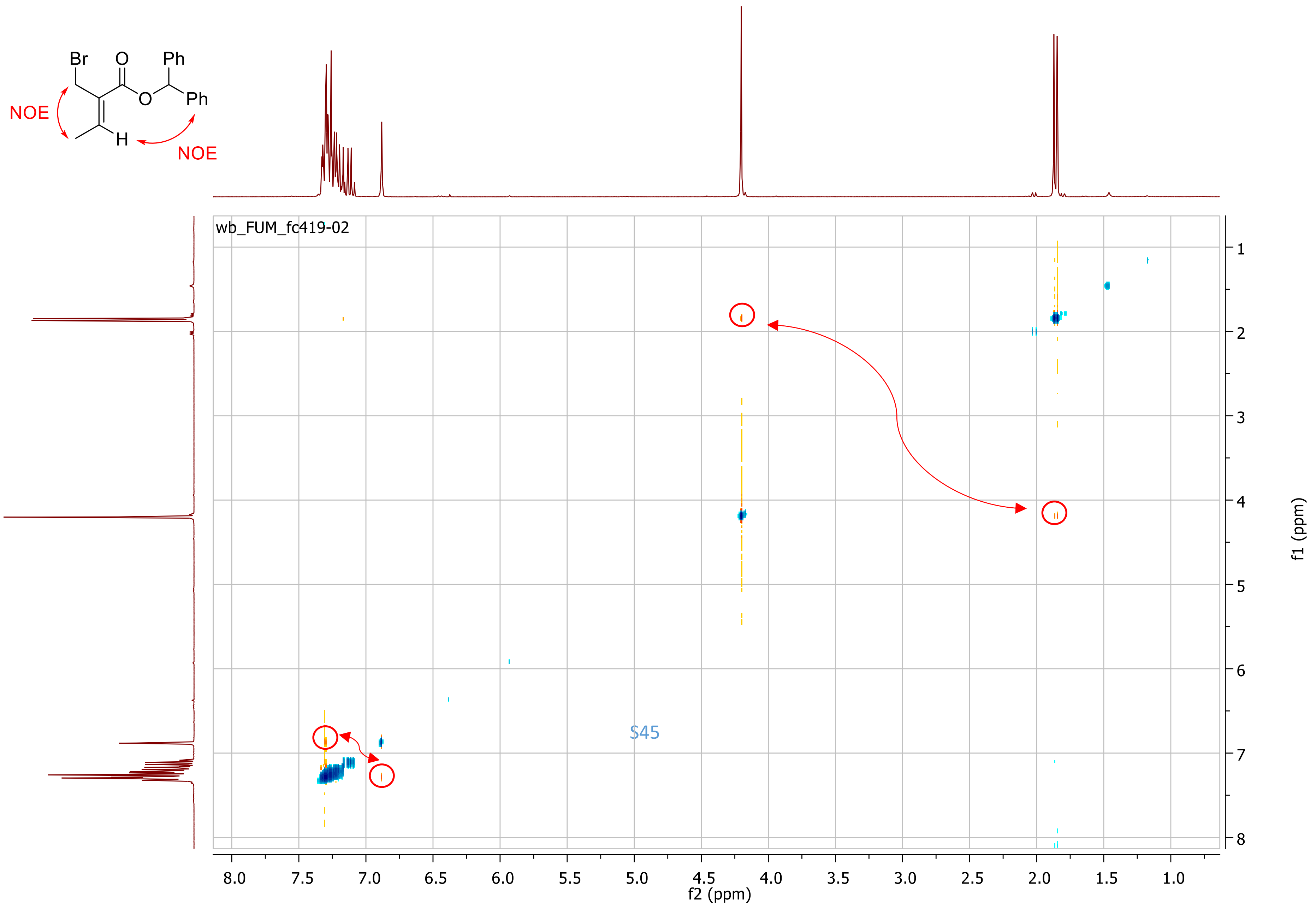

S45

N,*N*-diethyl-2-(hydroxymethyl)acrylamide (SI7).

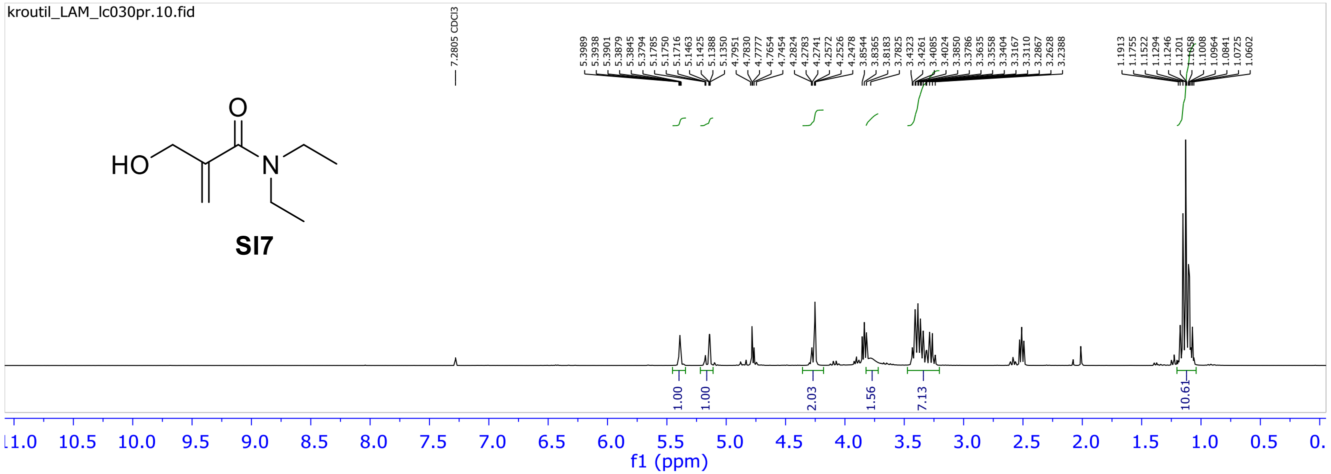

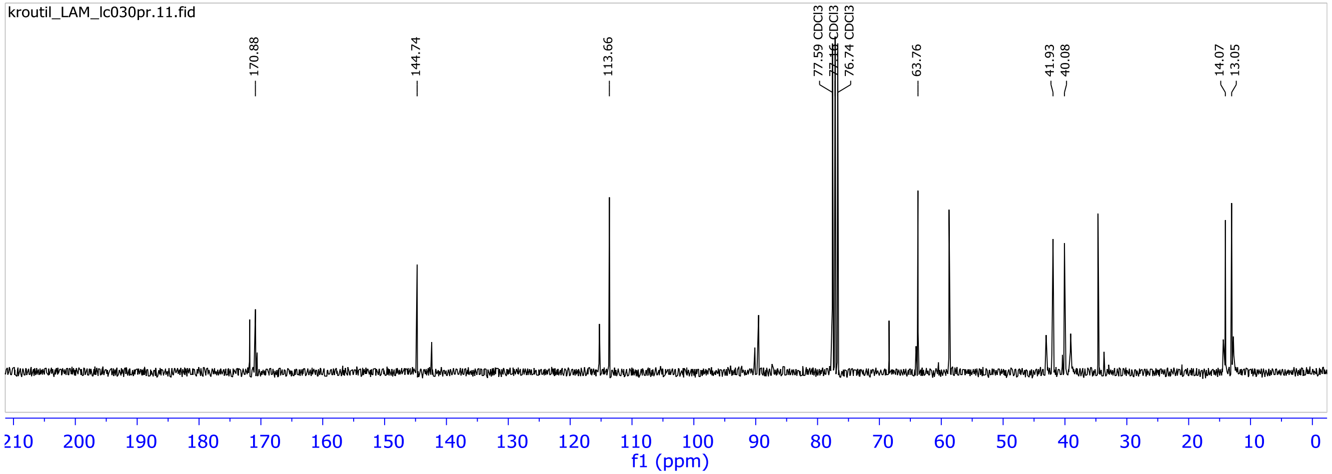

N,*N*-dibenzyl-2-(hydroxymethyl)acrylamide (**SI8**).

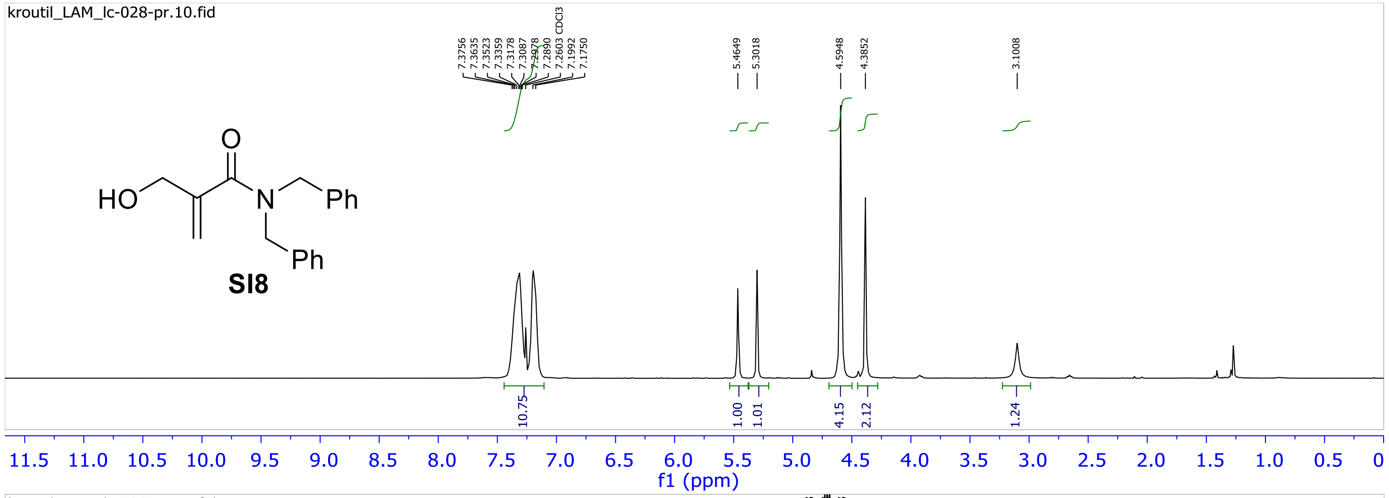

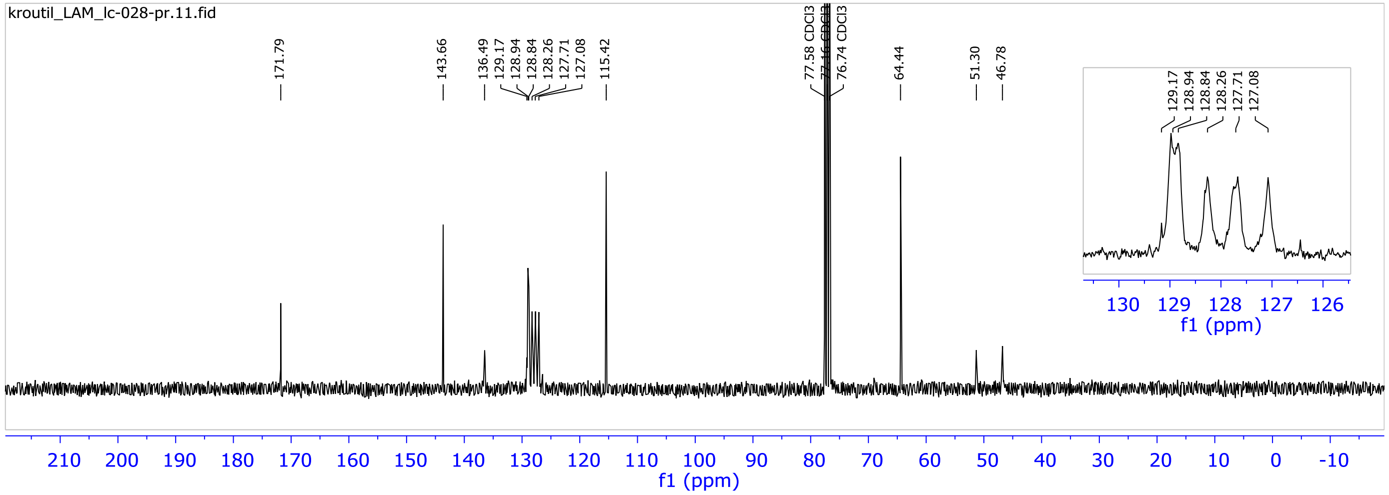

N,*N*-diethyl-2-(bromomethyl)acrylamide (**SI9**).

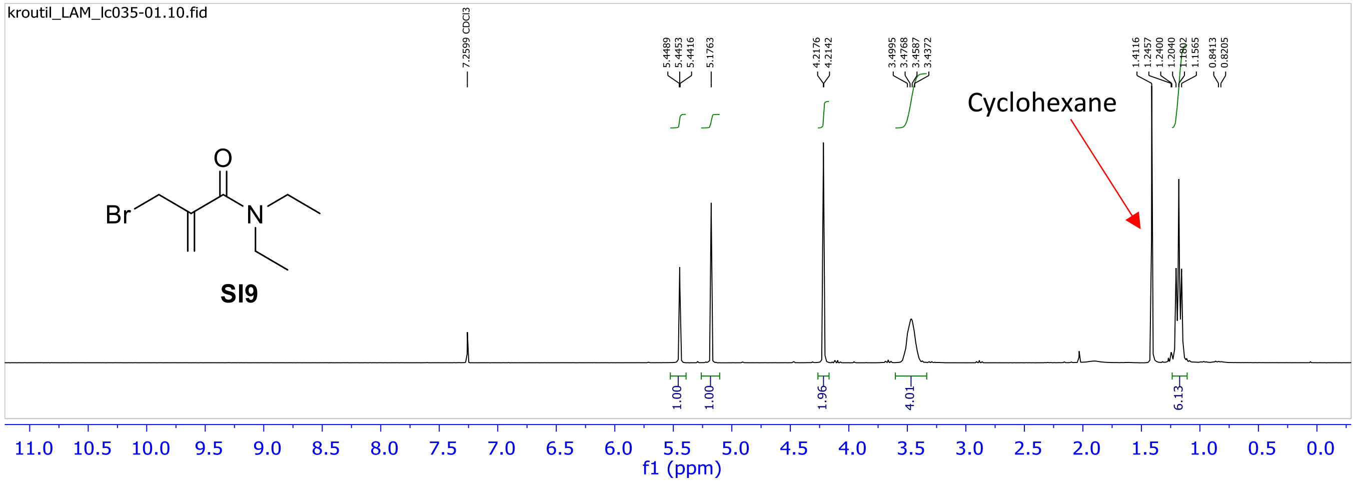

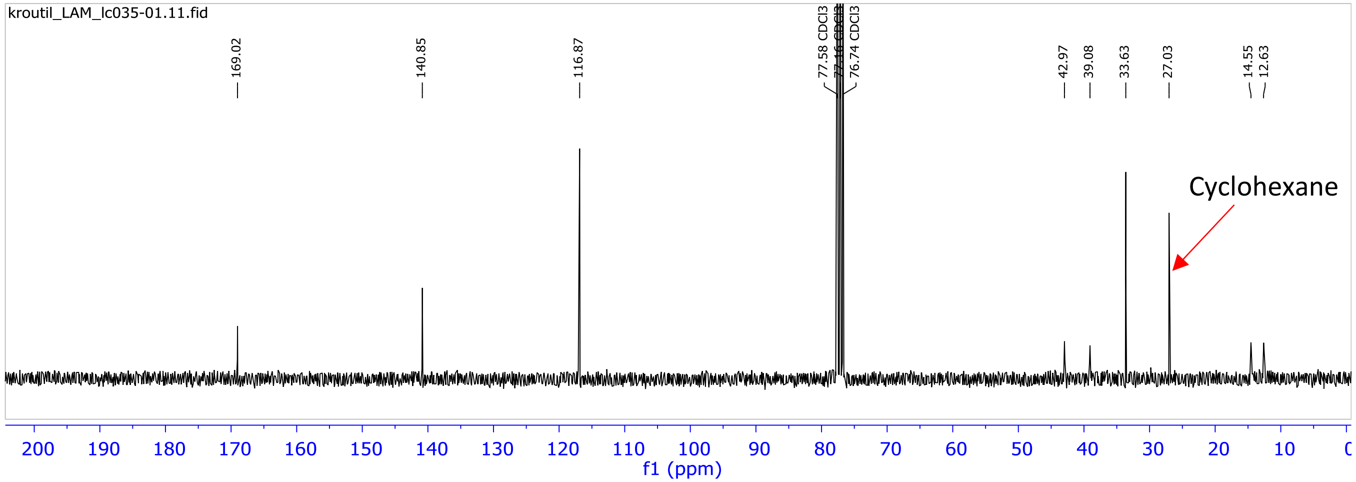

N,*N*-dibenzyl-2-(bromomethyl)acrylamide (**7f**).

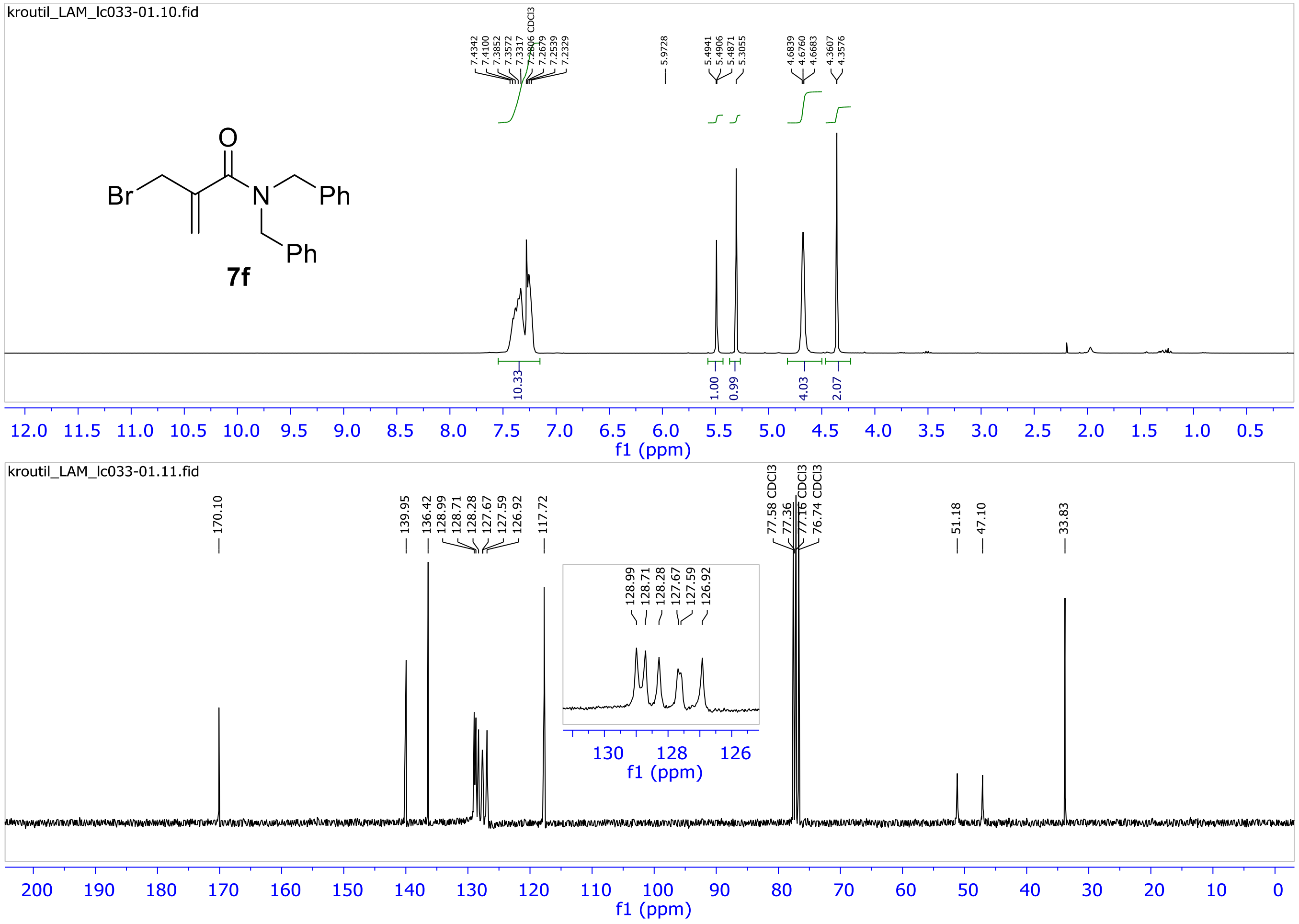

1-Phenylbut-3-en-1-ol (**8a**) in $CDCl_3$ @ 300.13 MHz (^1H) and 75. 47 MHz (^{13}C).

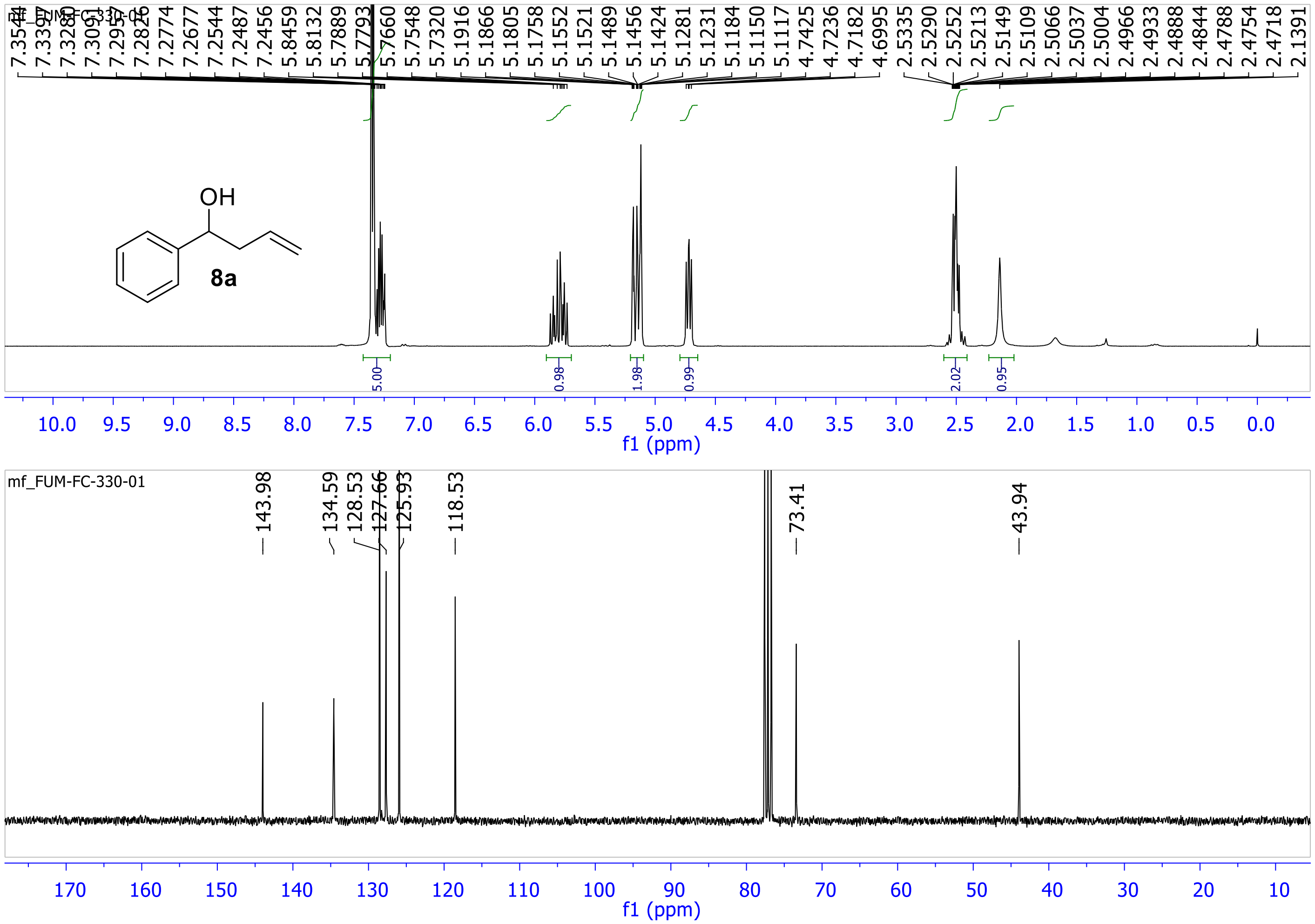

3-Methylene-5-phenyldihydrofuran-2(3*H*)-one (**10a**) in $CDCl_3$ @ 300.13 MHz (^1H) and 75. 47 MHz (^{13}C).

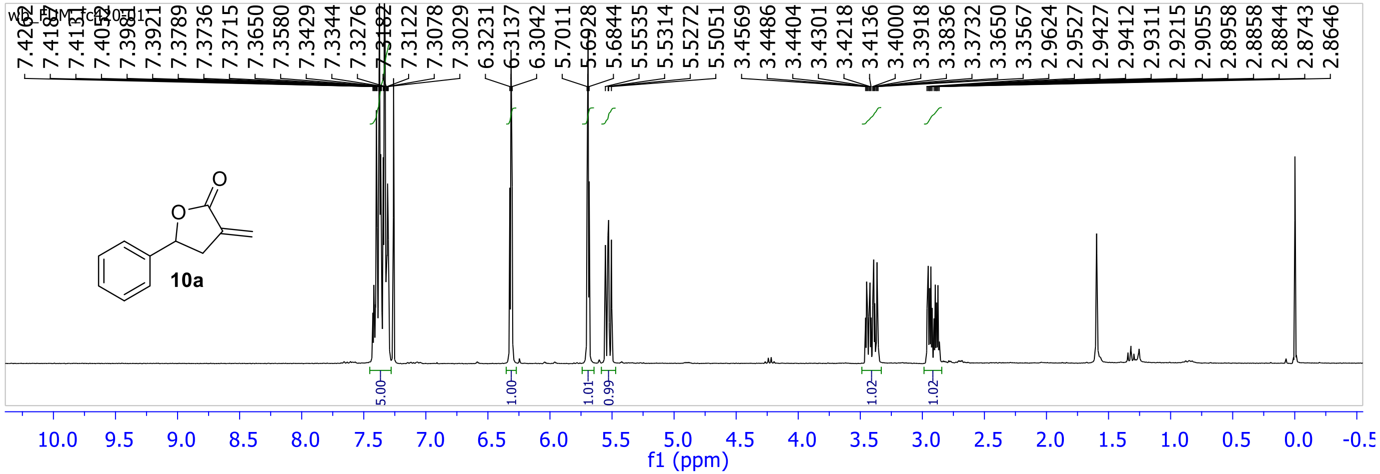

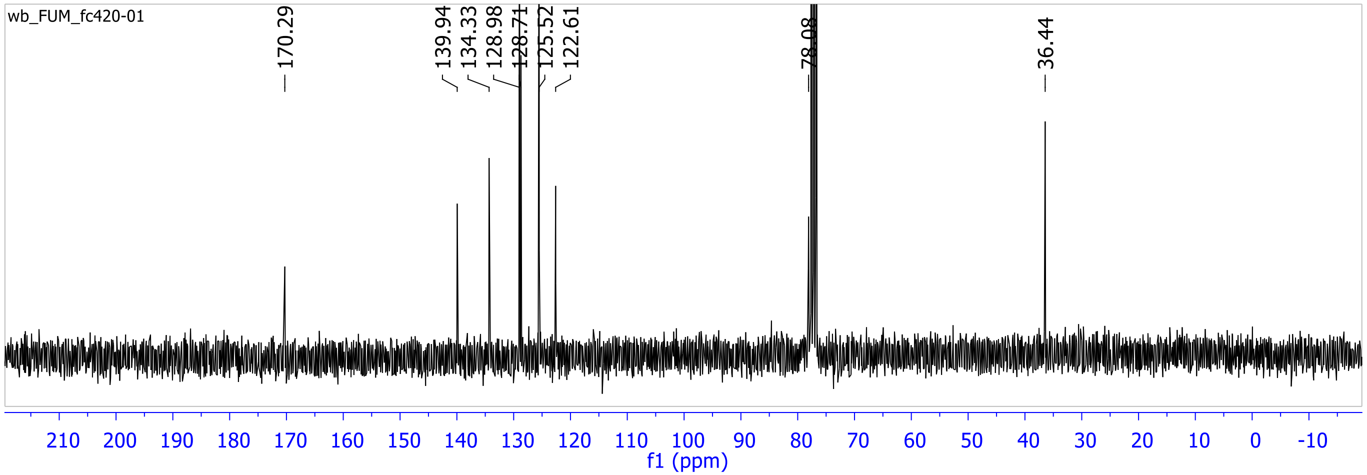

5-Methyl-3-methylene-5-phenyldihydrofuran-2(3*H*)-one (**10b**) in $CDCl_3$ @ 300.13 MHz (^{1}H) and 75. 47 MHz (^{13}C).

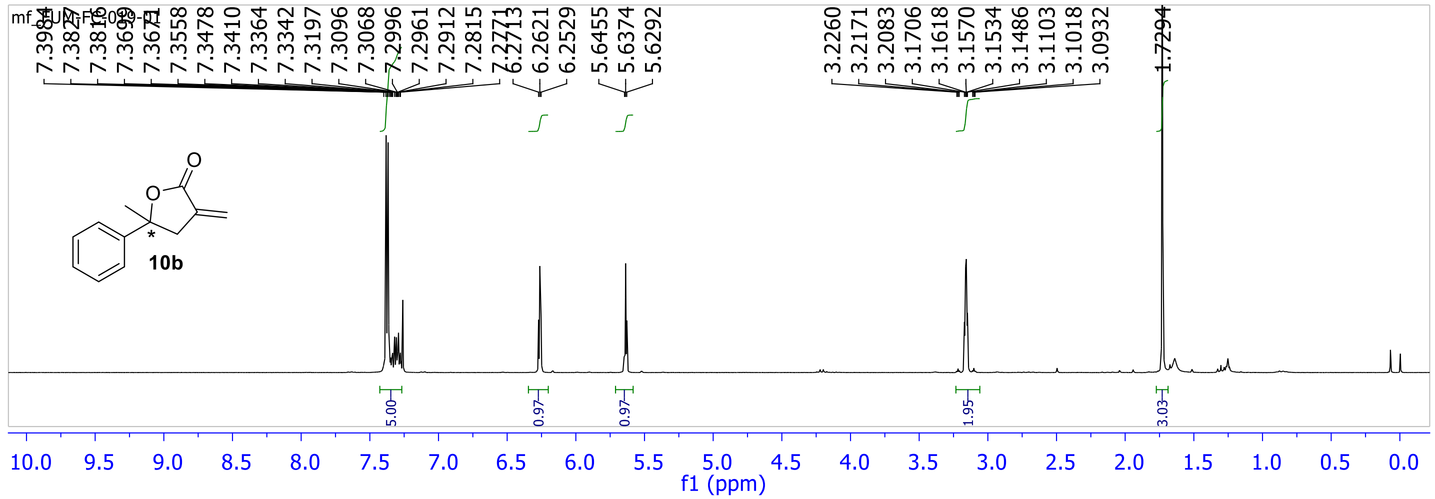

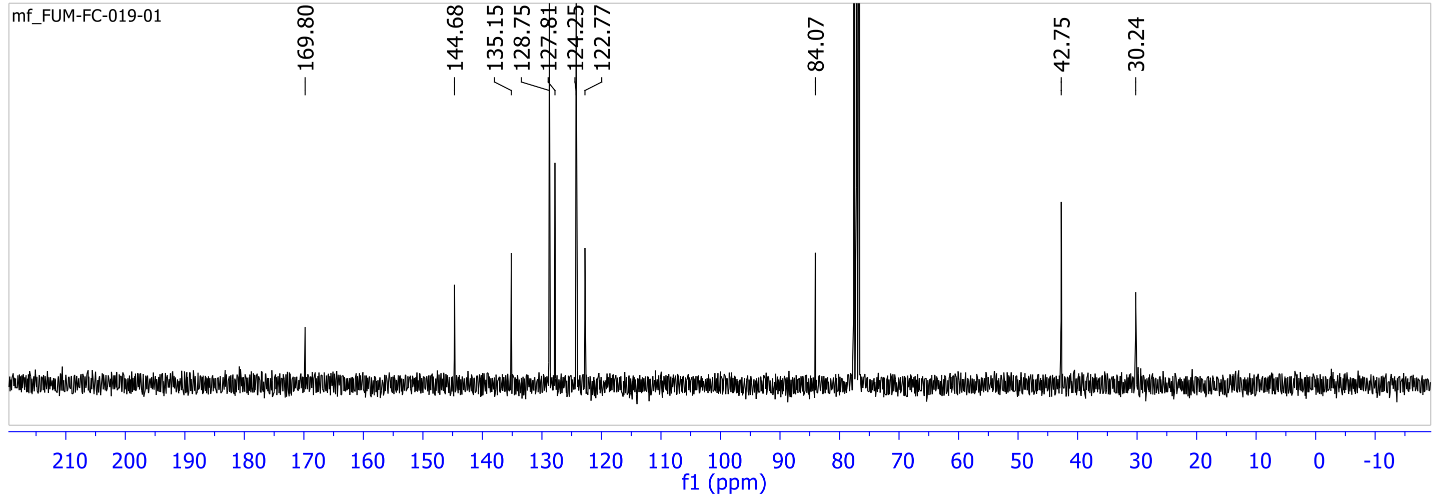

Benzhydryl 4-hydroxy-2-methylene-4-phenylpentanoate (**8e**) in $CDCl_3$ @ 300.13 MHz (^1H) and 75. 47 MHz (^{13}C).

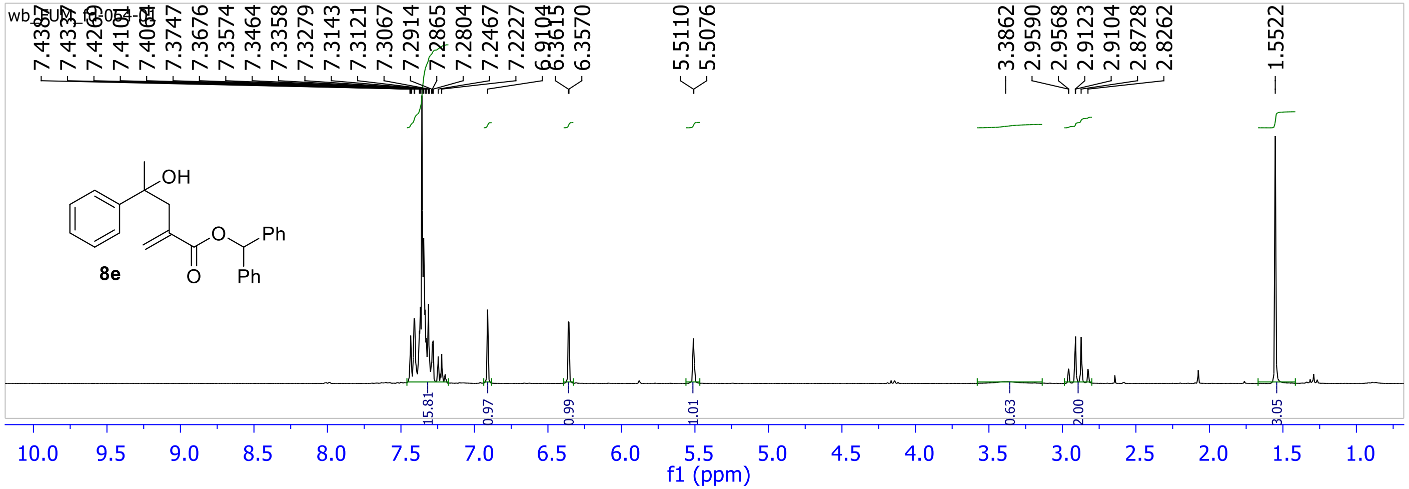

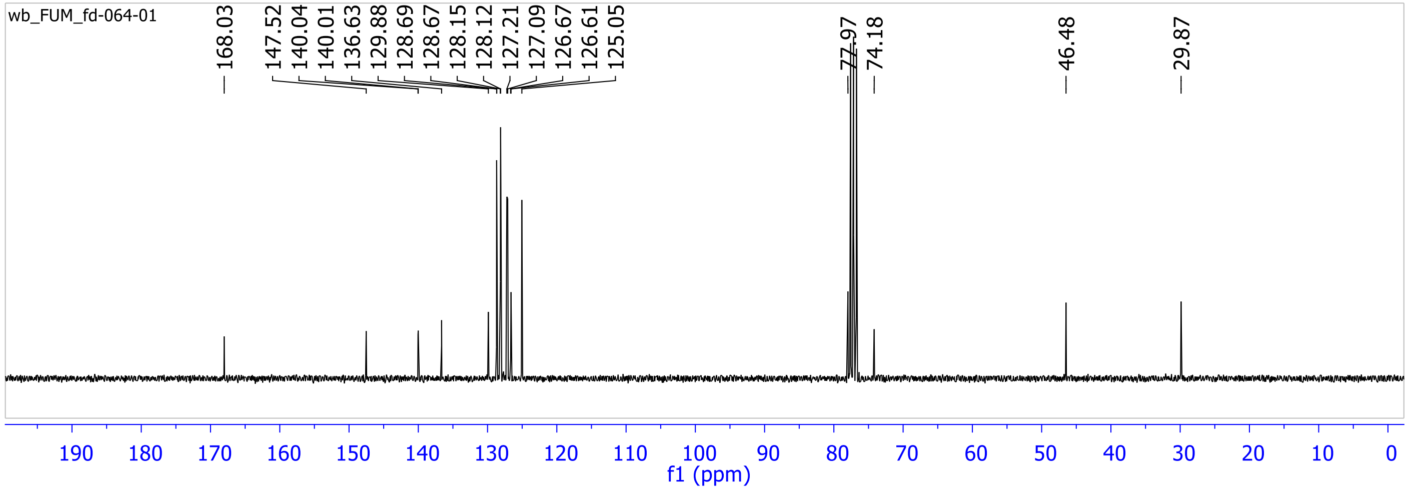

Benzhydryl 4-(4-bromophenyl)-4-hydroxy-2-methylenepentanoate (**8f**) in $CDCl_3$ @ 300.13 MHz (^1H) and 75. 47 MHz (^{13}C).

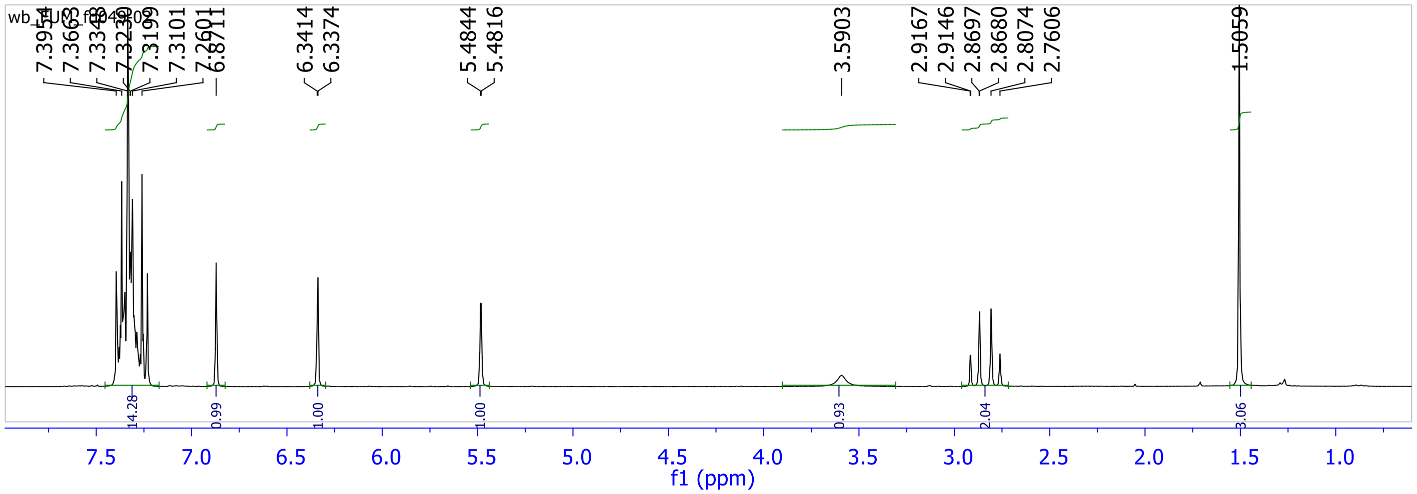

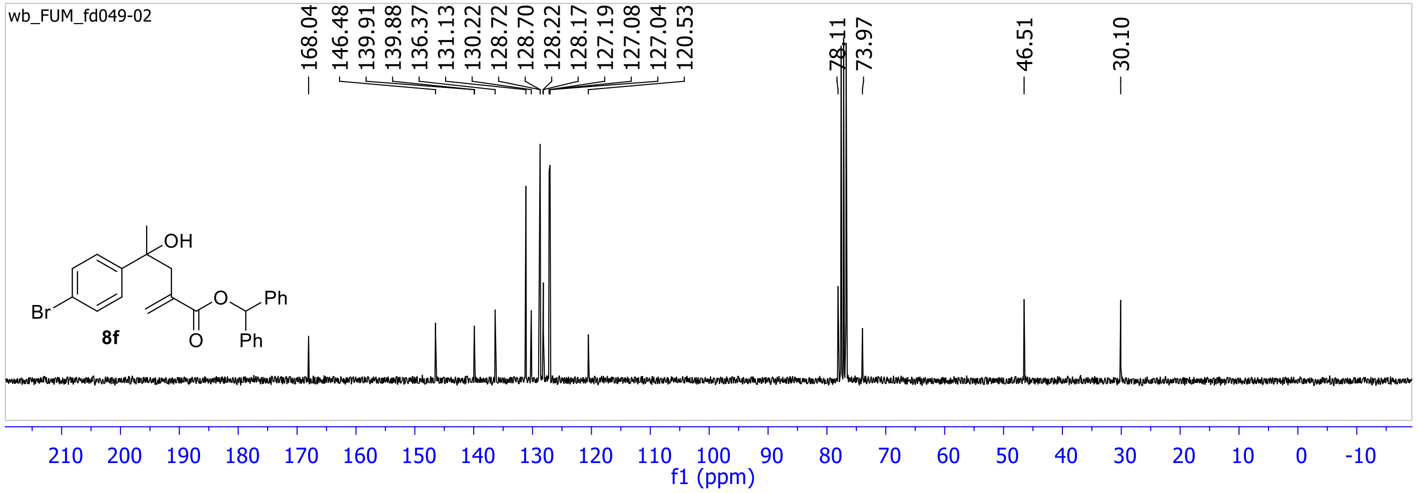

Benzhydryl 4-hydroxy-2-methylene-4-[4-(trifluoromethyl)phenyl]pentanoate (**8g**) in $CDCl_3$ @ 300.13 MHz (^{1}H), 282.39 MHz (^{19}F) and 75. 47 MHz (^{13}C; NMR spectra are shown in the same order with extensions of the ^{13}C-spectrum).

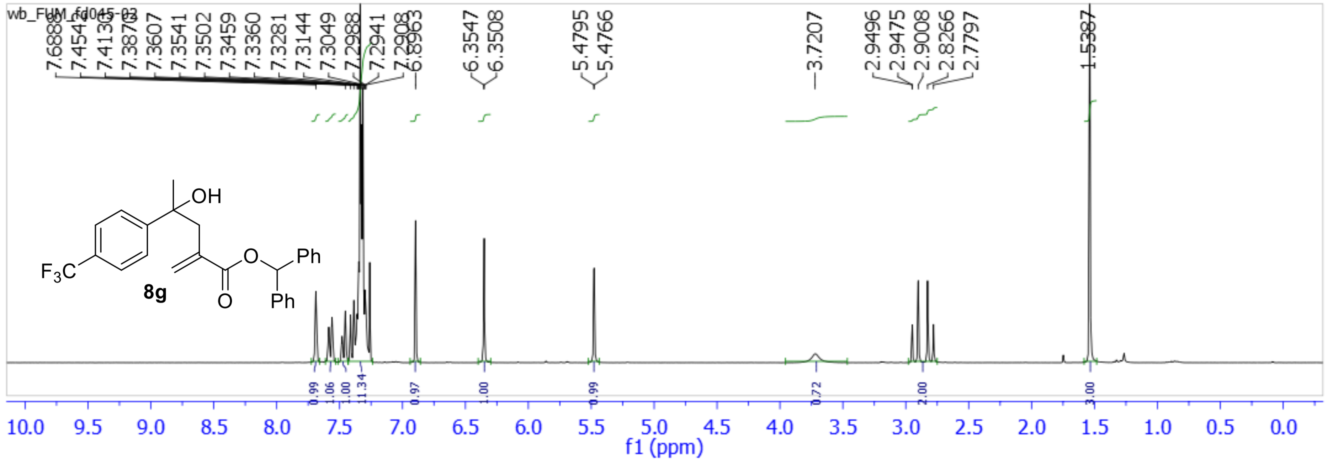

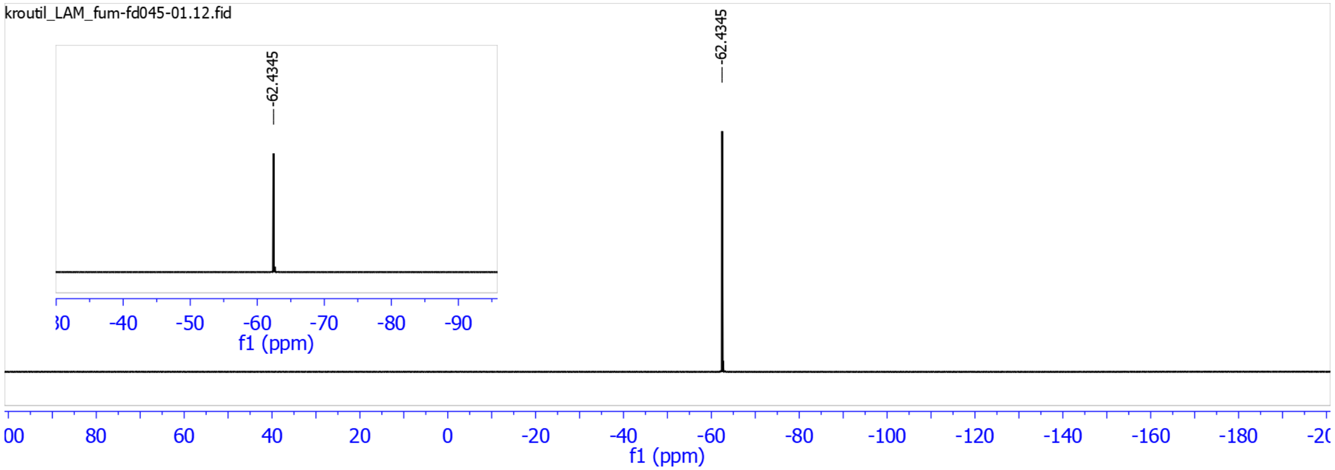

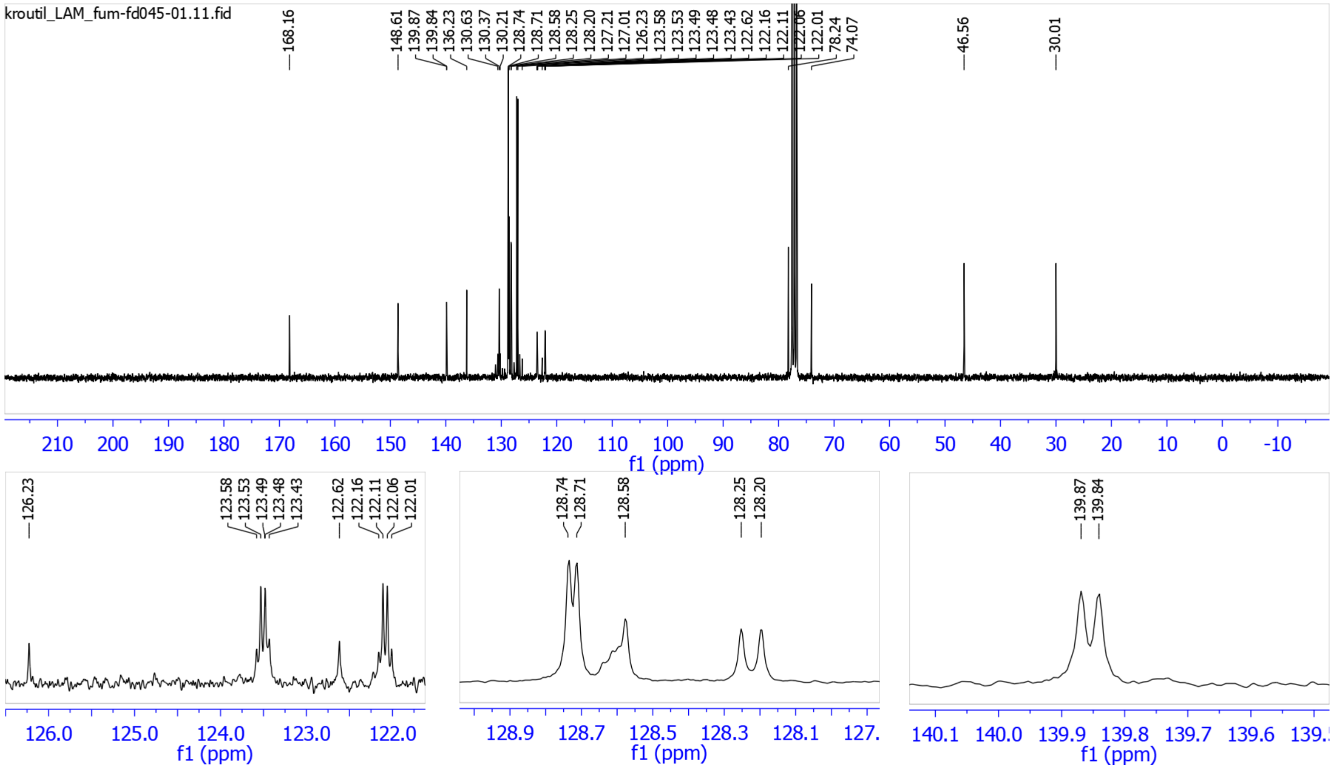
kroutil_LAM_fum-fd045-01.11.fid
168.16
148.61
139.87
139.84
136.23
130.63
130.37
130.21
128.74
128.71
128.58
128.25
128.20
127.21
127.01
126.23
123.58
123.53
123.49
123.48
123.43
122.62
122.16
122.11
122.06
122.01
78.24
74.07
46.56
30.01
f1 (ppm)

Benzhydryl 4-hydroxy-4-(4-methoxyphenyl)-2-methylenepentanoate (**8h**) in $CDCl_3$ @ 300.13 MHz (^1H) and 75. 47 MHz (^{13}C).

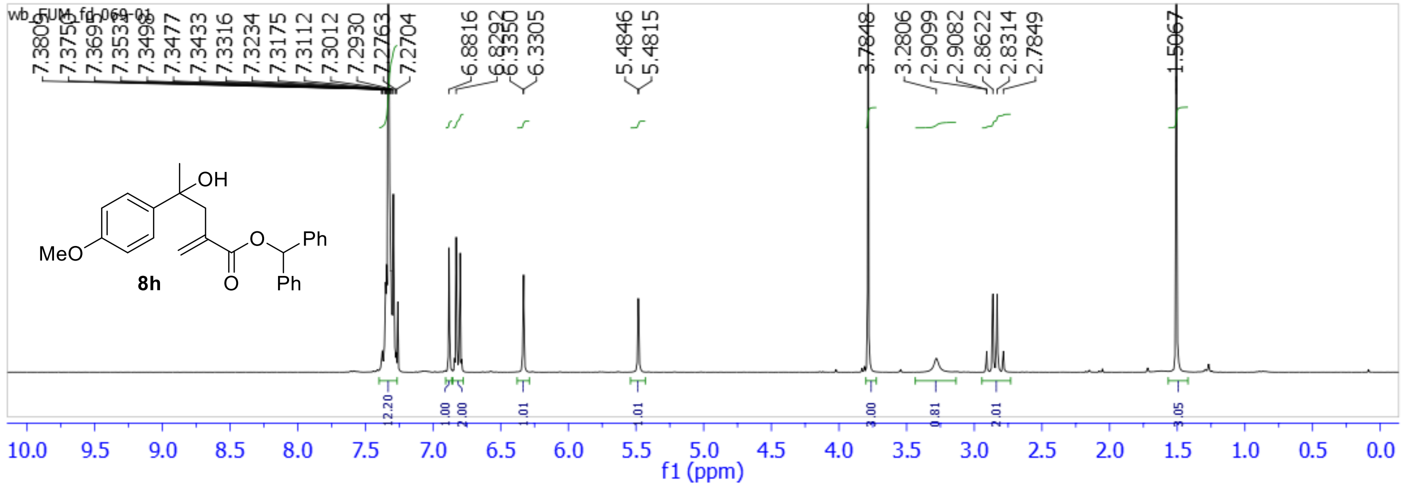

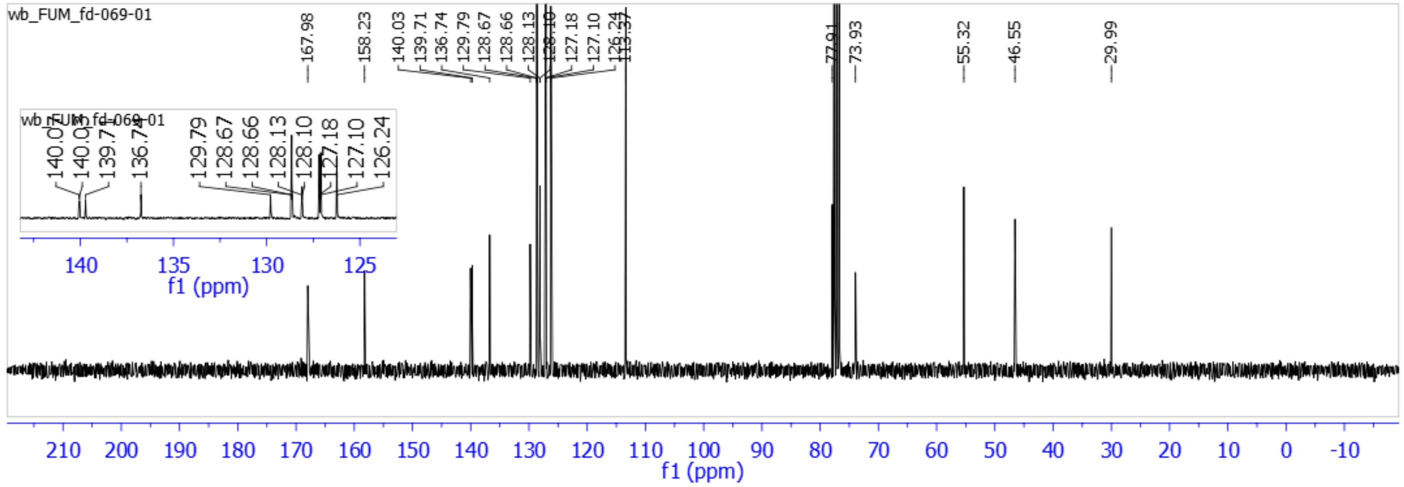

Benzhydryl 4-hydroxy-4-(3-methoxyphenyl)-2-methylenepentanoate (**8i**) in $CDCl_3$ @ 300.13 MHz (^{1}H) and 75. 47 MHz (^{13}C).

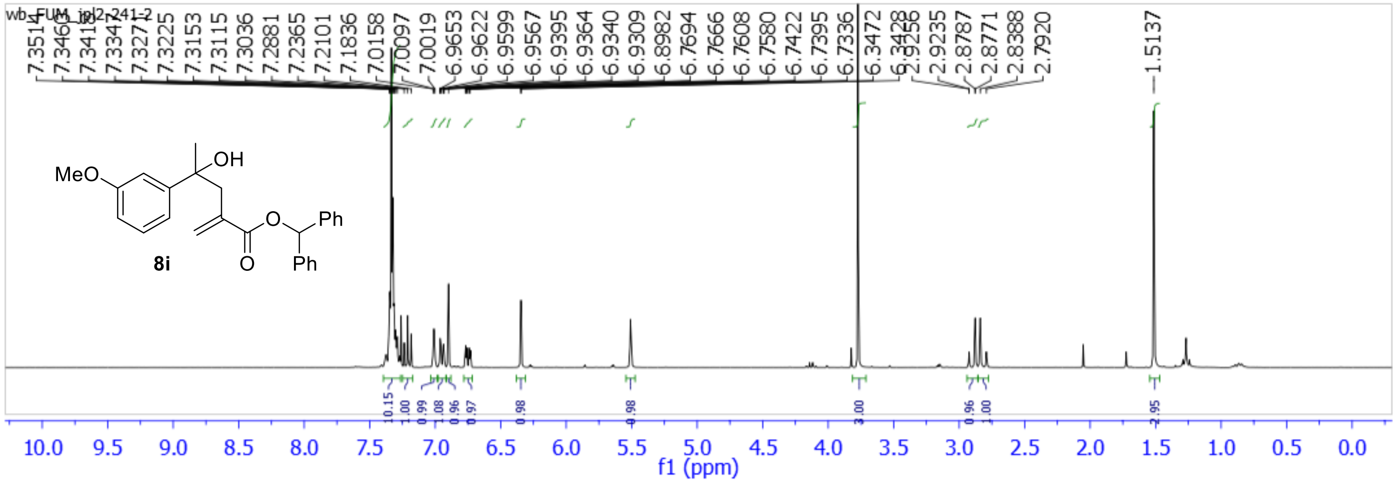

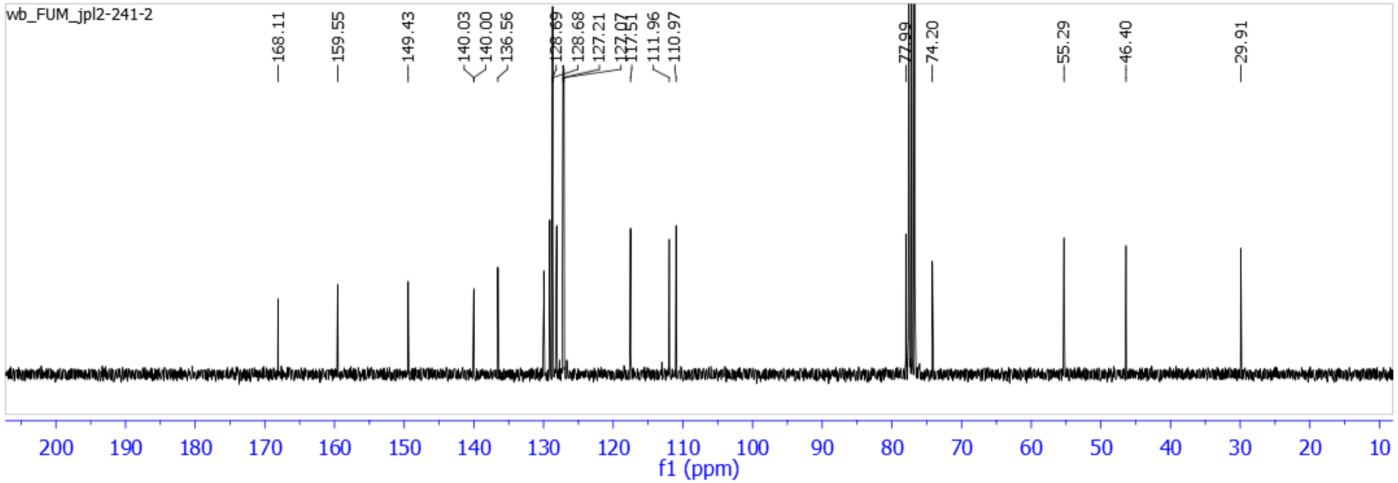

Benzhydryl 4-hydroxy-4-(2-methoxyphenyl)-2-methylenepentanoate (8j) in $CDCl_3$ @ 300.13 MHz (^{1}H) and 75. 47 MHz (^{13}C).

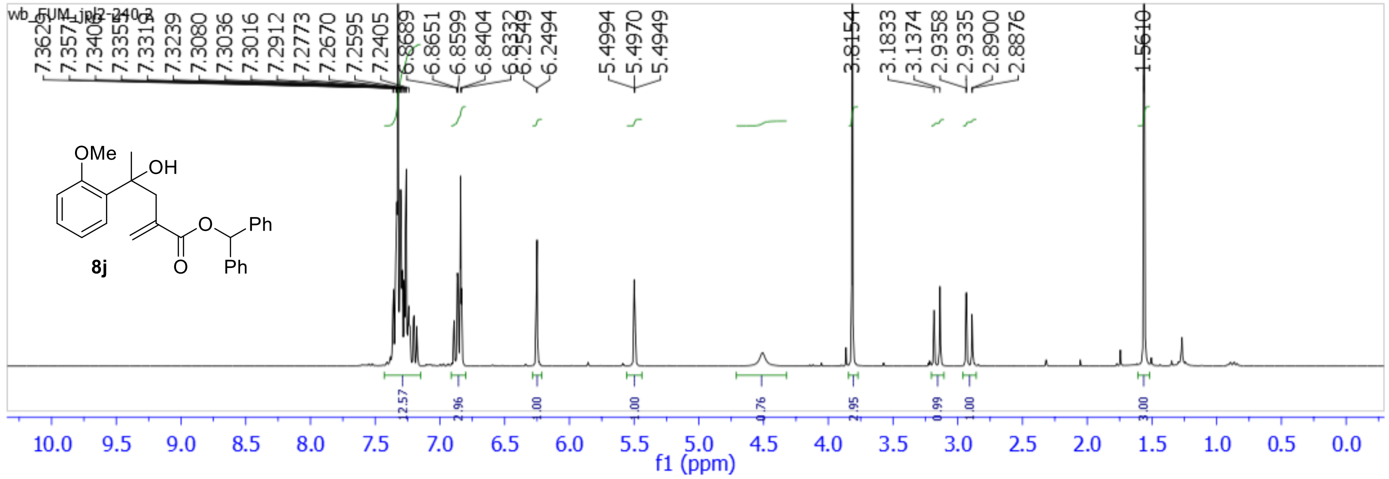

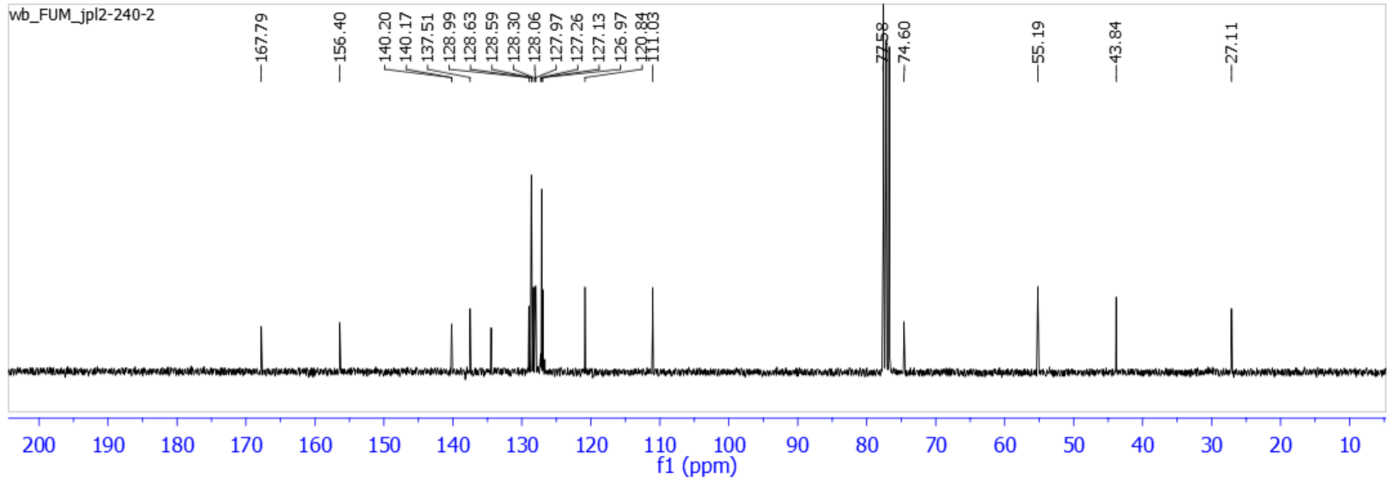

Benzhydryl 4-hydroxy-2-methylene-4-phenylhexanoate (**8k**) in $CDCl_3$ @ 300.13 MHz (^{1}H) and 75. 47 MHz (^{13}C).

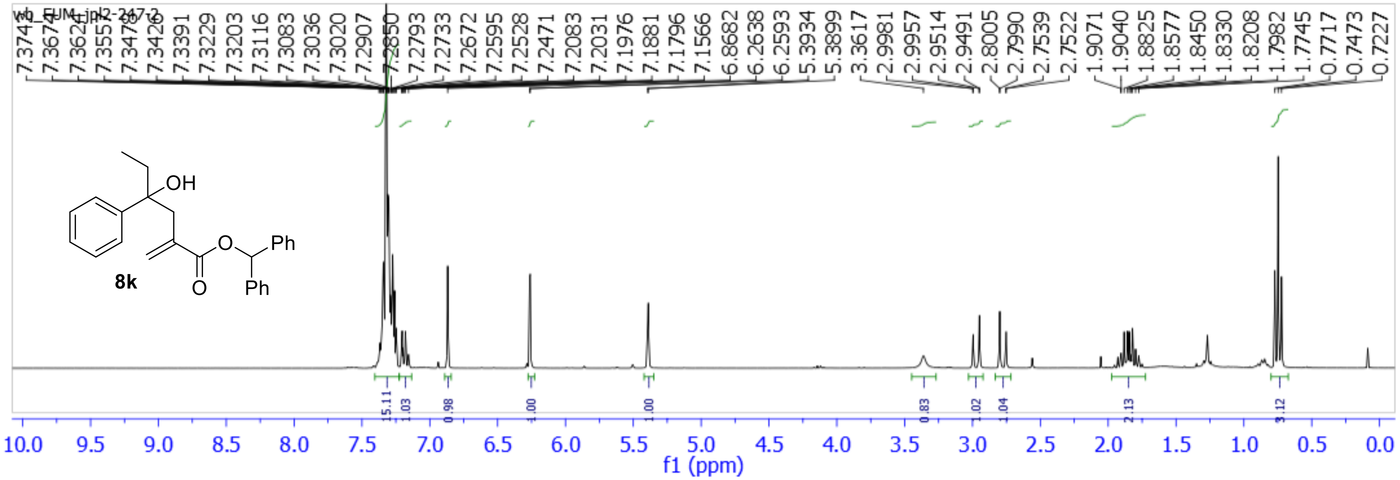

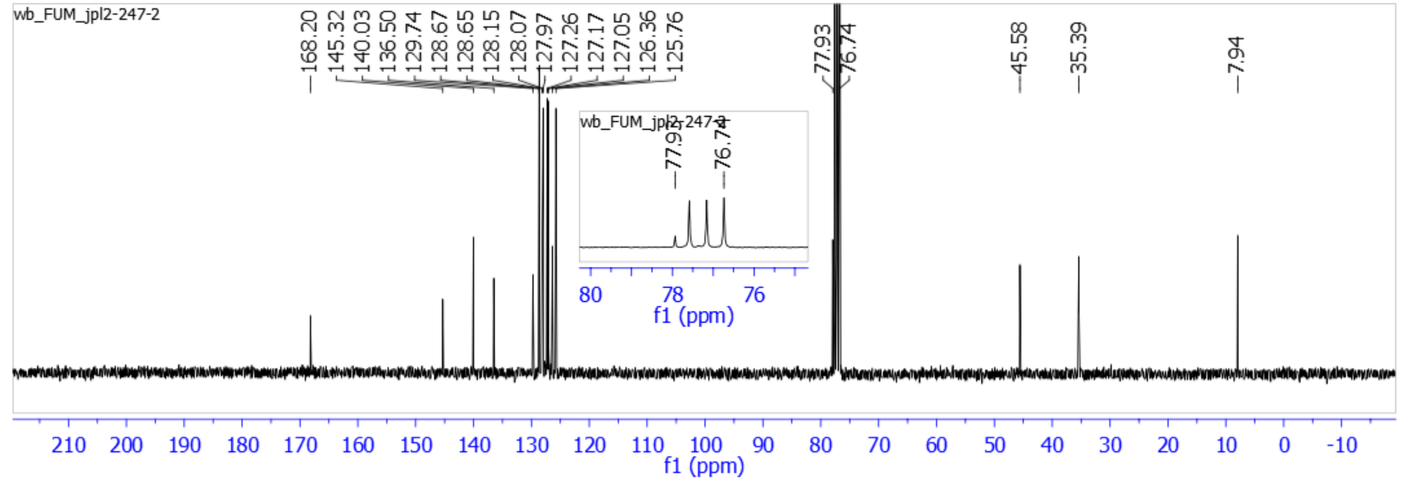

Benzhydryl 4-hydroxy-2-methylene-4-phenylheptanoate (**8l**) in $CDCl_3$ @ 300.13 MHz (^1H) and 75. 47 MHz (^{13}C).

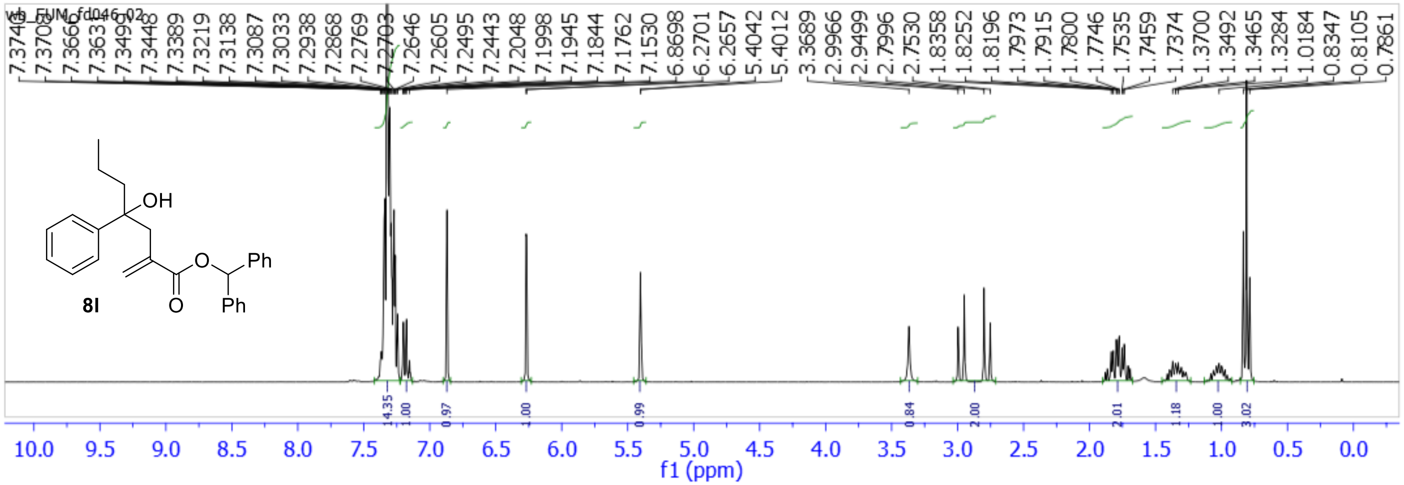

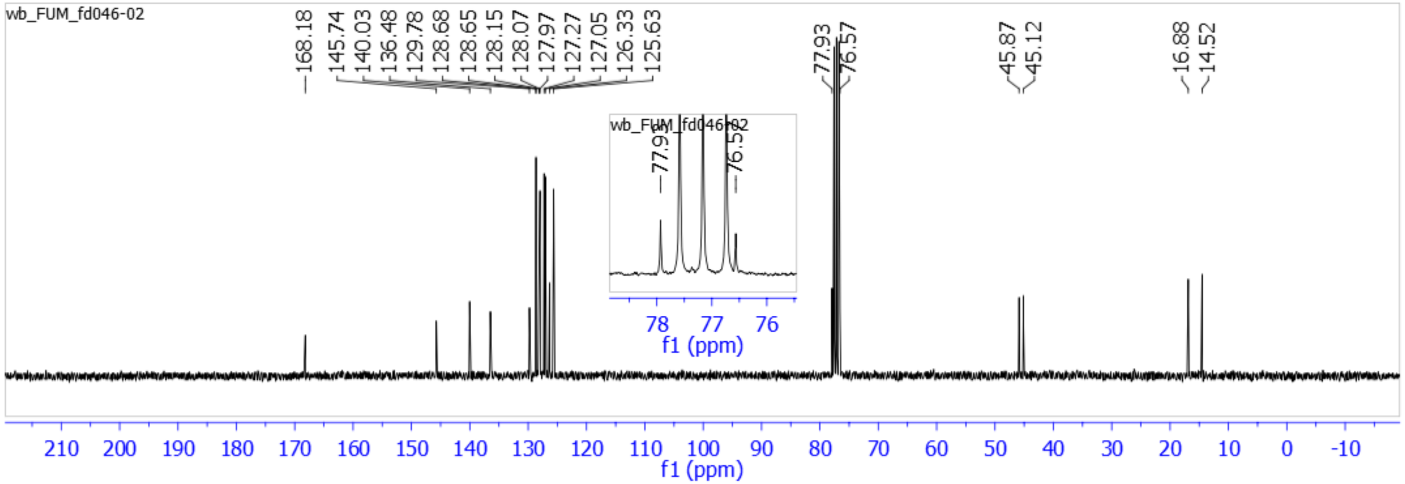

Benzhydryl 2-[(1-hydroxy-1,2,3,4-tetrahydronaphthalen-1-yl)methyl]acrylate (**8m**) in $CDCl_3$ @ 300.13 MHz (^1H) and 75. 47 MHz (^{13}C).

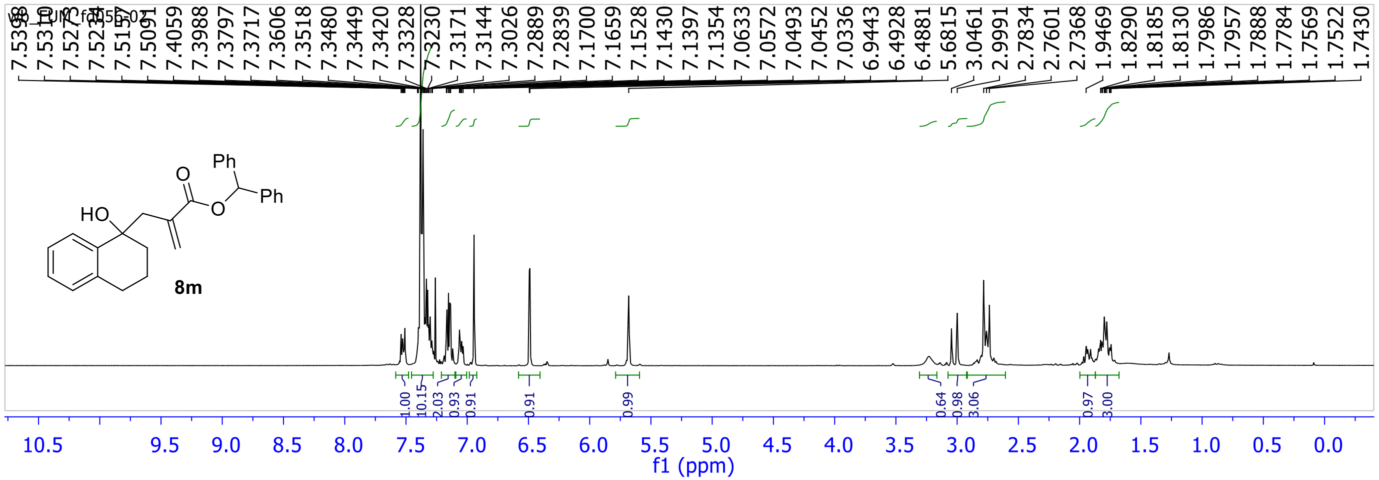

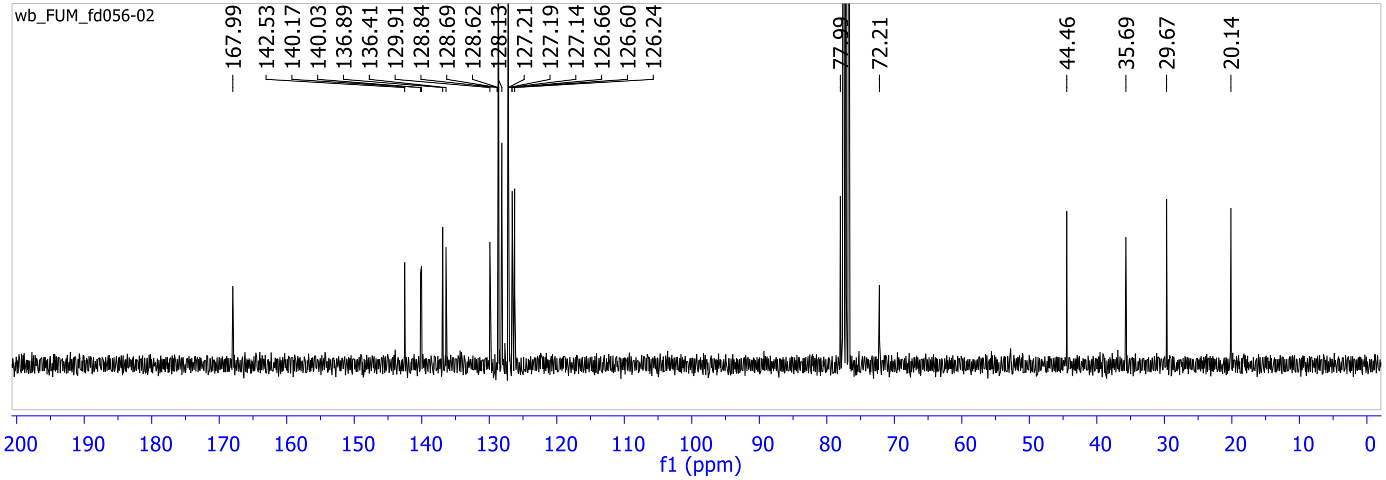

Benzhydryl 2-[(4-hydroxychroman-4-yl)methyl]acrylate (**8n**) in $CDCl_3$ @ 300.13 MHz (^1H) and 75. 47 MHz (^{13}C).

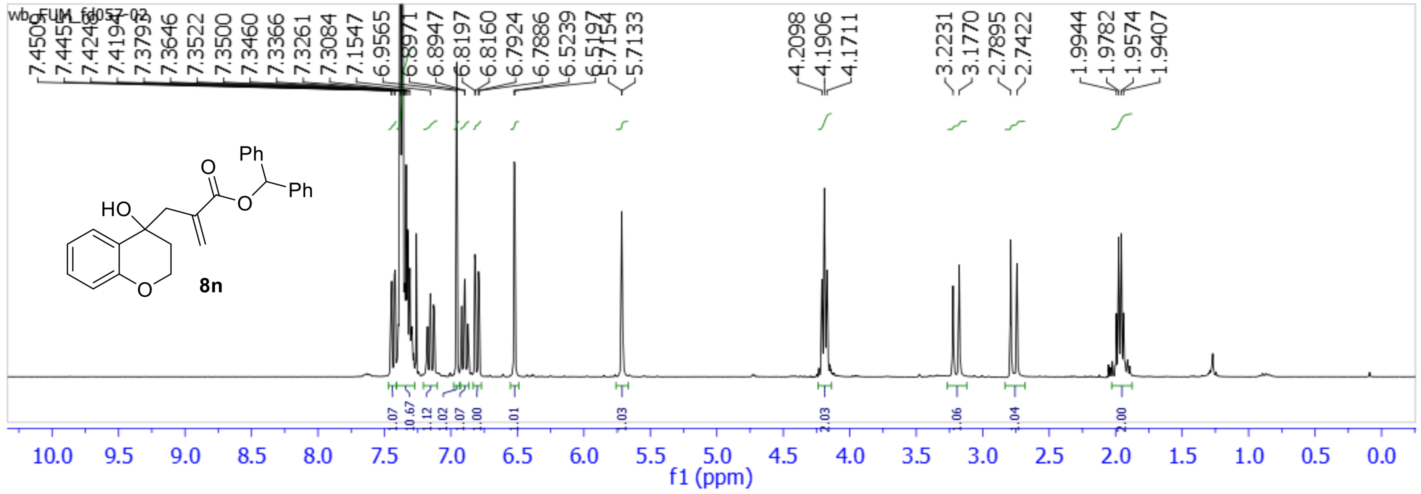

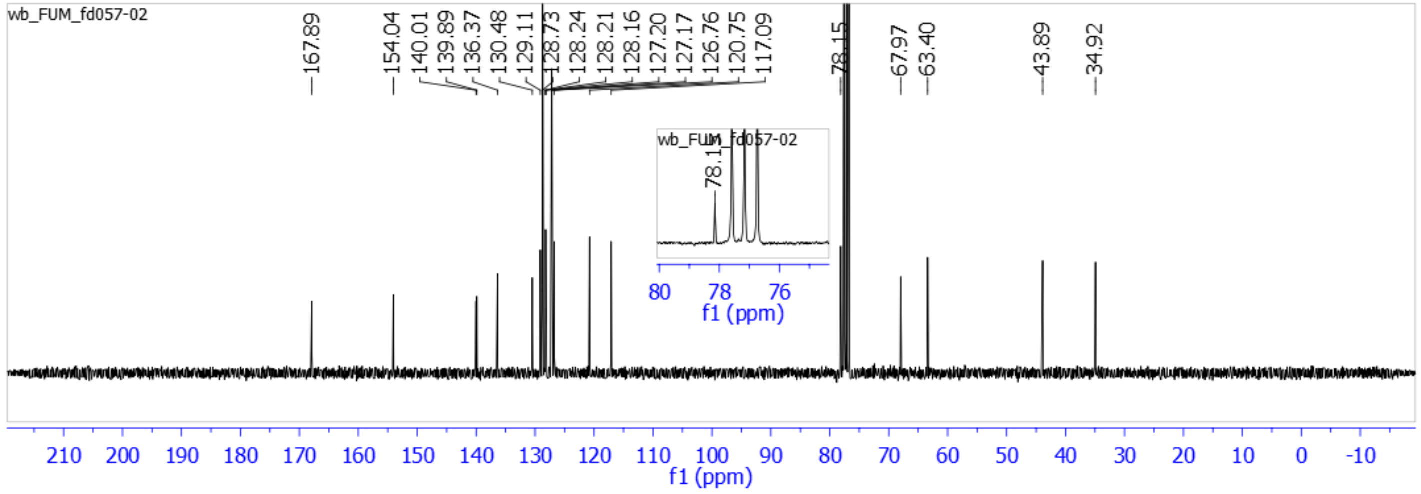

Benzhydryl 2-[(6-chloro-4-hydroxychroman-4-yl)methyl]acrylate **(8o)** in $CDCl_3$ @ 300.13 MHz (^{1}H) and 75. 47 MHz (^{13}C).

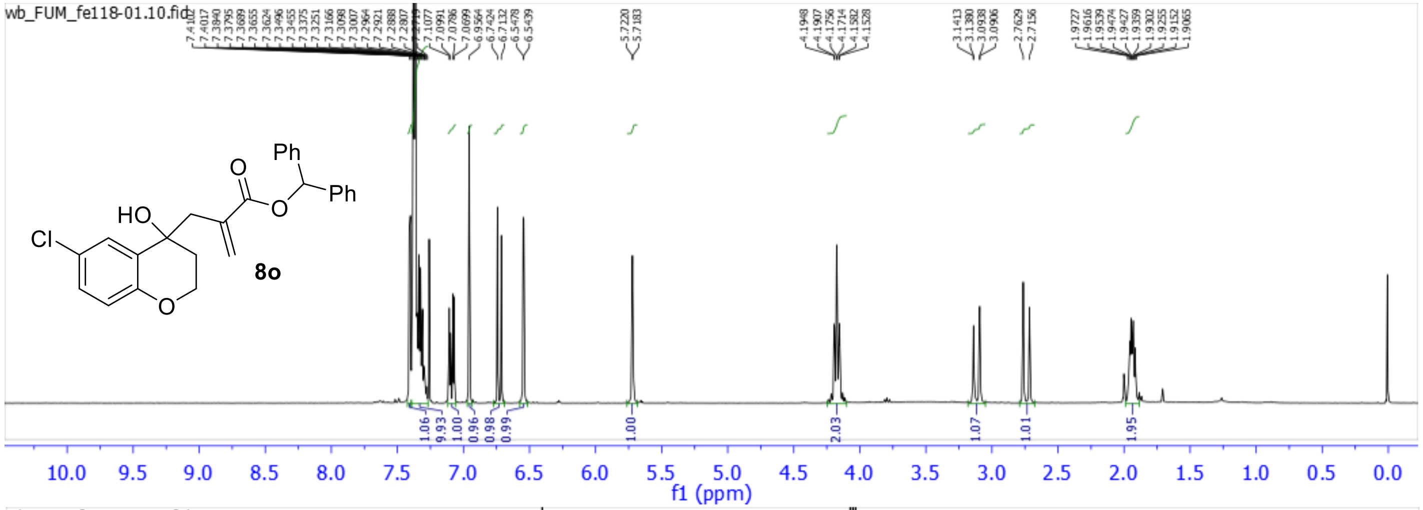

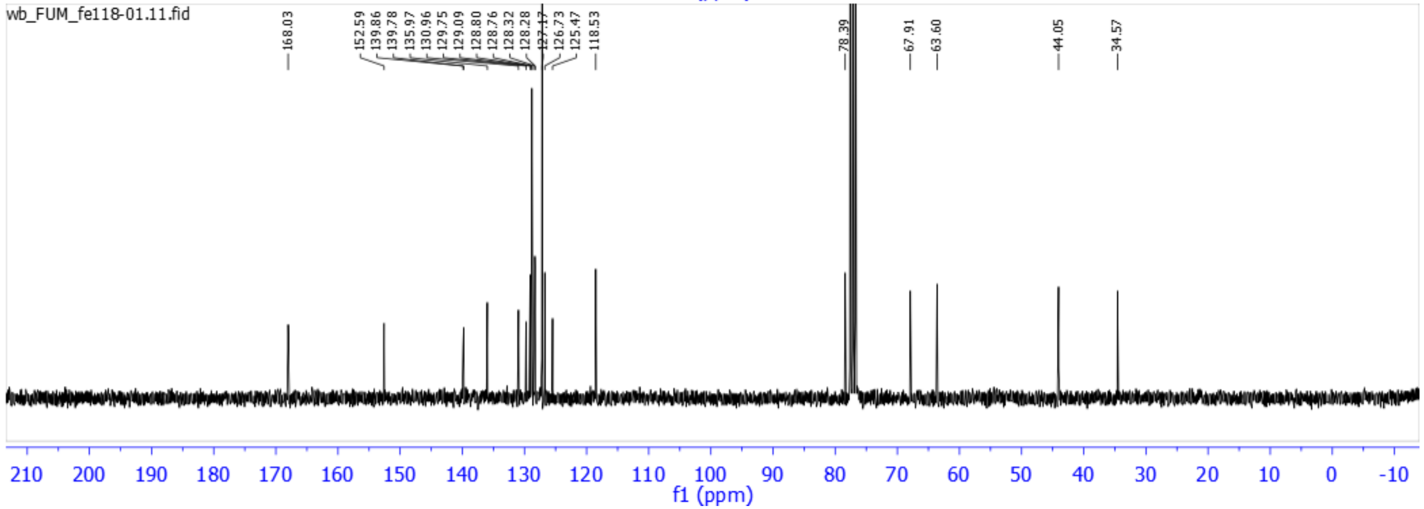

Benzhydryl (*E*)-4-hydroxy-4-methyl-2-methylene-6-phenylhex-5-enoate (**8p**) in $CDCl_3$ @ 300.13 MHz (^1H) and 75. 47 MHz (^{13}C).

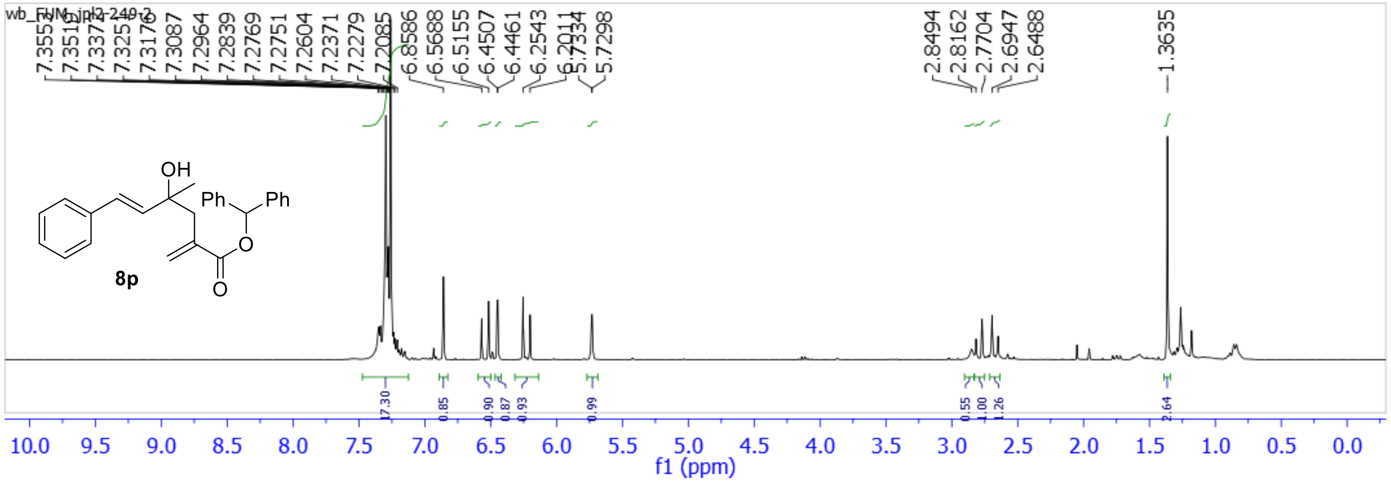

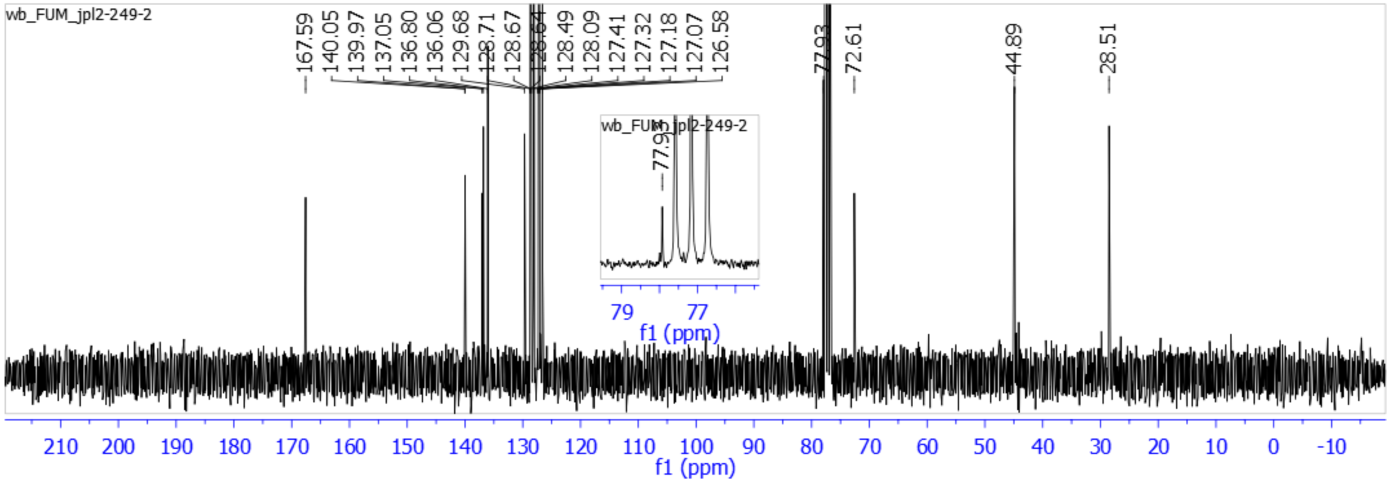

Benzhydryl 4-(4-bromophenyl)-4-hydroxy-3-methyl-2-methylenepentanoate (**8q**) in $CDCl_3$ @ 300.13 MHz (^1H) and 75. 47 MHz (^{13}C).

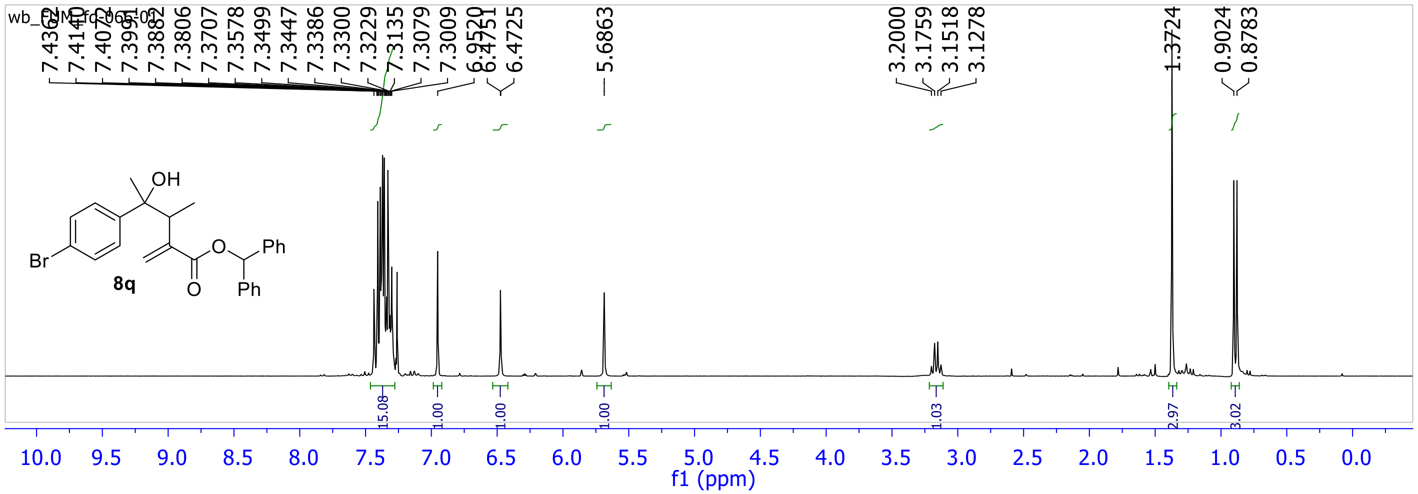

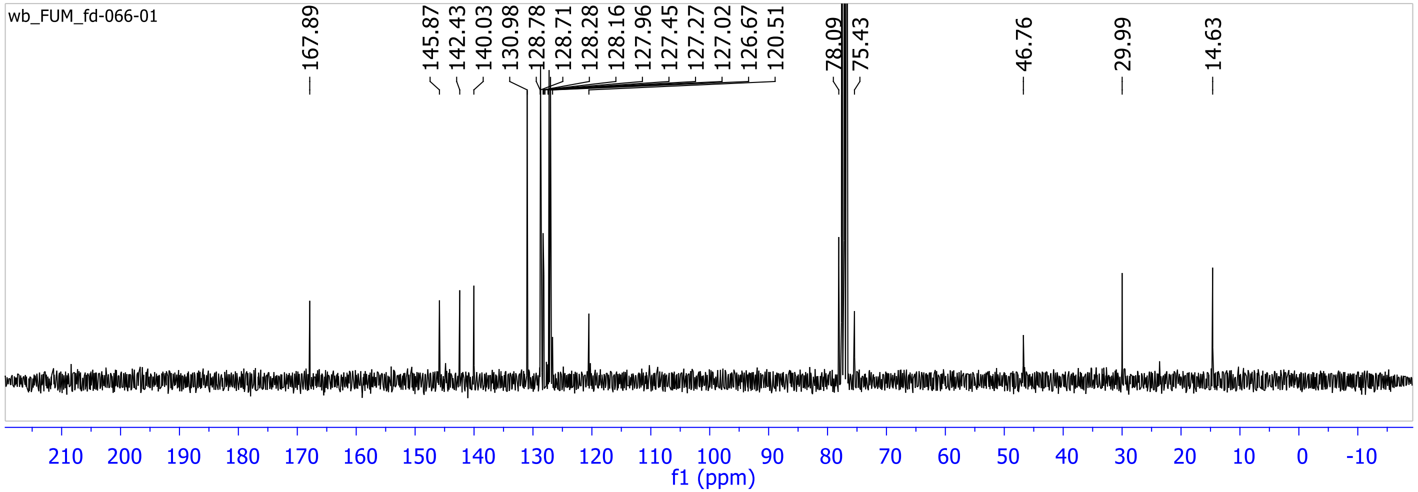

(*R*)-6-chloro-4'-methylene-3',4'-dihydro-5'H-spiro[chromane-4,2'-furan]-5'-one **(10o)** in $CDCl_3$ @ 300.13 MHz (^1H) and 75. 47 MHz (^{13}C).

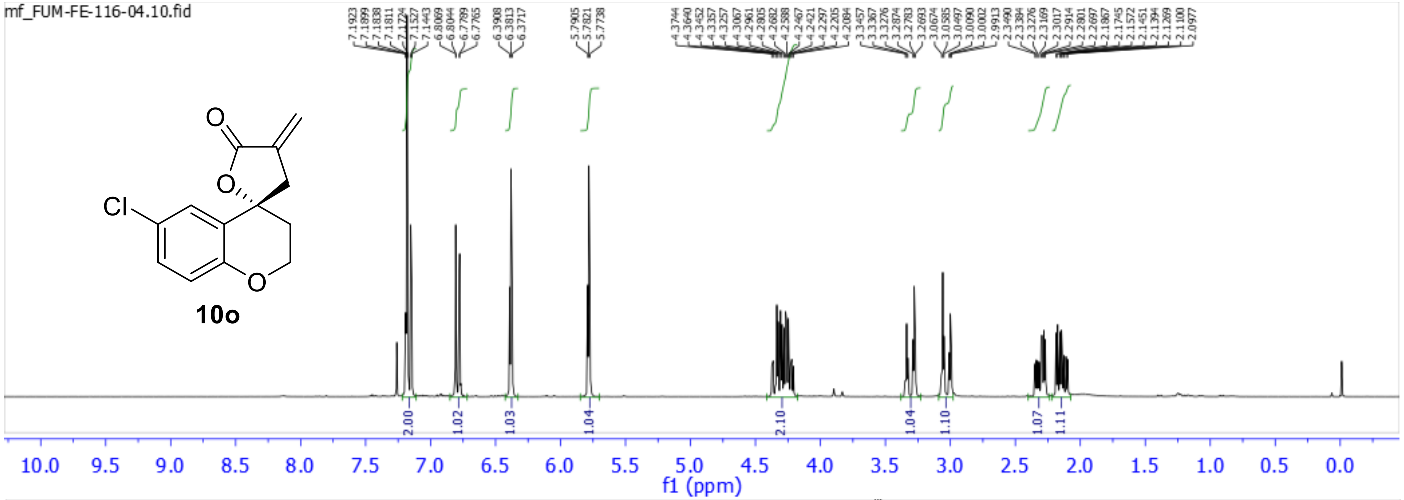

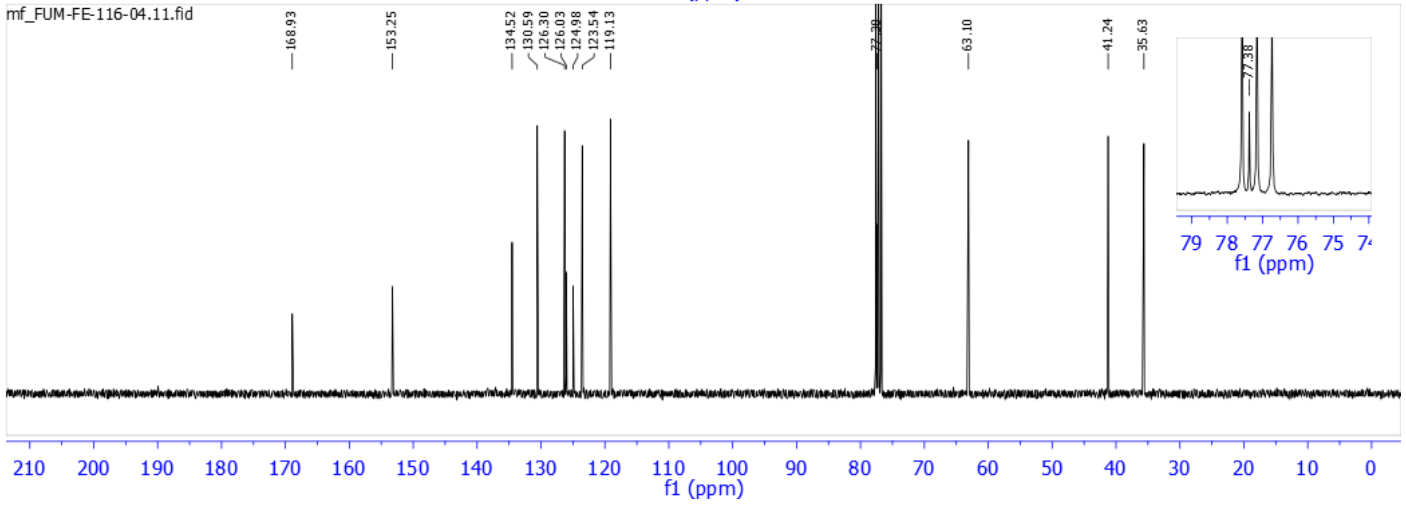

5-(4-bromophenyl)-4,5-dimethyl-3-methylenedihydrofuran-2(3H)-one (**10q**) in $CDCl_3$ @ 300.13 MHz (^{1}H) and 75. 47 MHz (^{13}C).

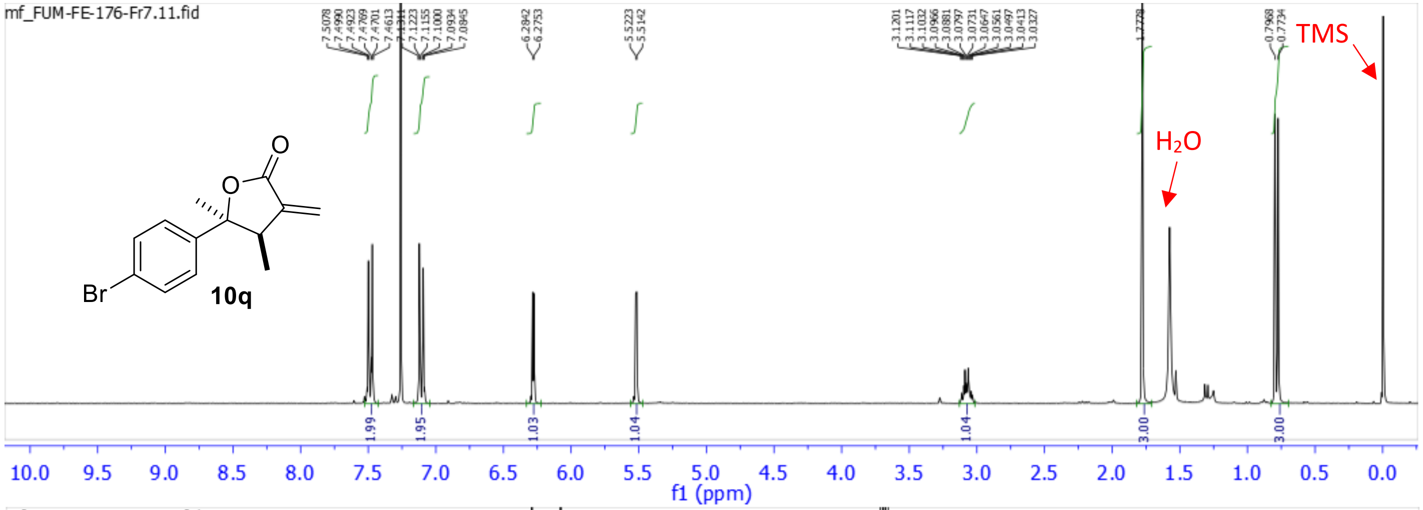

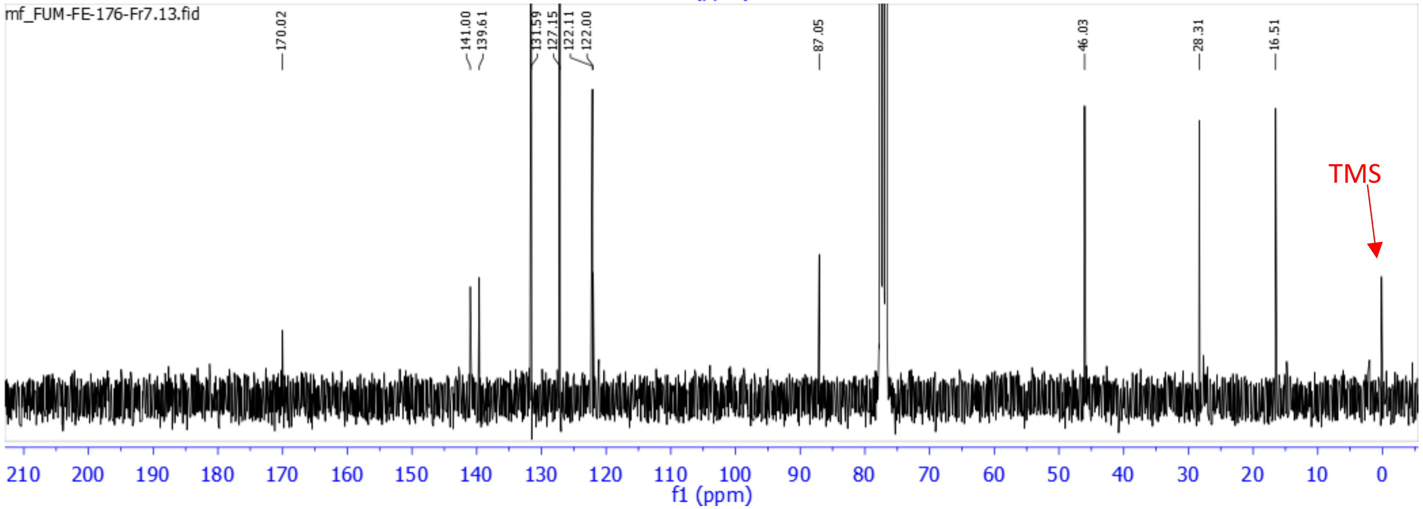

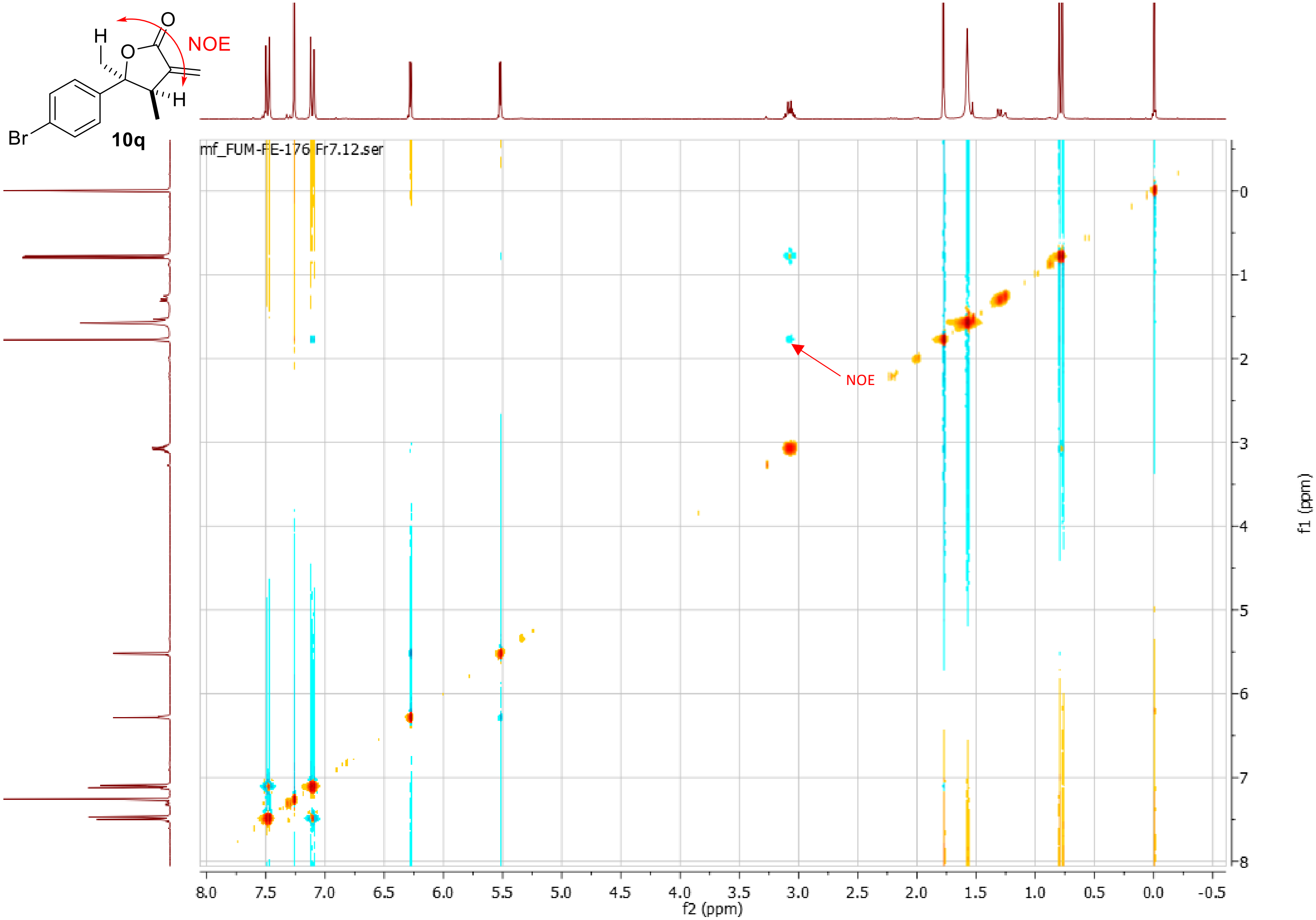
NOE
H
O
O
H
Br
10q
mf_FUM-FE-176 Fr7.12.ser
NOE
f2 (ppm)
f1 (ppm)

4-[(4-bromophenyl)(hydroxy)methyl]-3-methylenedihydrofuran-2(3*H*)-one (**19**) in $CDCl_3$ @ 300.13 MHz (^{1}H) and 75. 47 MHz (^{13}C).

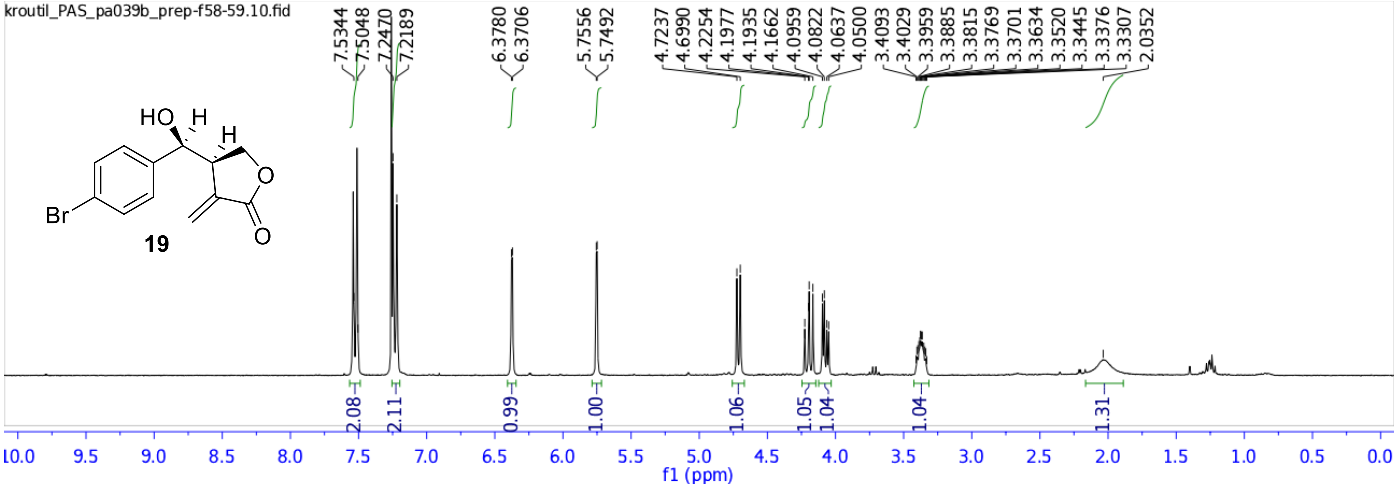

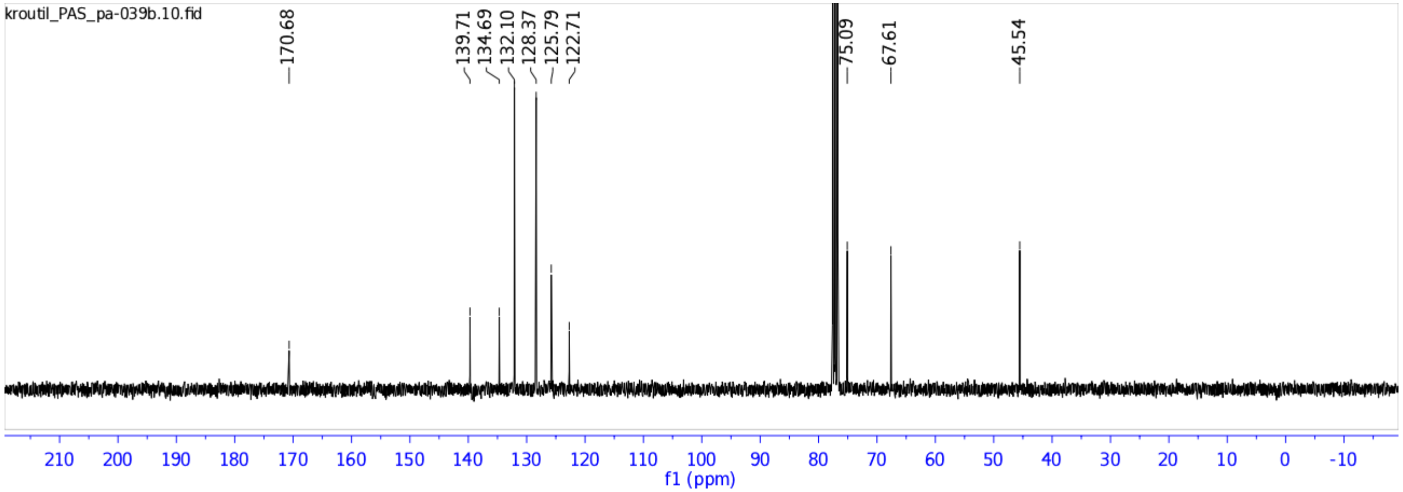

4-[1-(4-bromophenyl)-1-hydroxyethyl]-3-methylenedihydrofuran-2(3*H*)-one (**20**) in $CDCl_3$ @ 300.13 MHz (^1H) and 75. 47 MHz (^{13}C).

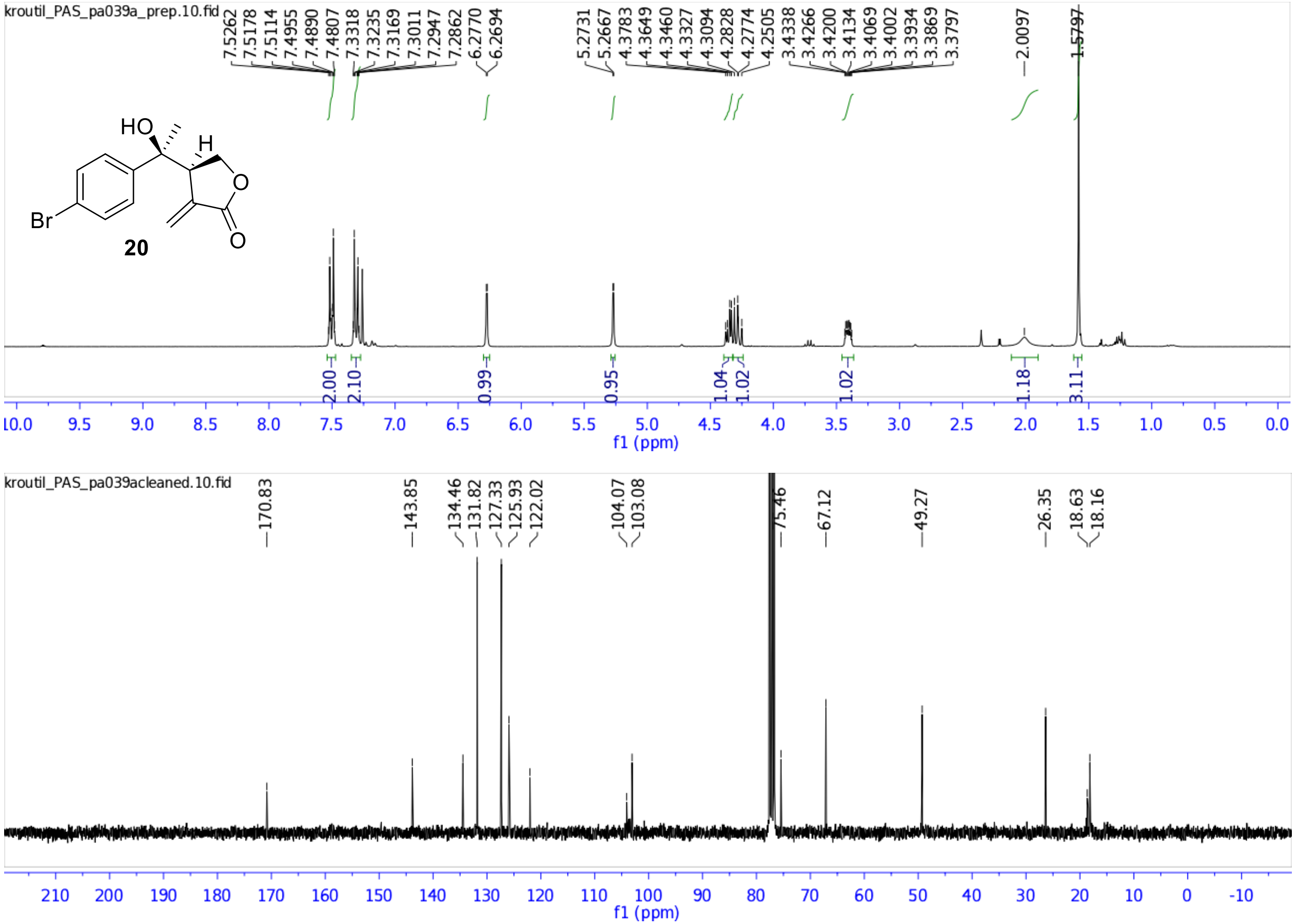

N,*N*-dibenzyl-4-hydroxy-2-methylene-4-phenylpentanamide (**8r**):

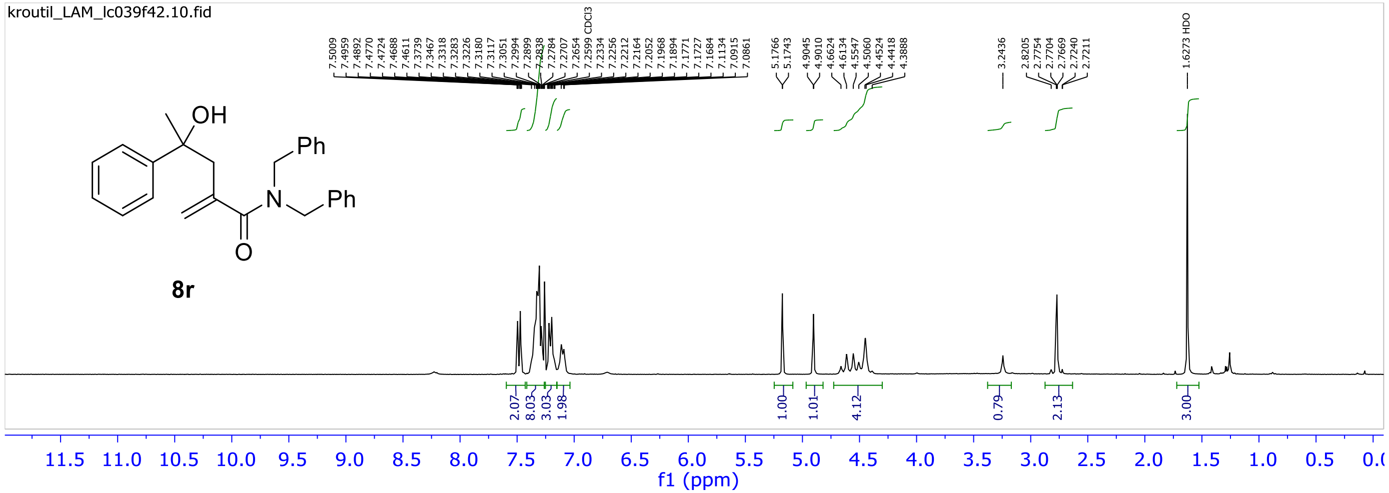

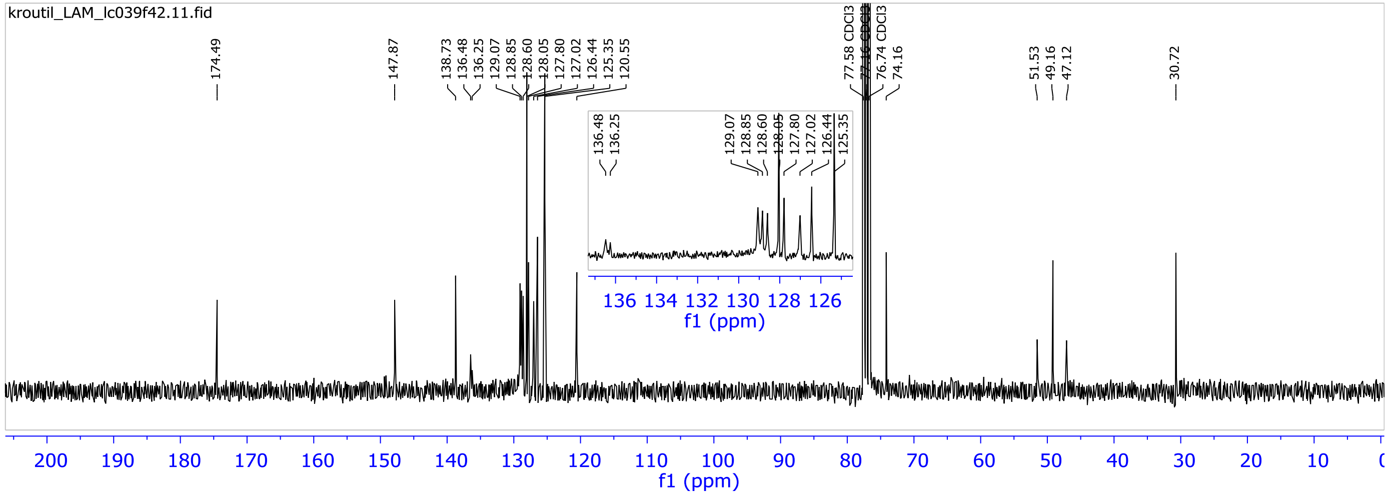

HPLC chromatograms

Benzhydryl 4-hydroxy-2-methylene-4-phenylpentanoate (8e).

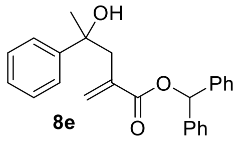

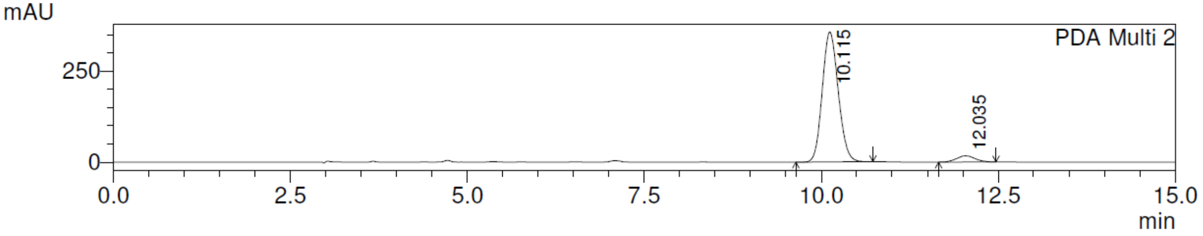

PeakTable

PDA Ch2 230nm 4nm

Peak#	Ret. Time	Area	Height	Area %	Height %
1	10.115	5611474	357534	94.752	95.497
2	12.035	310810	16859	5.248	4.503
Total		5922284	374394	100.000	100.000

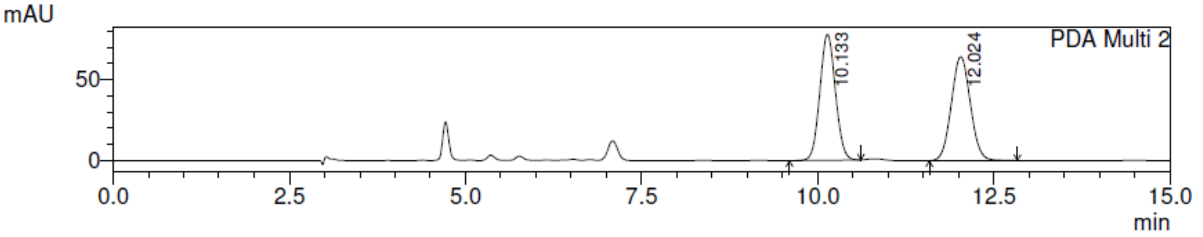

PeakTable

PDA Ch2 230nm 4nm

Peak#	Ret. Time	Area	Height	Area %	Height %
1	10.133	1193124	77491	49.851	54.769
2	12.024	1200273	63995	50.149	45.231
Total		2393397	141485	100.000	100.000

Benzhydryl 4-(4-bromophenyl)-4-hydroxy-2-methylenepentanoate (8f).

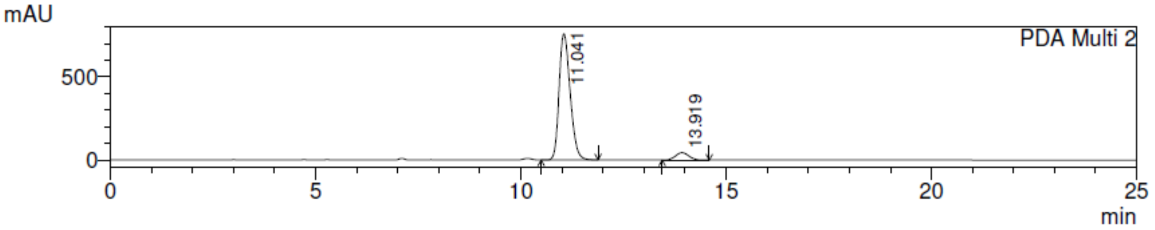

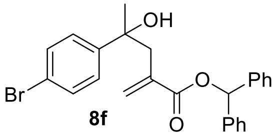

8f

PeakTable

PDA Ch2 230nm 4nm

Peak#	Ret. Time	Area	Height	Area %	Height %
1	11.041	14201746	759697	93.514	94.525
2	13.919	985047	44002	6.486	5.475
Total		15186793	803698	100.000	100.000

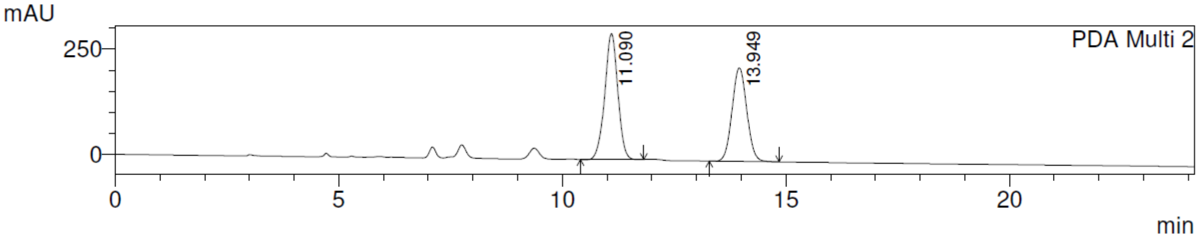

PeakTable

PDA Ch2 230nm 4nm

Peak#	Ret. Time	Area	Height	Area %	Height %
1	11.090	6086907	299540	54.342	57.356
2	13.949	5114302	222703	45.658	42.644
Total		11201209	522243	100.000	100.000

Benzhydryl 4-hydroxy-2-methylene-4-[4-(trifluoromethyl)phenyl]pentanoate (**8g**).

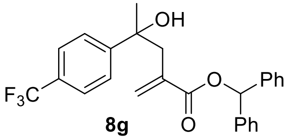

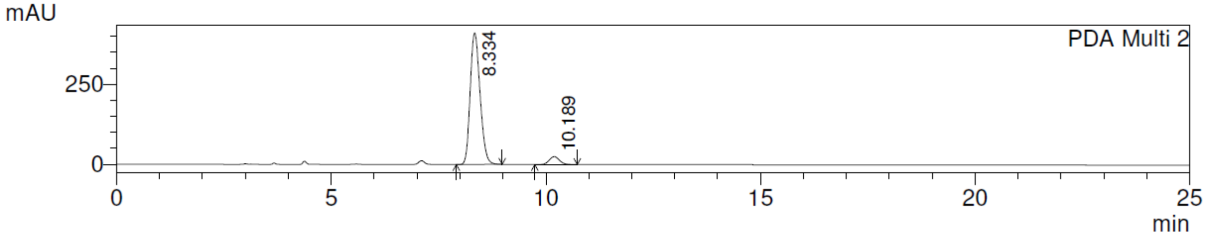

PeakTable

PDA Ch2 230nm 4nm

Peak#	Ret. Time	Area	Height	Area %	Height %
1	8.334	6560499	410990	94.153	94.310
2	10.189	407421	24797	5.847	5.690
Total		6967920	435787	100.000	100.000

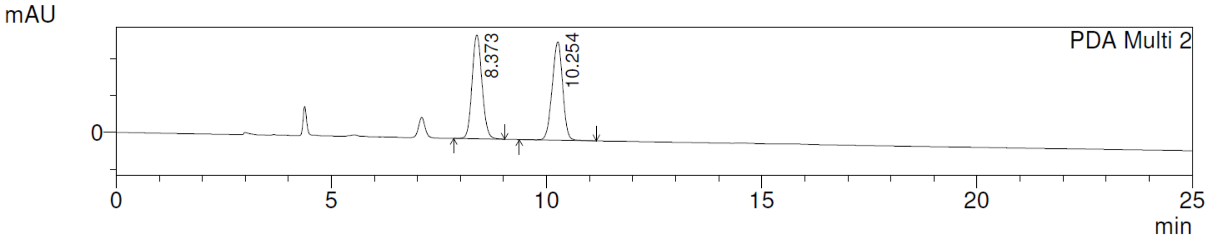

PeakTable

PDA Ch2 230nm 4nm

Peak#	Ret. Time	Area	Height	Area %	Height %
1	8.373	2258385	140588	50.074	51.317
2	10.254	2251707	133372	49.926	48.683
Total		4510092	273960	100.000	100.000

Benzhydryl 4-hydroxy-4-(4-methoxyphenyl)-2-methylenepentanoate (**8h**).

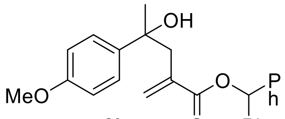

8h

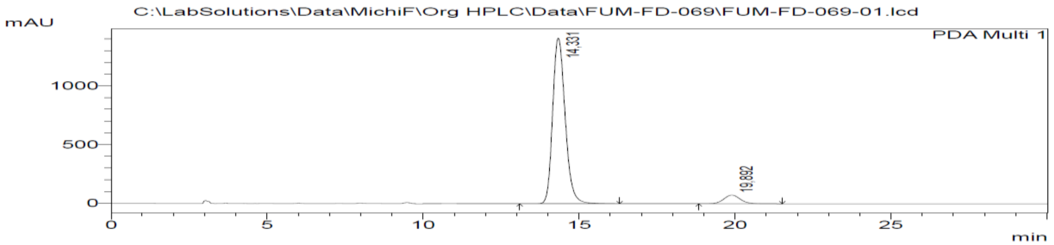

PeakTable

PDA Ch1 215nm

Peak#	Ret. Time	Area	Height	Area %	Height %
1	14,331	39311883	1410163	93,531	94,990
2	19,892	2719197	74371	6,469	5,010
Total		42031081	1484534	100,000	100,000

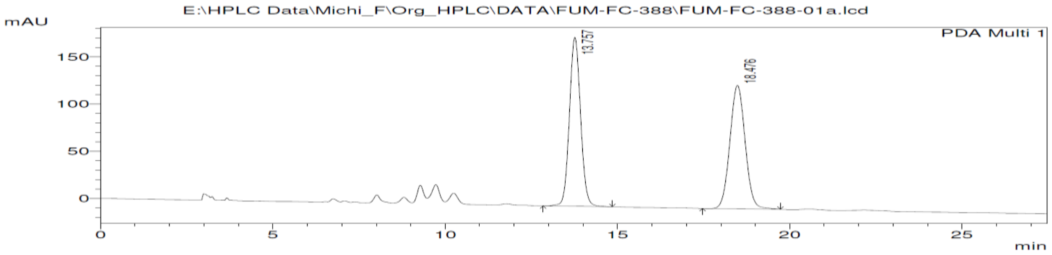

PeakTable

PDA Ch1 215nm 4nm

Peak#	Ret. Time	Area	Height	Area %	Height %
1	13.757	4032354	179012	50.039	57.775
2	18.476	4026087	130829	49.961	42.225
Total		8058441	309841	100.000	100.000

Benzhydryl 4-hydroxy-4-(3-methoxyphenyl)-2-methylenepentanoate (8i).

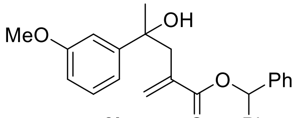

8i

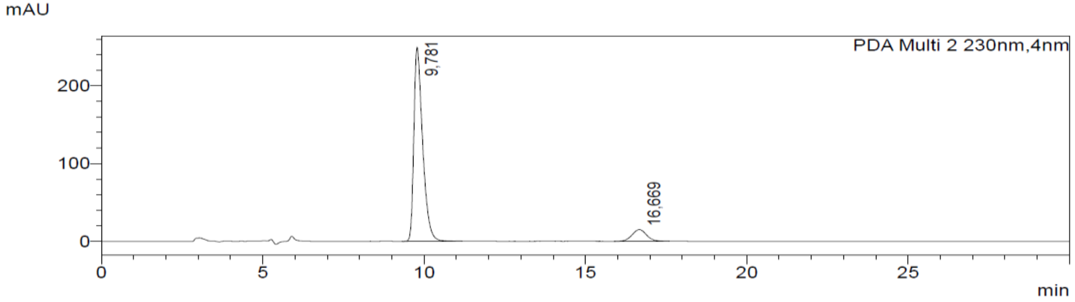

PDA Ch2 230nm

Peak#	Ret. Time	Name	Area	Height	Area%
1	9,781		4703597	249220	90,856
2	16,669		473409	15059	9,144
Total			5177006	264280	100,000

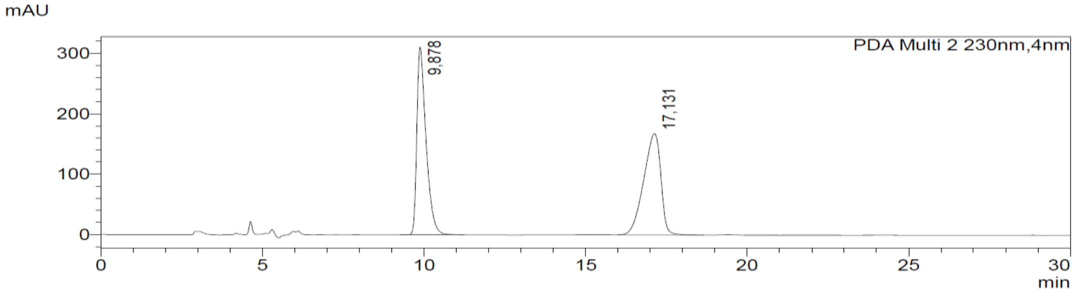

PDA Ch2 230nm

Peak#	Ret. Time	Name	Area	Height	Area%
1	9,878		6145262	310808	49,820
2	17,131		6189755	167775	50,180
Total			12335018	478582	100,000

Benzhydryl 4-hydroxy-4-(2-methoxyphenyl)-2-methylenepentanoate (8j).

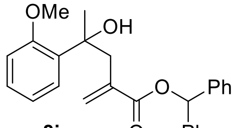

8j

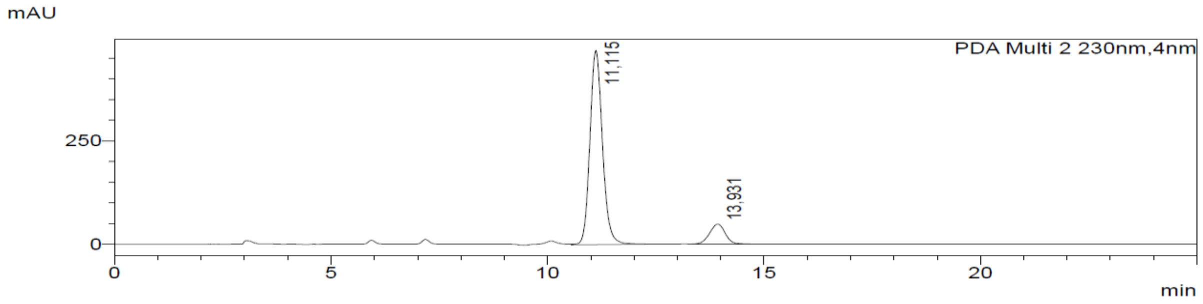

Peak#	Ret. Time	Name	Area	Height	Area%
1	11,115		9471125	468756	88,570
2	13,931		1222196	48329	11,430
Total			10693322	517086	100,000

PDA Ch2 230nm

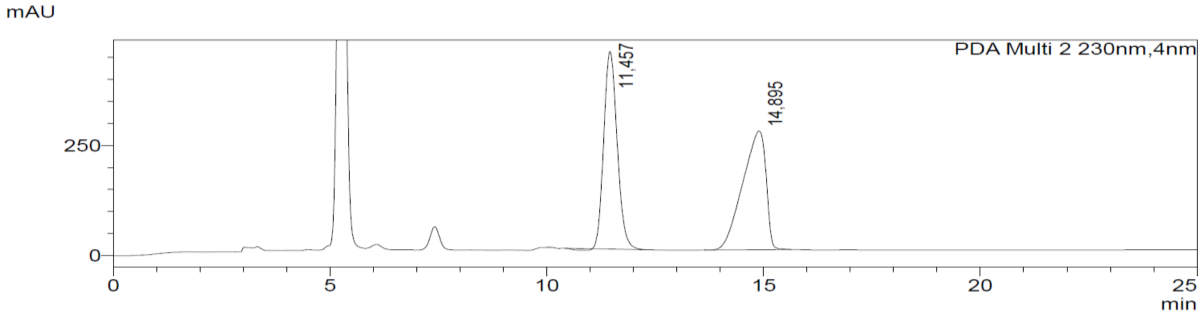

PDA Ch2 230nm

Peak#	Ret. Time	Name	Area	Height	Area%
1	11,457		9572832	447648	48,738
2	14,895		10068522	269159	51,262
Total			19641353	716807	100,000

Benzhydryl 4-hydroxy-2-methylene-4-phenylhexanoate (**8k**).

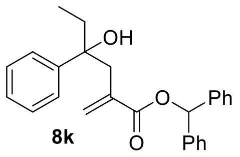

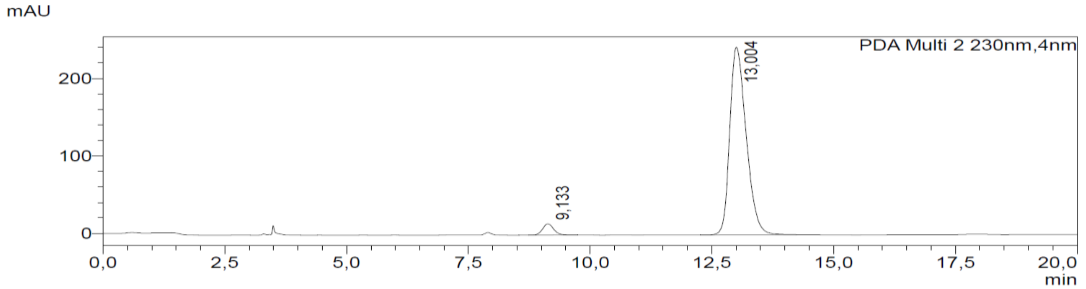

PDA Ch2 230nm

Peak#	Ret. Time	Name	Area	Height	Area%
1	9,133		228400	14390	3,861
2	13,004		5687530	241861	96,139
Total			5915930	256252	100,000

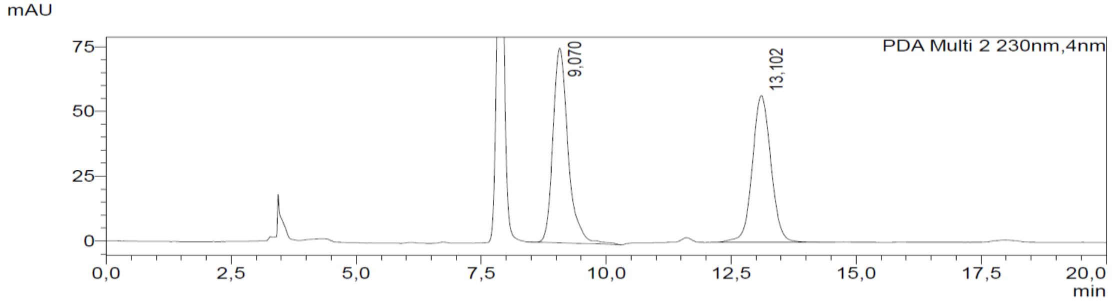

PDA Ch2 230nm

Peak#	Ret. Time	Name	Area	Height	Area%
1	9,070		1585320	75138	51,976
2	13,102		1464773	56576	48,024
Total			3050093	131714	100,000

Benzhydryl 4-hydroxy-2-methylene-4-phenylheptanoate (8l).

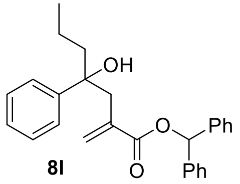

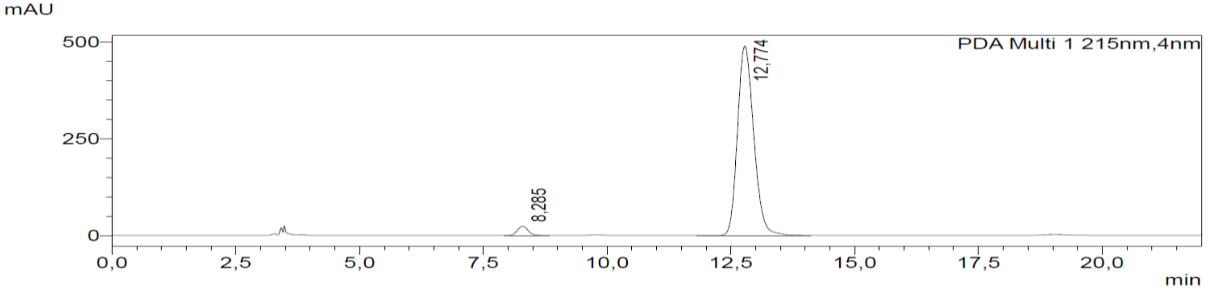

PDA Ch1 215nm

Peak#	Ret. Time	Name	Area	Height	Area%
1	8,285		354474	23706	3,076
2	12,774		11168767	489105	96,924
Total			11523240	512811	100,000

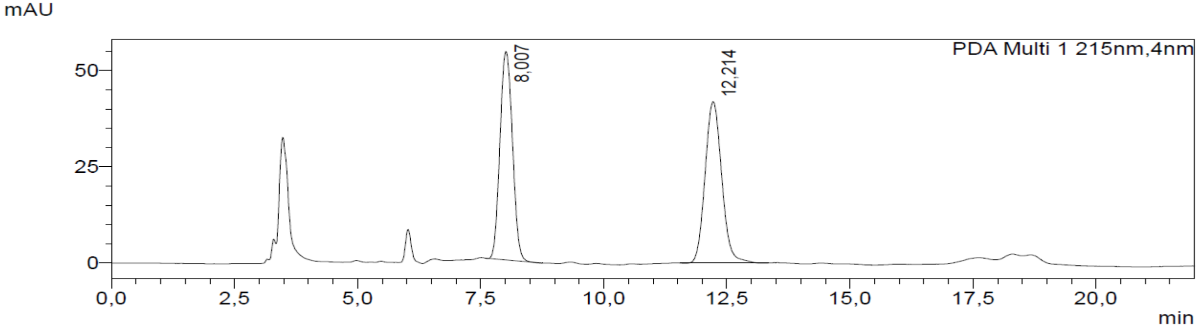

PDA Ch1 215nm

Peak#	Ret. Time	Name	Area	Height	Area%
1	8,007		956715	54167	49,276
2	12,214		984825	41885	50,724
Total			1941540	96052	100,000

Benzhydryl 2-[(1-hydroxy-1,2,3,4-tetrahydronaphthalen-1-yl)methyl]acrylate (**8m**).

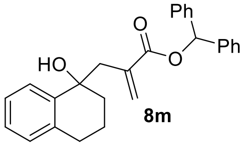

8m

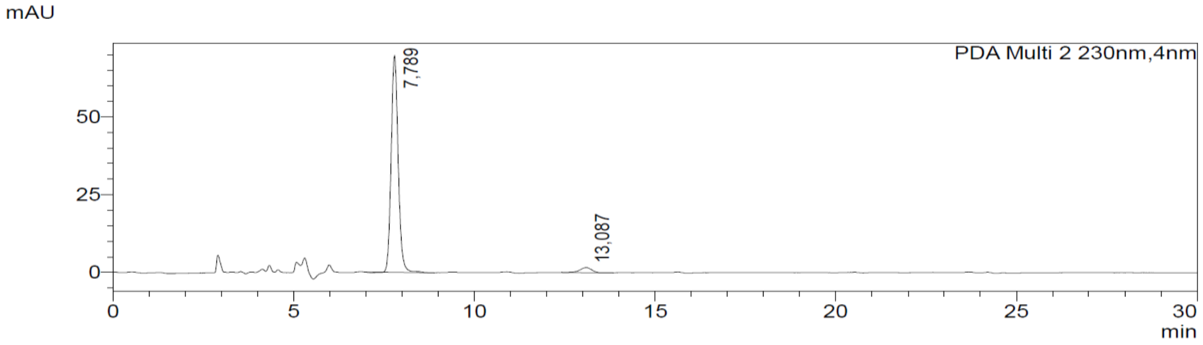

PDA Ch2 230nm

Peak#	Ret. Time	Name	Area	Height	Area%
1	7,789		943038	69718	96,422
2	13,087		34995	1616	3,578
Total			978034	71334	100,000

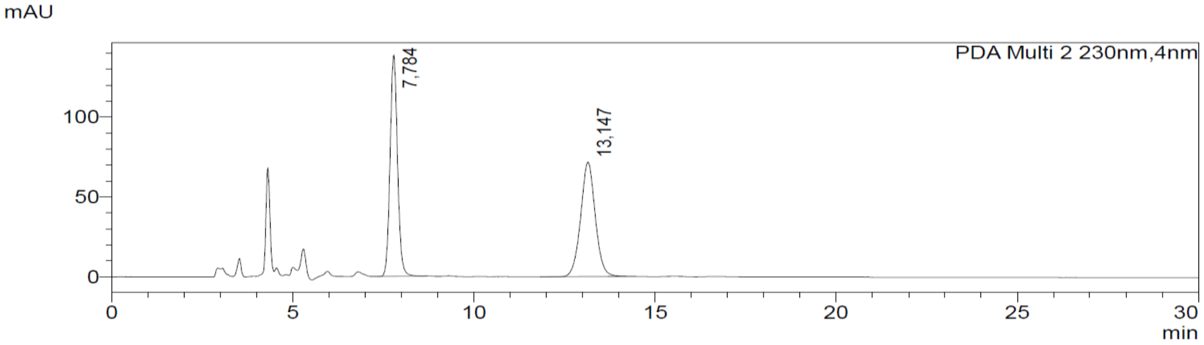

PDA Ch2 230nm

Peak#	Ret. Time	Name	Area	Height	Area%
1	7,784		1972749	138172	49,609
2	13,147		2003866	71551	50,391
Total			3976616	209723	100,000

Benzhydryl 2-[(4-hydroxychroman-4-yl)methyl]acrylate (**8n**).

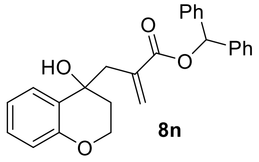

8n

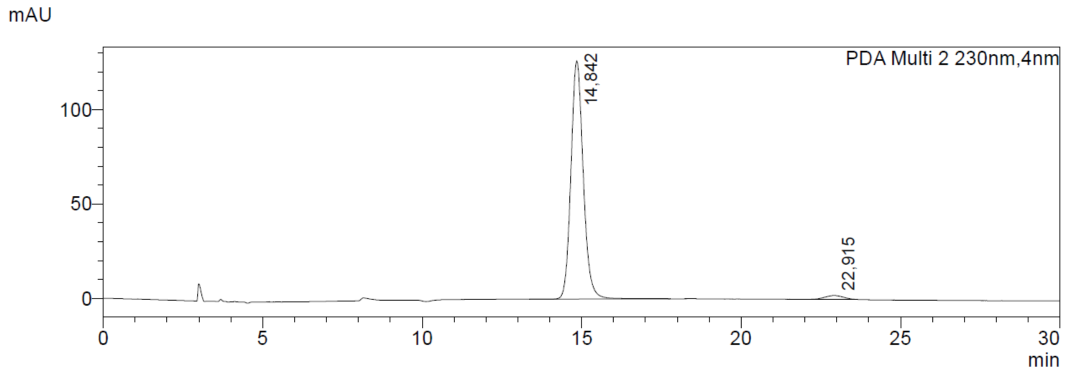

PDA Ch2 230nm					
Peak#	Ret. Time	Name	Area	Height	Area%
1	14,842		3396741	126149	97,552
2	22,915		85228	2103	2,448
Total			3481969	128251	100,000

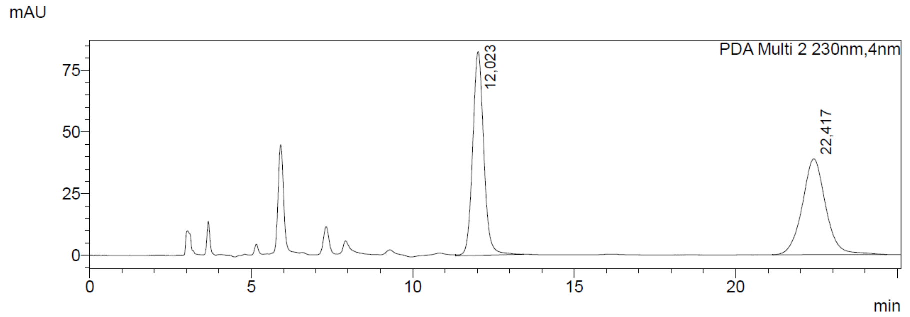

PDA Ch2 230nm					
Peak#	Ret. Time	Name	Area	Height	Area%
1	12,023		1972790	82764	50,164
2	22,417		1959903	38891	49,836
Total			3932693	121655	100,000

Benzhydryl 2-[(6-chloro-4-hydroxychroman-4-yl)methyl]acrylate (8o).

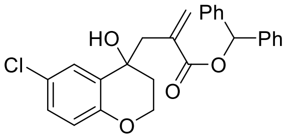

8o

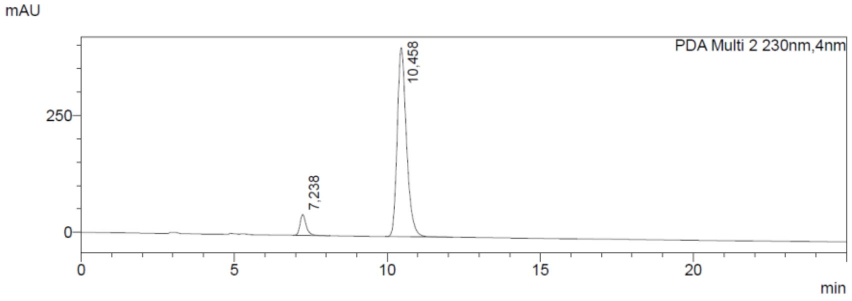

PDA Ch2 230nm					
Peak#	Ret. Time	Name	Area	Height	Area%
1	7,238		625368	44628	6,832
2	10,458		8528554	403699	93,168
Total			9153922	448328	100,000

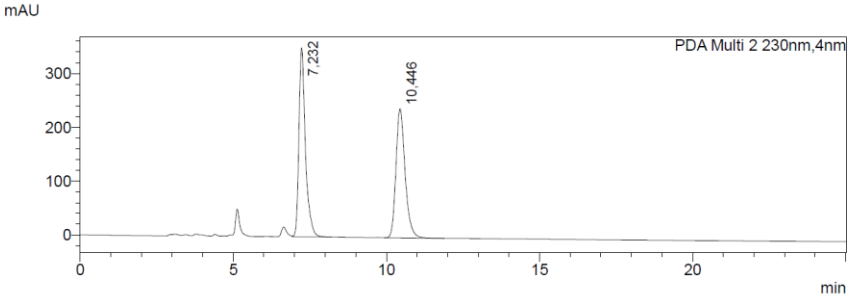

PDA Ch2 230nm					
Peak#	Ret. Time	Name	Area	Height	Area%
1	7,232		5181167	351625	51,027
2	10,446		4972605	240016	48,973
Total			10153772	591641	100,000

Benzhydryl (*E*)-4-hydroxy-4-methyl-2-methylene-6-phenylhex-5-enoate (8p).

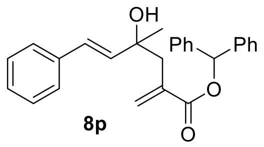

8p

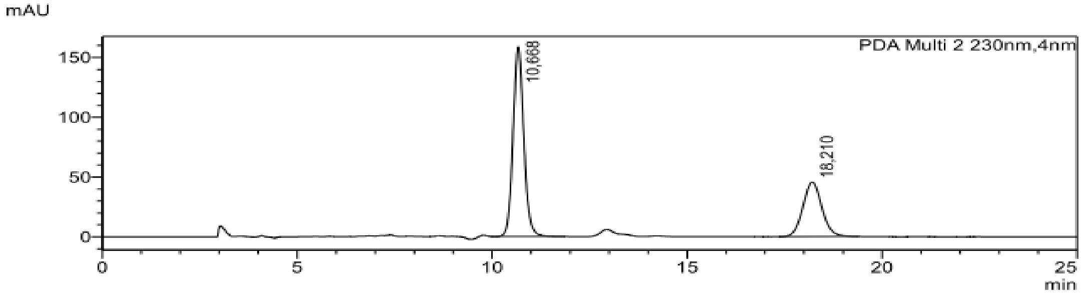

PDA Ch2 230nm

Peak#	Ret. Time	Name	Area	Height	Area%
1	10,668		3040225	158258	66,347
2	18,210		1542086	45566	33,653
Total			4582311	203824	100,000

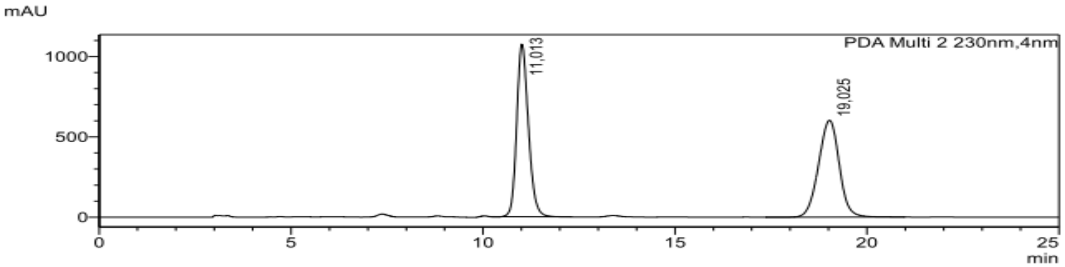

PDA Ch2 230nm

Peak#	Ret. Time	Name	Area	Height	Area%
1	11,013		22717518	1072154	50,085
2	19,025		22640822	602618	49,915
Total			45358340	1674772	100,000

Benzhydryl 4-(4-bromophenyl)-4-hydroxy-3-methyl-2-methylenepentanoate (**8q**).

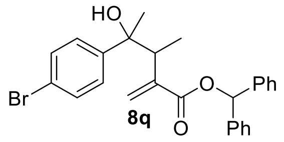

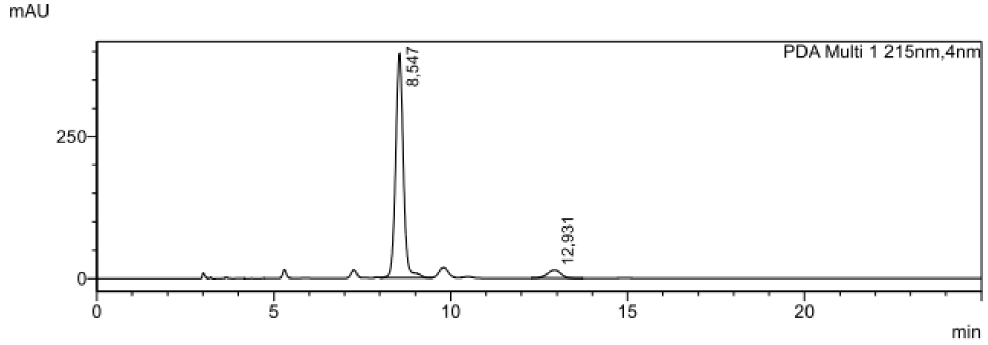

<Peak Table>

PDA Ch1 215nm

Peak#	Ret. Time	Name	Area	Height	Area%
1	8,547		6072079	394512	94,309
2	12,931		366421	14315	5,691
Total			6438501	408827	100,000

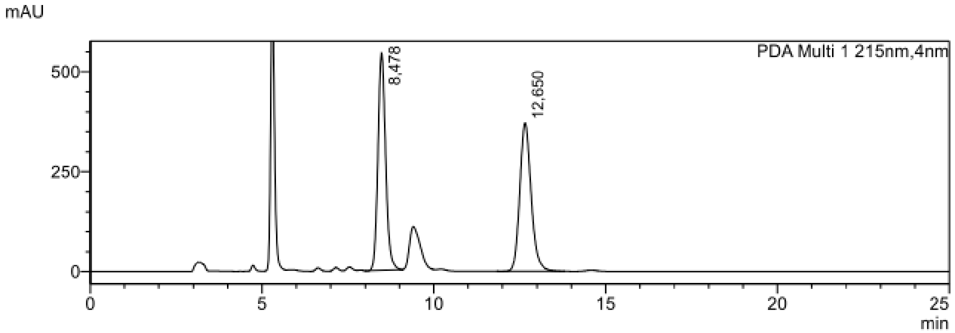

PDA Ch1 215nm

Peak#	Ret. Time	Name	Area	Height	Area%
1	8,478		8378765	543005	49,661
2	12,650		8493030	370275	50,339
Total			16871795	913280	100,000

Determination of Absolute Configuration.

*5-Methyl-3-methylene-5-phenyldihydrofuran-2(3H)-one (**10b**).*

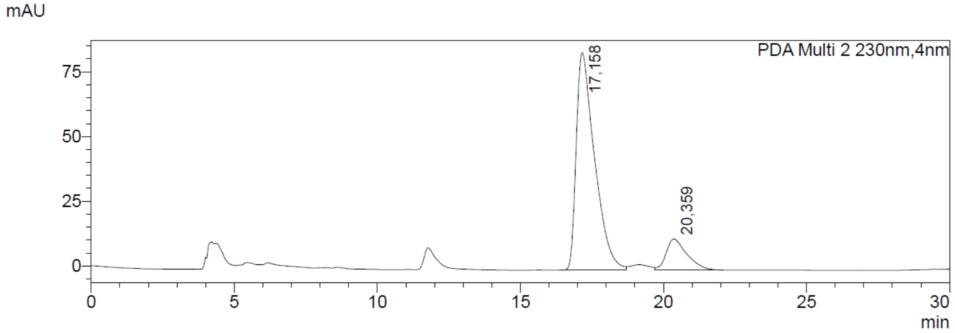

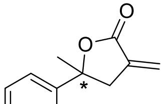

10b

PDA Ch2 230nm					
Peak#	Ret. Time	Name	Area	Height	Area%
1	17,158		3744938	84045	85,968
2	20,359		611287	12039	14,032
Total			4356225	96083	100,000

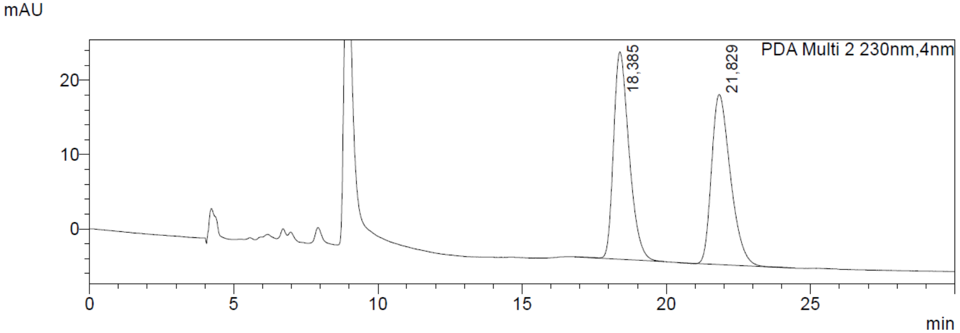

PDA Ch2 230nm					
Peak#	Ret. Time	Name	Area	Height	Area%
1	18,385		1046694	27899	50,021
2	21,829		1045824	22903	49,979
Total			2092518	50802	100,000

(R)-6-chloro-4'-methylene-3',4'-dihydro-5'H-spiro[chromane-4,2'-furan]-5'-one ***(10o)***.

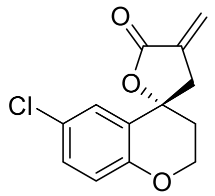

10o

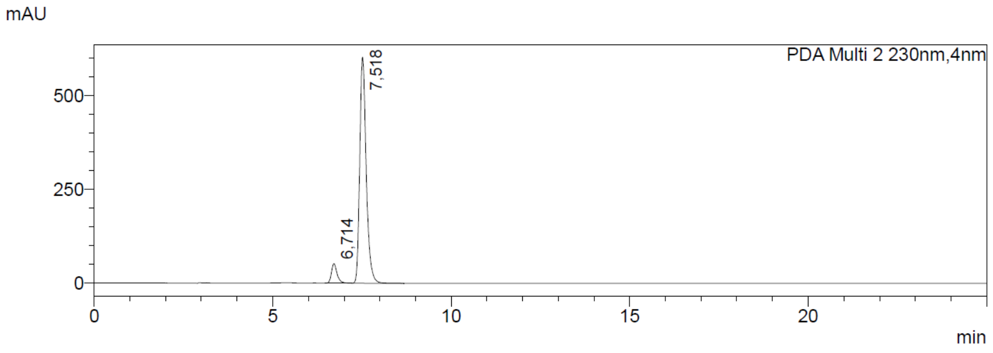

PDA Ch2 230nm

Peak#	Ret. Time	Name	Area	Height	Area%
1	6,714		576451	52125	7,028
2	7,518		7625795	603707	92,972
Total			8202245	655832	100,000

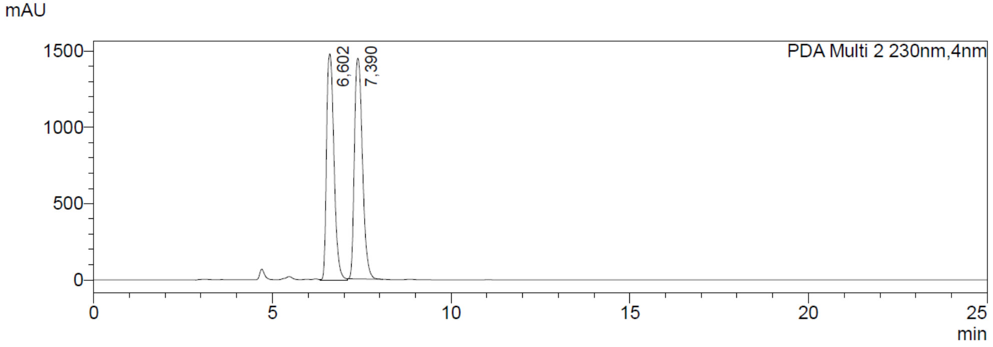

PDA Ch2 230nm

Peak#	Ret. Time	Name	Area	Height	Area%
1	6,602		21477900	1484039	48,715
2	7,390		22610871	1447664	51,285
Total			44088771	2931703	100,000

HPLC chromatograms of single crystals from X-Ray Analysis.

rac-**10o**-Cry1 [(*S*)-enantiomer crystallized with ee = 50%].

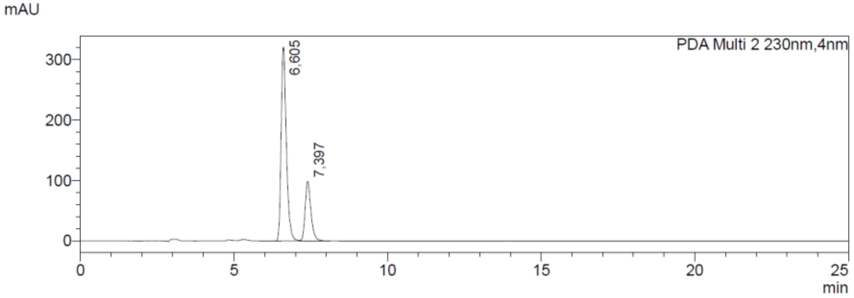

PDA Ch2 230nm

Peak#	Ret. Time	Name	Area	Height	Area%
1	6,605		3776463	321468	74,712
2	7,397		1278200	98175	25,288
Total			5054663	419643	100,000

rac-**10o**-Cry2 [(*S*)-enantiomer crystallized with ee = 98%].

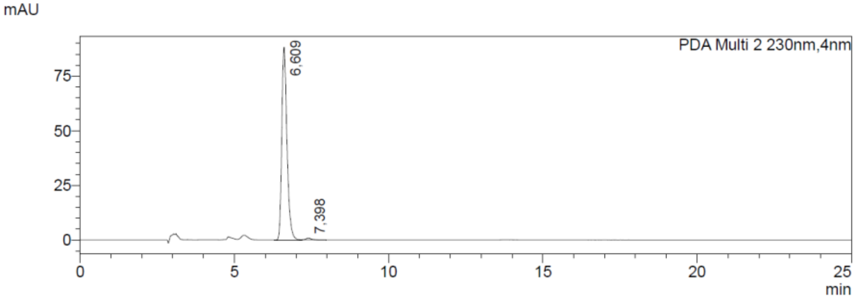

PDA Ch2 230nm

Peak#	Ret. Time	Name	Area	Height	Area%
1	6,609		1034765	88196	99,093
2	7,398		9473	783	0,907
Total			1044238	88979	100,000

(*R*)-**10o**-Cry1 [(*R*)-enantiomer crystallized with ee = > 99%].

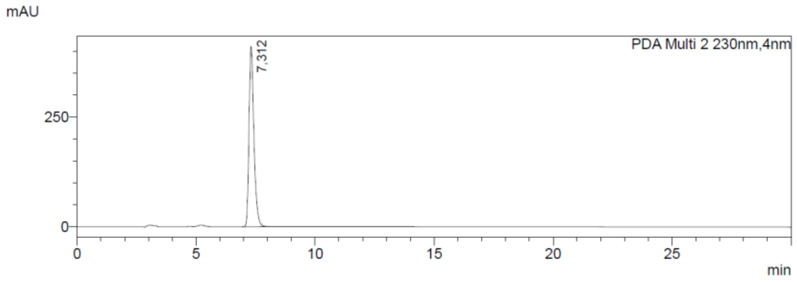

PDA Ch2 230nm

Peak#	Ret. Time	Name	Area	Height	Area%
1	7,312		5872117	411411	100,000
Total			5872117	411411	100,000

5-(4-bromophenyl)-4,5-dimethyl-3-methylenedihydrofuran-2(3H)-one (**10q**).

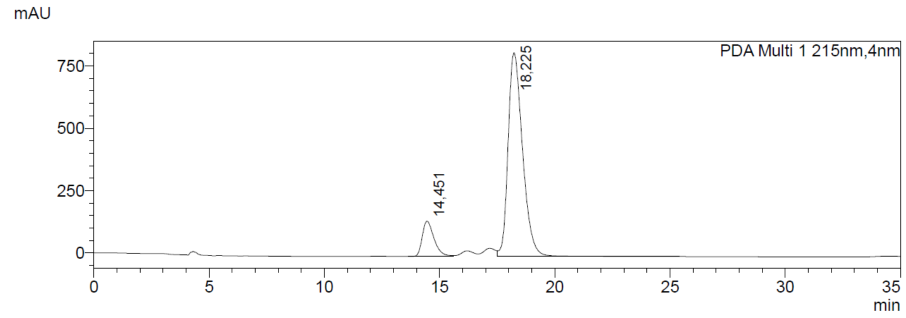

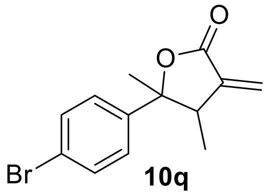

10q

PDA Ch1 215nm

Peak#	Ret. Time	Name	Area	Height	Area%
1	14,451		5083514	142039	12,421
2	18,225		35842772	817335	87,579
Total			40926285	959374	100,000

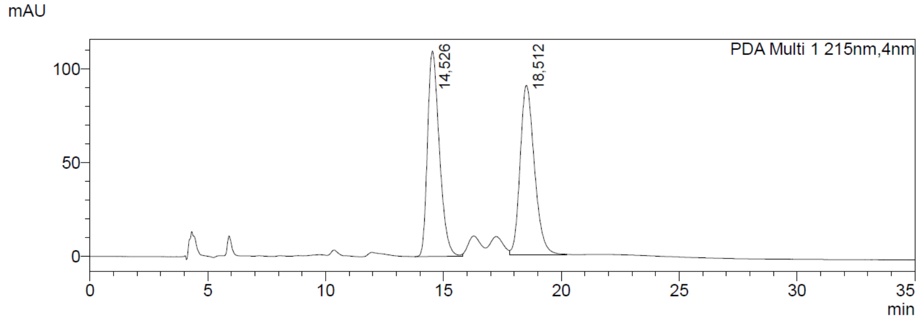

PDA Ch1 215nm

Peak#	Ret. Time	Name	Area	Height	Area%
1	14,526		3831006	109545	49,976
2	18,512		3834741	90504	50,024
Total			7665747	200049	100,000

4-[(4-bromophenyl)(hydroxy)methyl]-3-methylenedihydrofuran-2(3*H*)-one (**19**).

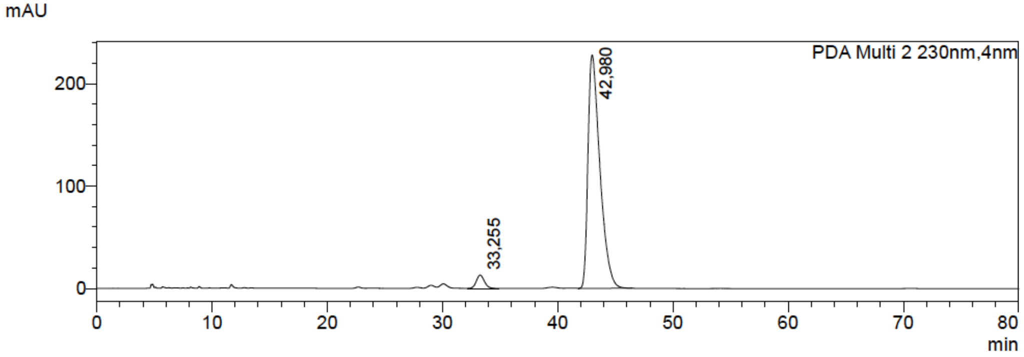

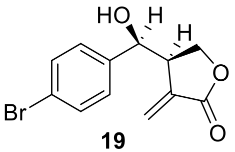

19

PDA Ch2 230nm

Peak#	Ret. Time	Name	Area	Height	Area%
1	33,255		627113	12995	3,684
2	42,980		16393222	228005	96,316
Total			17020335	241000	100,000

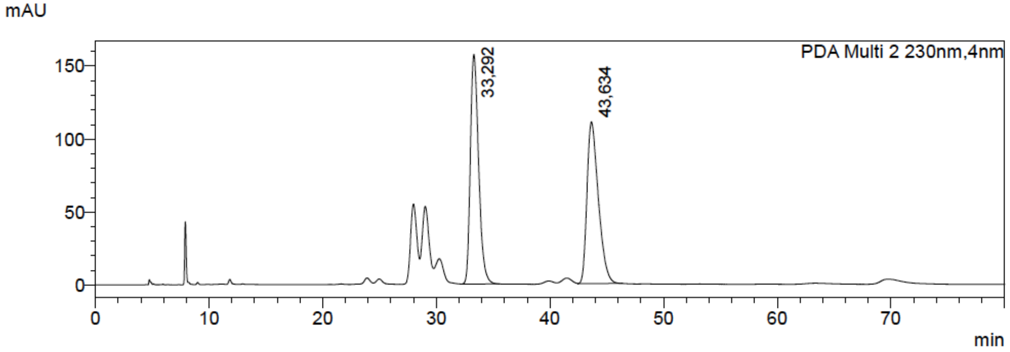

PDA Ch2 230nm

Peak#	Ret. Time	Name	Area	Height	Area%
1	33,292		7908705	157389	50,645
2	43,634		7707161	110835	49,355
Total			15615866	268224	100,000

4-[1-(4-bromophenyl)-1-hydroxyethyl]-3-methylenedihydrofuran-2(3H)-one (**20**).

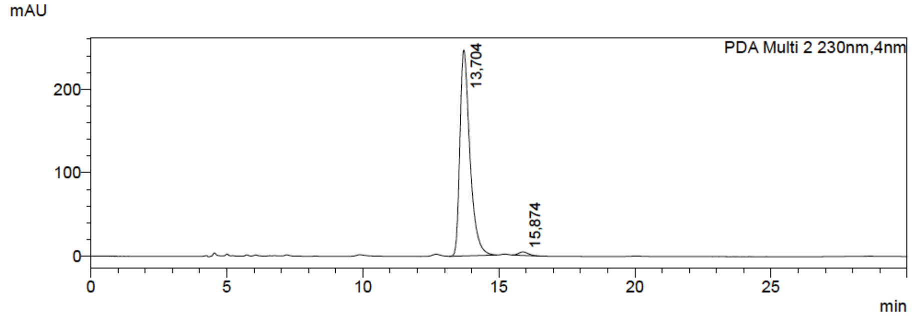

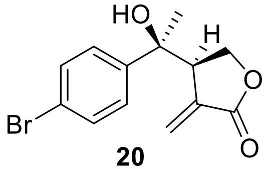

20

PDA Ch2 230nm

Peak#	Ret. Time	Name	Area	Height	Area%
1	13,704		6307761	246845	98,398
2	15,874		102701	4087	1,602
Total			6410462	250932	100,000

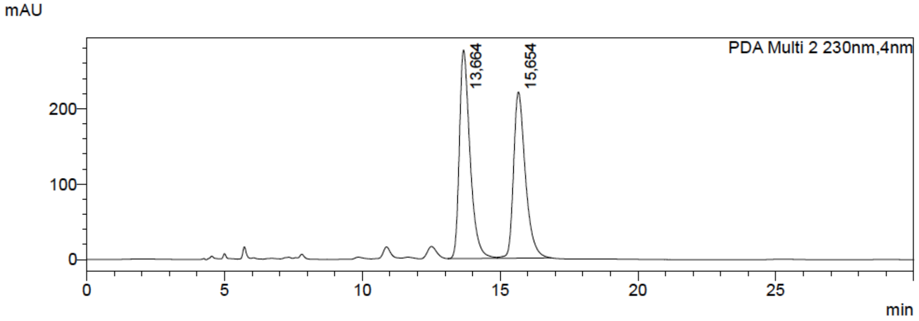

PDA Ch2 230nm

Peak#	Ret. Time	Name	Area	Height	Area%
1	13,664		7416671	276554	53,029
2	15,654		6569417	220360	46,971
Total			13986088	496915	100,000

N,*N*-dibenzyl-4-hydroxy-2-methylene-4-phenylpentanamide (8r).

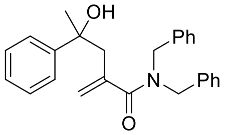

8r

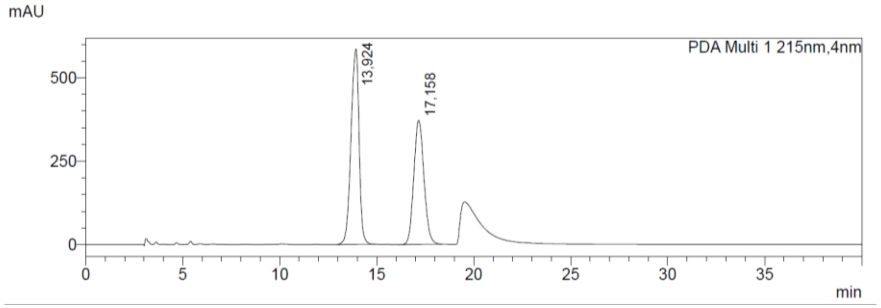

PDA Ch1 215nm

Peak#	Ret. Time	Name	Area	Height	Area%
1	13,924		17046867	585452	55,680
2	17,158		13569086	372000	44,320
Total			30615952	957452	100,000

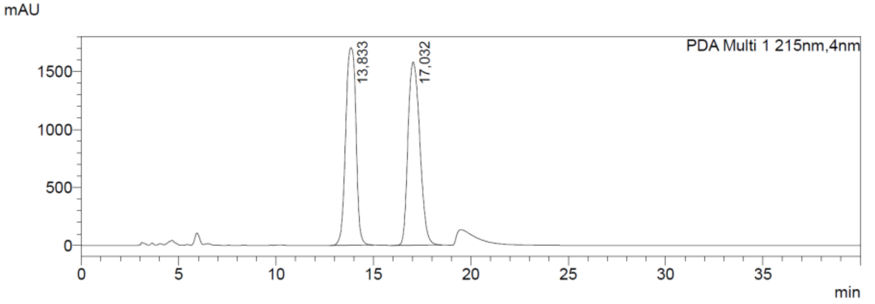

PDA Ch1 215nm

Peak#	Ret. Time	Name	Area	Height	Area%
1	13,833		62582180	1703936	48,343
2	17,032		66871423	1579411	51,657
Total			129453603	3283347	100,000

References.

1. Ishchenko, A. Y.; Yanik, S.; Rusanov, E. B.; Komarov, I. V.; Kirby, A. J., *Synthesis* **2015,** *47*, 367-367.
2. Jain, P.; Antilla, J. C., *J. Am. Chem. Soc.* **2010,** *132*, 11884-11886.
3. Suzuki, T.; Atsumi, J.-i.; Sengoku, T.; Takahashi, M.; Yoda, H., *J. Organomet. Chem.* **2010,** *695*, 128-136.
4. Boldrini, G. P.; Lodi, L.; Tagliavini, E.; Tarasco, C.; Trombini, C.; Umani-Ronchi, A., *J. Org. Chem.* **1987,** *52*, 5447-5452.
5. Eliel, E. L.; Bai, X.; Ohwa, M., *J. Chin. Chem. Soc.* **2000,** *47*, 63-70.
6. Garnier, J. M.; Robin, S.; Rousseau, G., *Eur. J. Org. Chem.* **2007,** *2007*, 3281-3291.
7. Sheldrick, G. M., *Acta Cryst.* **2008,** *A64*, 112-122.
8. Sheldrick, G. M., *Acta Cryst.* **2015,** *C71*, 3-8.
9. Flack, H., *Acta Cryst. A* **1983,** *39*, 876-881.
10. Johnson, C. K.; *ORTEP. Report ORNL-3794*, Oak Ridge National Laboratory: Tennessee, USA, **1965**.
11. Fuchs, M.; Schober, M.; Orthaber, A.; Faber, K., *Advanced Synthesis & Catalysis* **2013,** *355*, 2499-2505.
